# LP‐003, a novel high‐affinity anti‐IgE antibody for inadequately controlled seasonal allergic rhinitis: A multicenter, randomized, double‐blind, placebo‐controlled phase 2 clinical trial

**DOI:** 10.1002/clt2.70074

**Published:** 2025-06-22

**Authors:** Kai Guan, Shuang Liu, Yan Feng, Lisha Li, Xiaoming Zhu, Nai‐Chau‐Sun Bill, Haili Ma, Jie Yang, Cuicui Han, Heng Liu, Qingyu Wei, Haiyun Shi, Xueyan Wang

**Affiliations:** ^1^ Department of Allergy Beijing Key Laboratory of Precision Medicine for Diagnosis and Treatment on Allergic Diseases National Clinical Research Center for Dermatologic and Immunologic Diseases Peking Union Medical College Hospital Chinese Academy of Medical Sciences & Peking Union Medical College Beijing China; ^2^ Department of Otorhinolaryngology Head and Neck Surgery Shanxi Key Laboratory of Otorhinolaryngology Head and Neck Cancer The First Hospital Shanxi Medical University Taiyuan Shanxi China; ^3^ Department of Allergy Shengjing Hospital of China Medical University Shenyang Liaoning China; ^4^ Longbio Pharmaceutical Company Suzhou Jiangsu China; ^5^ Department of Allergy Beijing Shijitan Hospital Capital Medical University Beijing China

**Keywords:** anti‐IgE antibody, nasal symptom, RCT, seasonal allergic rhinitis, uncontrolled

## Abstract

**Background:**

Anti‐IgE therapy can serve as an option for inadequately controlled seasonal allergic rhinitis (SAR) patients. LP‐003, a novel anti‐IgE antibody, is being tested as an add‐on treatment for SAR. This trial aimed to evaluate whether LP‐003 is effective and safe for SAR.

**Methods:**

This placebo‐controlled double‐blind phase 2 randomized clinical trial was conducted in 17 hospitals in China. SAR patients whose symptoms were inadequately controlled despite first‐line treatment (nasal corticosteroids with or without oral antihistamine) in the previous two seasons were enrolled between July 6, 2023 and August 7, 2023. Participants were randomized in a ratio of 2:4:3 to receive subcutaneous injections of 100 mg LP‐003, 200 mg LP‐003 or placebo every 4 weeks for 2 doses. All patients received fluticasone propionate as standard‐of‐care (SoC). The main outcome was the mean total nasal symptom score (TNSS) during the peak pollen period (PPP). Secondary endpoints included a series of symptom and medication scores, quality of life assessments during PPP and pollen period (PP), immunogenicity and safety.

**Results:**

A total of 180 participants were randomly assigned. The LP‐003 + SoC treatment achieved a significantly lower TNSS compared with placebo + SoC (3.31 vs. 4.06, intergroup difference = −0.74, *p* = 0.0464). For key secondary outcomes, the LP‐003 group also achieved significantly lower daily nasal symptom and rescue medication use scores (3.54 vs. 4.42, intergroup difference = −0.88, *p* = 0.0352), and daily ocular symptom and rescue medication use scores (1.66 vs. 2.19, intergroup difference = −0.54, *p* = 0.0245) compared to the placebo group. The suppression of free IgE was prevalent and persistent. There was no statistically significant difference in adverse events and severe adverse events between LP‐003 and placebo groups.

**Conclusions:**

These findings support LP‐003 as a promising add‐on option to the SoC for patients with moderate to severe SAR. Fixed dosage regimen and extensive suppression of free‐IgE render it a cutting‐edge advantage.

## 1 INTRODUCTION

Seasonal allergic rhinitis (SAR) is one of the most prevalent allergic diseases around the world and shows a tendency to increase over time.^1^ With potential risk of developing asthma along with time^2^ or under special meteorological conditions,^3^ SAR exerts a heavy disease burden, especially in the context of climate change which has led to general extended pollen seasons and increased pollen production.^4^ The current cornerstones of AR treatment include allergen avoidance measures, pharmacotherapy and allergen immunotherapy (AIT).^5^ Since that environmental allergens are often difficult to completely avoid, and that not all AR patients are suitable candidates for AIT, pharmacotherapy remains the primary treatment approach in the current clinical setting.^6^ Current first‐line treatment for SAR includes intranasal corticosteroids, oral/intranasal antihistamines, etc.^7^ However, it was estimated that approximately 20%–35% of allergic rhinitis (AR) patients have insufficiently controlled symptoms despite current first‐line medical treatment.^8^, ^9^ Biologics have changed the treatment mode in many atopic diseases such as asthma, atopic dermatitis and food allergy. However, the application of biologics in patients with SAR is limited. Therefore, better therapeutic options are needed in addition to the currently available medications.

IgE plays a central role in the pathophysiology of many allergic diseases including AR, which exerts its effects mainly through interaction with two cellular receptors: the high‐affinity receptor FcεRI,^10^ mainly expressed on the surface of mast cells, basophils, and inflammatory dendritic cells, and the low‐affinity receptor CD23 (FcεRII/CD23),^11^ mainly expressed on the surface of B cells, triggering a wide variety of inflammatory responses including rapid release of preformed molecules (histamine, proteoglycans and proteases) and de novo synthesis of mediators, which results in clinical manifestations of type I hypersensitivity. The major mechanism of action of anti‐IgE antibody is to neutralize free IgE, preventing IgE‐mediated signal transduction and release of downstream mediators such as histamine.^12^ Therefore, the binding characteristics to human IgE are critical to the development of therapeutic anti‐IgE antibodies and prediction of its efficacy.

Omalizumab, the first approved anti‐IgE antibody, could effectively neutralize serum IgE regardless of allergen specificity, decrease FcεRI expression on mast cells, basophils, and dendritic cells, inhibiting FcεRI mediated allergic diseases and further reduce the risk of exacerbations.^13^ Omalizumab has shown efficacy in moderate‐to‐severe allergic asthma,^14^ chronic spontaneous/idiopathic urticaria,^15^ and food allergy.^16^ Previous studies have supported that omalizumab can reduce serum free IgE levels in SAR patients and provide clinical benefits.^17^, ^18^, ^19^, ^20^ Omalizumab as an add‐on standard of care (SoC, concomitant antihistamines and nasal corticosteroids) has also demonstrated efficacy in improving symptoms and quality of life in patients with inadequately controlled severe cedar pollinosis.^21^ However, Omalizumab's dosing regimen, which is based on total serum IgE levels and body weight, imposes a limitation on the IgE range, rendering a considerable proportion of patients with excessively high IgE levels unqualified for treatment. This creates a significant unmet medical need. In addition, Omalizumab has a rare (0.1%–0.2%) but severe, potentially life‐threatening side effect of anaphylaxis within 24 h after injection.^22^

Considering the advantages and disadvantages of the current anti‐IgE antibodies, a new anti‐IgE antibody should be generated with the following characteristics: higher‐IgE binding affinity, more potent FcεRI inhibition, satisfactory pharmacokinetics profile, fixed dosing regimen and reduced anaphylaxis risk. LP‐003 is a novel, humanized, anti‐IgE monoclonal antibody, which can block the binding of human IgE to FcεRI and FcεRII (CD23) receptors and inhibited the expression of the reporter gene. This study aimed to investigate the efficacy and safety of LP‐003 in treating moderate‐to‐severe SAR patients whose symptoms are inadequately controlled by SoC treatment.

## 2 METHODS

### 2.1 Generation of LP‐003

LP‐003 was generated using the hybridoma method. Briefly, antibody‐expressing B cells from immunized mice with recombinant IgE were fused with mouse myeloma cells to form hybridoma cells. Hybridoma cells expressing high‐affinity anti‐IgE antibodies were screened, and the genes encoding variable domain of the immunoglobulin heavy chain and variable domain of the immunoglobulin light chain of antibodies were cloned. After humanization, multiple mutations were introduced to the variable and constant regions. The abilities of LP‐003 in binding to human IgE and inhibition of IgE binding to IgE receptors were assessed and compared with Omalizumab (data provided in Appendix A and B). Membrane Proteome Array (MPA, Integral Molecular) was employed to test off‐target binding characteristics of LP‐003. A randomized, double‐blind, Phase I clinical study of LP‐003 was conducted.

### 2.2 Study design

The randomized, double‐blind, placebo‐controlled phase 2 clinical study (NCT06046391) was conducted at 17 centers located in Northern China. The study protocol is provided in Appendix C.

### 2.3 Participants

The eligible participants were moderate to severe SAR patients aged 18–65 years, with symptoms inadequately controlled despite first‐line treatment (nasal corticosteroids with or without oral antihistamine) in the previous 2 seasons. The diagnosis of SAR was made according to the 2022 version of Chinese guidelines for the diagnosis and treatment of AR,^23^ which include typical nasal symptoms, signs, and positive result for at least one allergen (consistent with clinical symptoms) in the skin prick test and/or serum‐specific IgE. Patients were excluded if they had perennial AR (SAR complicated with perennial AR and present with seasonal episodes may be included) or non‐allergic rhinitis; history of nasal or sinus surgery within 1 year; history of anti‐IgE antibody (e.g. Omalizumab) within 6 months; systemic usage of corticosteroids within 4 weeks; usage of intranasal glucocorticoids, mast cell membrane stabilizers, tricyclic antidepressants, leukotriene receptor antagonists, antihistamines within 1 week. The full list of entry and exclusion criteria is provided in Appendix D. The protocol and all documentation received ethics approval from the institutional review boards at each study site. The trial adhered to local regulations and Good Clinical Practice principles. Data analysis and reporting were performed in accordance with the Consolidated Standards of Reporting Trials reporting guidelines.

#### 2.3.1 Randomization and blinding

After the screening, eligible participants with moderate to severe SAR despite standard therapy in the previous two seasons were randomly assigned in a ratio of 2:4:3 to receive subcutaneous injections of 100 mg LP‐003, 200 mg LP‐003 or placebo every 4 weeks for 2 doses. The investigators and participants were blinded to assigned treatments during the study treatment period and clinical assessments. The placebo was indistinguishable in appearance from LP‐003.

#### 2.3.2 Procedures

**FIGURE 1:**
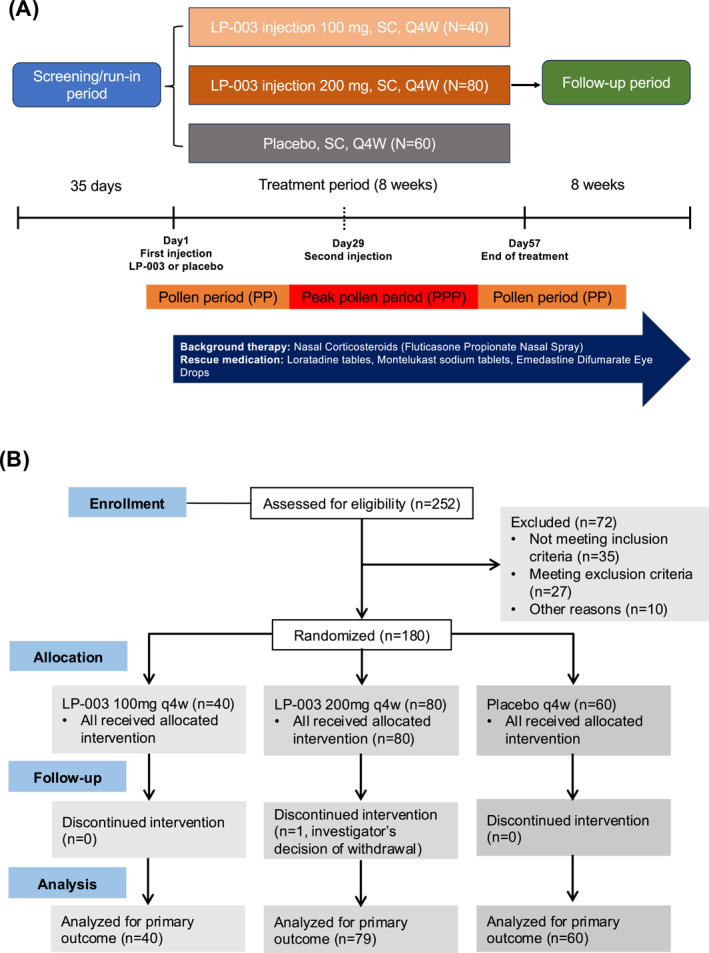
Overview of the study. (A) Trial design. (B) Trial profile.

The study comprised an up to 5‐week screening period and an 8‐week double‐blind treatment period (including visits for safety and efficacy measurements at day 1, 8, 15, 22, 29, 43, 57, 85, 113 for intensive sampling group and at day 1, 29, 57, 113 for non‐intensive sampling group). Participants received LP‐003 or placebo subcutaneously (every 4 weeks) for a total of 2 doses. Safety measures were also monitored. Blood and urine analyses and electrocardiographs were arranged during certain follow‐ups (detailed in Appendix C). All patients received concomitant nasal corticosteroids with/without antihistamines as SoC. Nasal fluticasone propionate (200 μg, once daily) was used throughout the treatment period irrespective of their symptoms. Loratadine tablets (oral, 10 mg/d, once daily), montelukast sodium tablets (oral, 10 mg/day, once daily) and emedastine difumarate eye drops (1 drop/day, twice daily) were permitted as rescue medication during the treatment period. An electronic diary was used to record nasal/ocular symptoms, and usage of rescue medications on a daily basis. The trial design is shown in Figure 1A.

#### 2.3.3 Endpoints

The primary endpoint was the mean daily total nasal symptom score (TNSS) during the peak pollen period (PPP). TNSS is calculated by summing the severity scores for sneezing, rhinorrhea, nasal congestion and nasal itching, with each symptom rated on a scale from 0 to 3, reflecting ascending severity. TNSS was chosen as the primary endpoint based on its frequent use in the design of clinical trials for AR.^21^, ^24^, ^25^ The total list of secondary endpoints is shown in Appendix C. Key secondary endpoints included mean daily nasal symptom and rescue medication scores (DNSMS) during PPP, mean daily ocular and rescue medication scores (DNOMS) during PPP, rescue medication usage during PP, and immunogenicity and safety. PPP was defined as the period encompassing three consecutive days before and after, each with a total pollen count of ≥300 pollen/1000 mm^2^, during the entire PP. PP was defined as the duration from the first day with a daily pollen count ≥20 total pollen/1000 mm^2^ to the last day with a count <20 total pollen/1000 mm^2^.

Rescue medication use scores refer to three symptom relief medications (loratadine tablets, montelukast sodium tablets and emedastine difumarate eye drops). Each medication used, regardless of dosage or frequency, receives 1 point, while non‐use is scored as 0 points. During the study, if participants find it difficult to tolerate nasal symptoms, they may use loratadine tablets. If symptoms remain uncontrolled, they should return to the hospital for a visit, and the investigator will decide, based on the participant's actual situation, whether to use montelukast sodium tablets. For relieving eye symptoms, participants may choose to use epinastine hydrochloride eye drops. Rescue medication should be discontinued once symptoms improve; during the use of rescue medication, continue using the investigational drug and standard concomitant therapy.

#### 2.3.4 Bioanalyses

The serum concentrations of LP‐003 and free‐IgE were determined by enzyme‐linked immunosorbent assay assays. Serum samples were collected at Days 15, 29, 43, 99 and 155 (end of study) and were tested for the presence of anti‐drug antibodies (ADAs) using a Meso Scale Discovery‐based homogenous bridging assay.

### 2.4 Statistics

The sample size was calculated according to the estimation of primary efficacy endpoint. Assuming *α* = 0.025 (one‐sided), a power (1 − *β*) of 0.8, and a non‐inferiority margin of 0, the mean difference in TNSS during the PPP between the treatment group and the control group (LP‐003 group minus placebo group) is −1, with a standard deviation of 2, and a treatment‐to‐control group ratio of 2:1. Using power analysis and sample size software (version 22), the calculated sample size was 146 participants (97 in the treatment group and 49 in the control group). Considering an approximate dropout rate of 20%, a total of 180 participants are required (120 in the treatment group and 60 in the control group).

Statistical analyses of efficacy endpoints were performed on the intention to treat analysis, which consisted of all randomized patients. The significance level of the efficacy endpoint was one‐sided 0.025. For the primary efficacy endpoint, the mean daily TNSS during the PPP for the two groups was compared using independent samples *t*‐tests to calculate the difference between the two groups and the upper limit of the 95% confidence interval. If the upper limit is < 0, then the superiority is established. According to the recommendations by ICH Harmonised Tripartite Guideline E9 (Statistical Principles For Clinical Trials), the U.S. Food & Drug Administration and European Medicines Agency guidance documents, imputation strategy was employed to enhance the robustness of the analytical framework. For missing values, multiple imputation methods are employed, considering group factors. The difference between treatment groups and placebo group was calculated by *t*‐tests. The secondary efficacy endpoints were analyzed using the same model as the primary outcome. The differences in rescue medication usage between groups were calculated by the chi‐square test. The assessment of safety was performed on all patients who received treatment at least once and had undergone safety assessment at least once. SAS 9.4 was used for the statistical analysis of clinical studies. Unless otherwise specified, the significance level of statistical tests other than efficacy endpoints is two‐sided 0.05.

## 3 RESULTS

### 3.1 Baseline characteristics of participants

Between July 6, 2023 and August 7, 2023, a total of 252 participants were screened, after screening 180 adult patients were randomized to receive LP‐003 100 mg (*n* = 40) or LP‐003 200 mg (*n* = 80) or placebo (*n* = 60). The flowchart of the phase 2 trial and design of the study procedure are shown in Figure 1B. As shown in Table 1, demographics and clinical characteristics were generally balanced between the treatment groups and the placebo group.

**TABLE 1:** Baseline characteristics and pollen season exposure of participants.

Characteristics	LP‐003			Placebo (*N* = 60)
	100 mg (*N* = 40)	200 mg (*N* = 80)	Total (*N* = 120)	
Age, mean (SD), y	37.0 (11.0)	36.8 (10.6)	36.9 (10.7)	39.1 (9.6)
Age, median (Q1–Q3), y	37.0 (28.5–43.0)	37.0 (29.0–44.0)	37.0 (29.0–43.0)	37.5 (32.0–44.5)
Sex, *n* (%)				
Men	18 (45.0)	31 (38.8)	49 (40.8)	34 (56.7)
Women	22 (55.0)	49 (61.3)	71 (59.2)	26 (43.3)
Weight, mean (SD), kg	68.1 (11.6)	65.6 (13.2)	66.4 (12.7)	72.9 (14.3)
Weight, median (Q1–Q3), kg	66.3 (60.5–75.5)	61.9 (55.0–74.2)	63.9 (56.5–74.7)	74.5 (62.3–81.0)
Height, mean (SD), cm	167.9 (7.9)	166.5 (8.2)	167.0 (8.1)	169.9 (8.8)
Height, median (Q1–Q3), cm	168.5 (160.5–172.5)	165.0 (160.0–172.5)	165.5 (160.0–172.5)	170.0 (163.0–177.0)
Baseline total nasal symptom score, mean (SD)	4.07 (2.28)	4.55 (2.40)	4.39 (2.36)	4.78 (3.04)
Free IgE IU/mL, no. (%)				
≤150	18 (45.0)	47 (58.8)	55 (54.2)	31 (51.7)
>150	22 (55.0)	33 (41.3)	65 (45.8)	29 (48.3)
Baseline free IgE level, mean (SD), IU/mL				
≤150	77.4 (38.5)	64.9 (37.8)	68.3 (38.1)	60.3 (40.1)
>150	629.5 (875.5)	580.7 (571.2)	600.2 (701.5)	650.0 (836.6)
Exposure to pollen season, mean (SD), d	77.9 (15.3)	76.6 (14.5)	77.0 (14.7)	78.2 (15.5)
Exposure to peak pollen period, mean (SD), d	13.7 (8.9)	11.2 (8.2)	12.0 (8.5)	12.2 (8.3)

*Note*: Data are presented as *n* (%), unless otherwise indicated.

### 3.2 Efficacy

**FIGURE 2:**
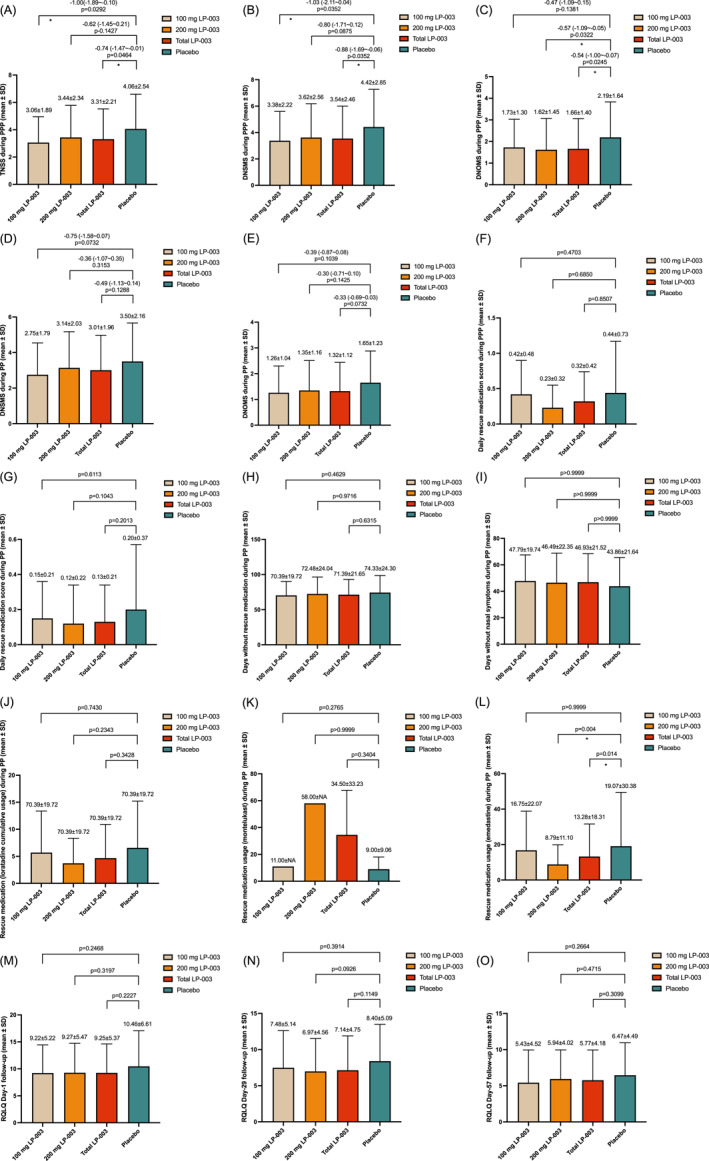
Efficacy endpoints of this trial. (A) Mean total nasal symptom score (TNSS) in LP‐003 and placebo group during peak pollen period (PPP). (B) Mean daily nasal symptom and rescue medication score (DNSMS) during PPP. (C) Mean daily ocular and rescue medication scores (DNOMS) during PPP. (D) Mean DNSMS during PP. (E) DNOMS during PP. (F) Daily rescue medication score during PPP. (G) Daily rescue medication score during PP. (H) Days without rescue medication during PP. (I) Days without symptoms during PP. (J) Rescue medication (loratadine cumulative usage) during PP. (K) Rescue medication (montelukast cumulative usage) during PP. (L) Rescue medication (emedastine cumulative usage) during PP. (M) Rhinoconjunctivitis Quality of Life Questionnaire (RQLQ) at Day‐1 follow up. (N) RQLQ at Day‐29 follow up. (O) RQLQ at Day‐57 follow up. DNOMS, daily ocular and rescue medication scores. DNSMS, daily nasal symptom and rescue medication scores. PP, pollen period; PPP, peak pollen period; RQLQ, Rhinoconjunctivitis Quality of Life Questionnaire; SD, standard deviation. The intergroup difference was calculated using *t*‐test, with statistically significant differences (*p* < 0.05) marked by asterisk (*).

For the primary endpoint, the average TNSS during PPP was significantly lower in LP‐003 treatment group than in the placebo group (3.31 vs. 4.06, intergroup difference = −0.74, *p* = 0.0464). The difference between LP‐003 group and placebo group (−0.74) exceeded the prespecified minimal clinically important difference of 0.55 for AR.^26^ For the key secondary endpoints, LP‐003 treatment group also significantly reduced the DNSMS (3.54 vs. 4.42, intergroup difference = −0.88, *p* = 0.0352) and DNOMS (1.66 vs. 2.19, intergroup difference = −0.54, *p* = 0.0245) compared to the placebo group. During PP, the usage of loratadine tablets in the LP‐003 group was significantly lower than in the placebo (22.5% vs. 41.7%, *p* = 0.0075). The usage of emedastine difumarate eye drops in LP‐003 group was also significantly lower than in the placebo group (26.7% vs. 45%, *p* = 0.0135). The above results as well as the comparisons between each LP‐003 dose group and the placebo group are shown in Table 2 and Figure 2.

**TABLE 2:** Efficacy endpoints of LP‐003.

Variable	Placebo	LP‐003, total	LP‐003, 100 mg group	LP‐003, 200 mg group	*p* value between LP‐003 100/200 mg
Primary endpoint					
Total nasal symptom scores (TNSS) during the peak pollen season (PPP)^a^					
No. of patients analyzed, n	60	119	40	79	0.3825
Average score, mean (SD)	4.06 (2.54)	3.31 (2.21)	3.06 (1.89)	3.44 (2.34)	
Difference between treatment group versus placebo, mean (95% CI)	N/A	−0.74 (−1.47 to −0.01)	−1.00 (−1.89 to −0.10)	−0.62 (−1.45 to 0.21)	
*p* value versus placebo	N/A	***0.0464***	***0.0292***	0.1427	
Secondary endpoints					
Daily nasal symptom and rescue medication use scores (DNSMS) during the peak pollen season (PPP)^a^					
No. of patients analyzed, n	60	120	40	80	0.6202
Average score, mean (SD)	4.42 (2.85)	3.54 (2.46)	3.38 (2.22)	3.62 (2.56)	
Difference between treatment group versus placebo, mean (95% CI)	N/A	−0.88 (−1.69 to −0.06)	−1.03 (−2.11 to 0.04)	−0.80 (−1.71 to 0.12)	
*p* value versus placebo	N/A	0.0352	0.0581	0.0875	
Daily ocular symptom and rescue medication use scores (DNOMS) during the peak pollen season (PPP)^a^					
No. of patients analyzed, n	60	120	40	80	0.7060
Average score, mean (SD)	2.19 (1.64)	1.66 (1.40)	1.73 (1.30)	1.62 (1.45)	
Treatment group versus placebo, mean (95%CI)	N/A	−0.54 (−1.00 to −0.07)	−0.57 (−1.09 to −0.05)	−0.47 (−1.09 to 0.15)	
*p* value versus placebo	N/A	0.0245	0.1381	0.0322	
Daily nasal symptom and rescue medication use scores (DNSMS) during the entire pollen period (PP)^a^					
No. of patients analyzed, n	60	120	40	80	0.3063
Average score, mean (SD)	3.50 (2.16)	3.01 (1.96)	2.75 (1.79)	3.14 (2.03)	
Treatment group versus placebo, mean (95%CI)	N/A	−0.49 (−1.13 to 0.14)	−0.75 (−1.58 to 0.07)	−0.36 (−1.07 to 0.35)	
*p* value versus placebo	N/A	0.1288	0.0732	0.3153	
Daily ocular symptom and rescue medication use scores (DNOMS) during the entire pollen period (PP)^a^					
No. of patients analyzed, n	60	120	40	80	0.6802
Average score, mean (SD)	1.65 (1.23)	1.32 (1.12)	1.26 (1.04)	1.35 (1.16)	
Treatment group versus placebo, mean (95% CI)	N/A	−0.33 (−0.69 to 0.03)	−0.39 (−0.87 to 0.08)	−0.30 (−0.71 to 0.10)	
*p* value versus placebo	N/A	0.0732	0.1039	0.1425	
Daily rescue medication score during the peak pollen season (PPP)					
No. of patients analyzed, n	60	120	40	80	0.2285
Average score, mean (SD)	0.44 (0.73)	0.32 (0.42)	0.42 (0.48)	0.23 (0.32)	
*p* value versus placebo	N/A	0.8507	0.4702	0.6850	
Daily rescue medication score during the entire pollen period (PP)					
No. of patients analyzed, n	60	120	40	80	0.3390
Average score, mean (SD)	0.20 (0.37)	0.13 (0.21)	0.15 (0.21)	0.12 (0.22)	
*p* value versus placebo	N/A	0.2013	0.6113	0.1043	
Days without rescue medication during the entire pollen period (PP)					
No. of patients analyzed, n	60	120	40	80	0.5479
Average score, mean (SD)	74.33 (24.30)	71.39 (21.65)	70.39 (19.72)	72.48 (24.04)	
*p* value versus placebo	N/A	0.6315	0.4629	0.9716	
Rescue medication usage during the entire pollen period (PP), loratadine tablets					
No. of patients analyzed, n	60	120	40	80	0.5524
Cumulative usage, mean (SD)	6.58 (8.64)	4.67 (6.24)	5.69 (7.69)	3.71 (4.63)	
*p* value versus placebo	N/A	0.3428	0.7430	0.2343	
Percentage of user, mean (SD)	25 (41.7)	27 (22.5)	13 (32.5)	14 (17.5)	0.0636
*p* value versus placebo	N/A	***0.0075***	0.3549	***0.0016***	
Rescue medication usage during the entire pollen period (PP), accumulation of montelukast sodium tablets					
No. of patients analyzed, n	60	120	40	80	N/A
Cumulative usage, mean (SD)	9.00 (9.06)	34.50 (33.23)	11.00 (NA)	58.00 (NA)	
*p* value versus placebo	N/A	0.3404	>0.9999	0.2765	
Percentage of user, mean (SD)	4 (6.7)	2 (1.7)	1 (2.5)	1 (1.3)	>0.9999
*p* value versus placebo	N/A	0.1864	0.6396	0.2117	
Rescue medication usage during the entire pollen period (PP), accumulation of emedastine difumarate eye drops					
No. of patients analyzed, n	60	120	40	80	0.3586
Cumulative usage, mean (SD)	19.07 (30.38)	13.28 (18.31)	16.75 (22.07)	8.79 (11.10)	
*p* value versus placebo	N/A	0.5010	0.8798	0.3062	
Percentage of user, mean (SD)	27 (45.0)	32 (26.7)	18 (45.0)	14 (17.5)	0.0013
*p* value versus placebo	N/A	***0.014***	>0.9999	***0.0004***	
Rhinoconjunctivitis Quality of Life Questionnaire (RQLQ) during the entire pollen period (PP)^a^					
No. of patients analyzed, n	60	120	40	80	0.9578
Day 1 follow‐up, mean (SD)	10.46 (6.61)	9.25 (5.37)	9.22 (5.22)	9.27 (5.47)	
*p* value versus placebo	N/A	0.2227	0.3197	0.2468	
Day 29 follow‐up, mean (SD)	8.40 (5.09)	7.14 (4.75)	7.48 (5.14)	6.97 (4.56)	0.5893
*p* value versus placebo	N/A	0.1149	0.3914	0.0926	
Day 57 follow‐up, mean (SD)	6.47 (4.49)	5.77 (4.18)	5.43 (4.52)	5.94 (4.02)	0.5338
*p* value versus placebo	N/A	0.3099	0.2664	0.4715	
Days without nasal symptoms during the entire pollen period (PP)					
No. of patients analyzed, n	60	120	40	80	>0.9999
Average days, mean (SD)	43.86 (21.64)	46.93 (21.52)	47.79 (19.74)	46.49 (22.35)	
*p* value versus placebo	N/A	>0.9999	>0.9999	>0.9999	

*Note*: All data were analyzed using *t*‐tests (normally distributed data) or Wilcoxon rank‐sum tests (non‐normally distributed data). For the primary endpoint (TNSS during the peak pollen season), the nasal symptom scores were calculated as follows: sum the daily averages of each participant, then divide by the number of peak pollen days. For missing values and imputation strategies, multiple imputation methods are employed, considering group factors, with imputation conducted 30 times. DNSMS was calculated as follows: (daily nasal symptom scores + daily rescue medication use score)/number of peak pollen days. Rescue medication use scores: refer to two nasal symptom relief medications (loratadine tablets and montelukast sodium tablets). The usage of each rescue medication, regardless of dosage and frequency, is scored as 1 point, and scores 0 points if no medication is used. For missing values and imputation strategies, multiple imputation methods are employed, considering group factors, with imputation conducted 20 times. DNSMS was calculated as follows: Ocular symptom and rescue medication use scores: (daily ocular symptom scores + daily rescue medication use score)/number of peak pollen days. Rescue medication use scores: refer to two relief medications (loratadine tablets and emedastine difumarate eye drops). The usage of each rescue medication, regardless of dosage and frequency, is scored as 1 point, and scores 0 points if no medication is used. For missing values and imputation strategies, multiple imputation methods are employed, considering group factors, with imputation conducted 20 times. Daily rescue medication score refers to the usage score of two nasal symptom‐relieving medications (Loratadine tablets and Montelukast sodium tablets) and one ocular symptom‐relieving medication (Emedastine fumarate eye drops). The usage of each rescue medication, regardless of dosage and frequency, is scored as 1 point. That is, the number of medications used by the participant each day corresponds to the score, with 0 points assigned for non‐use. Days without rescue medication refers to the days when neither nasal symptom‐relieving medications (Loratadine tablets and Montelukast sodium tablets) nor ocular symptom‐relieving medications (Emedastine fumarate eye drops) were used, without considering imputation strategies. Days without nasal symptoms is defined as the number of days when all nasal symptom scores were ≤1. For the RQLQ analysis, the mean score of the 7 items is calculated by summing the individual item scores and dividing by the total number of items, without considering imputation strategies. The bold‐italic formatting is used to highlight statistically significant *p*‐values.

Abbreviations: DNOMS, daily ocular symptom and rescue medication use scores; DNSMS, daily nasal symptom and rescue medication use scores; N/A, not applicable; PP, pollen period; PPP, peak pollen period; RQLQ, rhinoconjunctivitis Quality of Life Questionnaire; TNSS, total nasal symptom score.

^a^ Data were analyzed using independent samples *t*‐tests, other data were analyzed using rank sum test.

### 3.3 Bioanalyses of free‐IgE suppression and immunogenicity

Biomarker analyses showed that serum free IgE was significantly reduced in LP‐003 group. In 100 mg Q4W group, free‐IgE in 90% of patients (36/40) was suppressed to below lower limit of quantification (LLOQ) on day 29. This proportion persisted until day 57. The mean free‐IgE level in four patients whose free‐IgE was still detectable was 135 IU/mL on day 29 and 73 IU/mL on day 57. In 200 mg Q4W group, free‐IgE in 98.5% of patients (79/80) was suppressed below LLOQ at day 29 and this suppression persisted until day 57. The only patient with free‐IgE still in detectable range had free‐IgE of 29 IU/mL on day 29 and 20 IU/mL on day 57.

The ADA in 100 and 200 mg groups was 2.5% (1/40) and 3.8% (3/80), respectively at day 29 and 5.0% (2/40) and 8.8% (7/80), respectively at day 57. Surprisingly, there was also pre‐existing ADA in those subjects before LP‐003 injection (2.5% and 3.8%, respectively at baseline), suggesting that the detected ADA might target the constant part of the LP‐003.

### 3.4 Safety profiles

Adverse events during the trial are summarized in Table 3. The frequency of AE between LP‐003 group and placebo groups was similar. There were 1 serious adverse event (SAE) in LP‐003 group and 2 SAEs in the placebo group. The only SAE that led to discontinuation occurred in the LP‐003 group, in which the patient had a history of intermittent deafness and was advised to discontinue when similar symptoms developed during the trial.

**TABLE 3:** Summary of adverse events.

	LP‐003 (N = 120)	Placebo (N = 60)	*p* value
Death	0	0	
SAEs	1 (0.8)	2 (3.3)	0.217
AEs leading to discontinuation	1 (0.8)	0	0.478
Any AEs	50 (41.7)	24 (40.0)	0.830
Most frequently occurring AEs (≥2% in either group)			
UA increased	7 (5.8)	3 (5.0)	0.818
Upper respiratory tract infection	4 (3.3)	2 (3.3)	1.000
Fever	4 (3.3)	0	0.153
Nasopharyngitis	3 (2.5)	2 (3.3)	0.748
ALT increased	3 (2.5)	2 (3.3)	0.748
TBIL increased	3 (2.5)	1 (1.7)	0.721
Urinary tract infection	3 (2.5)	1 (1.7)	0.721
Urticaria	3 (2.5)	0	0.217
Joint pain	3 (2.5)	0	0.217
Hyperuricemia	3 (2.5)	0	0.217
Headache	3 (2.5)	0	0.217

*Note*: Date was presented as *n* (%). The *p*‐value is calculated using Fisher's exact test.

Abbreviations: AE, Adverse event; ALT, alanine transaminase; SAE, serious adverse event; TBIL, total bilirubin; UA, uric acid.

## 4 DISCUSSION

As the first registered domestic anti‐IgE antibody in China, this study supported LP‐003 as a novel anti‐IgE antibody with unique pharmacological properties, and confirmed its efficacy and safety through a multicenter, randomized, double‐blind, placebo‐controlled phase 2 clinical trial. The high affinity of LP‐003 enables the possibility of fixed‐dose administration, which is more convenient and potentially cost‐effective. Overall, the unique pharmacological characteristics endow this novel anti‐IgE antibody with the potential to challenge the currently available treatment options.

The discovery of IgE and its role in allergic diseases has led to the development of anti‐IgE therapies. Omalizumab, the first approved anti‐IgE antibody, binds to the CH3 domain of human IgE, directly blocks IgE binding with high‐affinity type‐I receptors (FcεRI) and in‐sterically inhibits its binding to low affinity type II receptors (FcεRII/CD23).^27^ Compared to traditional pharmacological treatments, anti‐IgE therapy offers higher efficacy, convenience, and fewer side effects. However, the insufficient affinity to IgE of Omalizumab requires dosage adjustment based on serum IgE levels and body weight, which makes patients with higher IgE level and greater body weight unqualified for the treatment. Moreover, the risk of life‐threatening anaphylaxis has somewhat limited the convenient use of Omalizumab. These limitations have driven the development of next‐generation anti‐IgE antibodies to address unmet medical needs.

Our study demonstrated significant clinical efficacy for LP‐003 compared with placebo. Given that the study aims to explore the anti‐IgE effects of LP‐003 in controlling SAR symptoms during the pollen season, the timing of administration is set prior to the pollen peak (assuming circulating IgE has not yet fully bound to receptors). Consequently, participants may not have reached their highest TNSS values at the time of receiving the first dose of the investigational drug. Therefore, the absolute TNSS level during the subsequent pollen peak, rather than the change in TNSS, serves as the primary endpoint of the study. This study design is also consistent with the Phase 3 design of Omalizumab in SAR.^21^ Although the 200 mg LP‐003 group did not demonstrate statistically significant differences in primary endpoints compared to the placebo group, it exhibited clinically meaningful efficacy in DOSMS and rescue medication usage (loratadine tablets and emetine fumarate eye drops). Furthermore, a dose‐dependent response was observed between the 100 and 200 mg groups for these above outcomes. The observed discrepancy between primary and secondary endpoints may stem from: (1) limited sample size per group; and (2) heterogeneity in baseline characteristics (baseline IgE, baseline TNSS score and etc.) which were not stratified during randomization in this exploratory Phase 2 trial. To address these limitations, the subsequent Phase 3 trial was designed with an increased sample size and incorporated stratification based on prognostic covariates during randomization. Given that 90% and 98.5% patients' free‐IgE level were suppressed to below LLOQ in the 100 and 200 mg LP‐003 groups, respectively, it is not surprising that there is minimal difference in clinical outcomes between these dosing regimens when considering the comparable pharmacodynamic efficacy in IgE suppression. This observation suggests that 100 mg of LP‐003 might be sufficient to improve symptoms in those not well‐controlled SAR subjects. As provided in the Appendix A, LP‐003 showed much higher IgE binding affinity (2.08 pM vs. 1790 pM, 800 folder higher) and more potent FcεRI inhibition compared to Omalizumab, making it possible for LP‐003 to adopt a fixed dosing regimen regardless of baseline total serum IgE. Given that cost‐effectiveness is a crucial factor in evaluating the applicability of a biological agent,^28^ the fixed‐dosage regimen enabled by the high affinity of LP‐003 presents a distinct advantage.

Multiple mutations have been introduced in LP‐003 to enhance the pharmacokinetic profiles, which can strengthen the binding activity of Fc fragment of LP‐003 to FcRn in acidic pH, resulting in reduction of the clearance pathways and prolonged drug exposure.^29^ As illustrated in Appendix B(A), LP‐003 was able to block the binding of IgE to recombinant FcεRI with approximately 20‐fold activity compared to Omalizumab, which is consistent with the structural design of LP‐003. In addition, IgE antibodies that can bind to the IgE/FcεRI complex may lead to the activation of the allergen‐independent FcεRI receptor,^30^ resulting in a severe hypersensitivity reaction. Since the binding assay to the IgE/FcεRI complex showed that LP‐003 did not bind to the IgE/FcεRI complex, as shown in Appendix B(B), its safety was ensured.

The efficacy of omalizumab has been previously investigated in SAR patients in a Japanese randomized, placebo‐controlled, double‐blind, phase 3 trial, in which the difference between omalizumab and placebo was reported to be −1.03. It is hard to make definitive comparisons between Omalizumab and LP‐003 based on currently available data, since the trial settings such as pollen load and background treatment are quite different. Further investigation incorporating Omalizumab as a positive control may provide a clearer answer to this question.

Most frequently reported AE in the LP‐003 study was also commonly observed in other anti‐IgE studies (upper respiratory tract infection, nasopharyngitis and urticaria, etc.).^15^, ^21^, ^31^ The frequency of uric acid increase was 5.8% in LP‐003 group and 5.0% in the placebo group, with no statistically significant difference. No hypersensitivity was observed by far and a more accurate estimate of the hypersensitivity will be obtained as more clinical trials are conducted in the future.

Taken together, our study indicates that LP‐003 is a novel high affinity anti‐IgE monoclonal antibody showing promising bioactivity in treating SAR. The high IgE binding affinity and potent FcεRI inhibiting characteristics are the key to stronger and longer suppression of free‐IgE in healthy subjects and AR patients. The fixed dosing irrespective of baseline IgE level as well as potential superiority make LP‐003 a more potent, possibly cost‐effective, and more convenient treatment option. Future clinical studies are required to further investigate the LP‐003's clinical performance on other IgE mediated diseases.

## AUTHOR CONTRIBUTIONS

Study planning and design (K.G., X.W., N.‐C.‐S.B., H.L.). Data collection (K.G., Y.F., L.L., X.Z., Q.W., H.S., X.W.). Study oversight (H.M., J.Y., C.H., H.L.). Data interpretation and manuscript preparation (S.L., C.H., H.L.).

## CONFLICT OF INTEREST STATEMENT

The authors declare no conflicts of interest.

## CONSENT FOR PUBLICATION

Informed consent was obtained from all subjects involved in the study prior to treatment start.

## Supporting information

Appendix A

Appendix B

Appendix C

Appendix D

## Data Availability

Study data cannot be shared openly to protect study participant privacy.
