# Supplementary material for: LP‐003, a novel high‐affinity anti‐IgE antibody for inadequately controlled seasonal allergic rhinitis: A multicenter, randomized, double‐blind, placebo‐controlled phase 2 clinical trial
Source: Clin Transl Allergy. 2025 Jun 22;15(6):e70074. doi: 10.1002/clt2.70074 (PMC12183111; doi:10.1002/clt2.70074)
Supplement: Supplementary file 1 — Appendix A [file CLT2-15-e70074-s004.docx]

**Appendix A. IgE binding kinetics of LP-003 and Omalizumab using KinExA.**

(A) The binding curve of LP-003. (B) The dissolution constant (Kd)for LP-003. (C) The binding curve of Omalizumab. (D) The dissolution constant (Kd)for Omalizumab.
