# Supplementary material for: LP‐003, a novel high‐affinity anti‐IgE antibody for inadequately controlled seasonal allergic rhinitis: A multicenter, randomized, double‐blind, placebo‐controlled phase 2 clinical trial
Source: Clin Transl Allergy. 2025 Jun 22;15(6):e70074. doi: 10.1002/clt2.70074 (PMC12183111; doi:10.1002/clt2.70074)
Supplement: Supplementary file 2 — Appendix B [file CLT2-15-e70074-s001.docx]

**Appendix B. Inhibition of human IgE binding to FcεRI by anti-IgE antibodies.** (A) The binding curves of human IgE and FcεRI in the presence of either Omalizumab, LP-003, and isotype control. (B) The binding curves of IgE antibodies (Omalizumab, LP-003, isotype control and Le27) to IgE/FcεRI compex. Le 27 is an anti-IgE antibody which binds non-competitively to Cε4 domain and serve as a positive control of binding to IgE/FcεRI compex.
