# Supplementary material for: LP‐003, a novel high‐affinity anti‐IgE antibody for inadequately controlled seasonal allergic rhinitis: A multicenter, randomized, double‐blind, placebo‐controlled phase 2 clinical trial
Source: Clin Transl Allergy. 2025 Jun 22;15(6):e70074. doi: 10.1002/clt2.70074 (PMC12183111; doi:10.1002/clt2.70074)
Supplement: Supplementary file 3 — Appendix C [file CLT2-15-e70074-s002.docx]

**Appendix C. Study Protocol**

Registration Classification: Category 1 of Therapeutic Biological Products

**A Randomized, Double-blind, Placebo-controlled, Phase II Clinical**

**Study to Evaluate the Efficacy, Safety, and Pharmacokinetics of**

**LP-003 Injection in Patients with Moderate-to-Severe Seasonal Allergic Rhinitis with Inadequately Controlled Symptoms under Standard-of-Care**

**Study Protocol**

Protocol No.: P10-LP003-02

Version No.: 1.0

Version Date: 27 February 2023

Clinical Trial No.: CTR20231145

Registration classification: Category 1 of Therapeutic Biological Products

Leading site: Beijing Shijitan Hospital, Capital Medical University

Principal Investigator: Professor Xueyan Wang

Sponsor: LongBio Pharma (Suzhou)

**Confidentiality Statement**

All information contained in this protocol is owned by LongBio Pharma (Suzhou) Co., Ltd. It is provided solely for the review by researchers, ethics committees, regulatory authorities, contract research organizations, and clinical trial site management organizations. Without the written approval of LongBio Pharma (Suzhou) Co., Ltd., this information must not be copied, circulated, or disseminated in any other manner, except when necessary to explain it to participants who may join the trial during the process of obtaining informed consent.

**List of Abbreviations**

| Abbreviation | Description |
| --- | --- |
| ADA | Anti-drug antibody |
| ADAS | Anti-drug antibody analysis set |
| AE | Adverse event |
| ALT | Alanine aminotransferase |
| AR | Allergic rhinitis |
| AST | Aspartate aminotransferase |
| AUC | Area under curve |
| CHO | Chinese hamster ovary |
| CRC | Clinical research coordinator |
| CSU | Chronic spontaneous urticaria |
| CTCAE | Common terminology criteria for adverse events |
| DMP | Data management plan |
| DMR | Data management report |
| DVP | Data verification plan |
| eCRF | Electronic case report form |
| EDC | Electronic data collection |
| FcεRI | High-affinity IgE receptor |
| GCP | Good Clinical Practice |
| HIV | Human immunodeficiency virus |
| ICF | Informed Consent Form |
| ICH | International Conference on Harmonisation |
| IgE | Immunoglobulin E |
| IND | Investigational new drug |
| ITT | Intention-to-treat |
| MAD | Multiple ascending dose |
| MedDRA | Medical dictionary for regulatory activities |
| MRT | Mean residence time |
| MTD | Maximum tolerable dose |
| NCI CTCAE | National Cancer Institute Common terminology criteria for adverse events |
| NOAEL | No observed adverse effect level |
| PAR | Perennial allergic rhinitis |
| PD | Pharmacodynamic |
| PDDS | Pharmacodynamic data set |
| PK | Pharmacokinetic |
| PKCS | Pharmacokinetic concentration set |
| PKPS | Pharmacokinetic concentration parameter set |
| Q4W | Every 4 weeks |
| RQLQ | Rhinoconjunctivitis quality of life questionnaire |
| SAD | Single ascending dose |
| SAE | Serious adverse event |
| SAP | Statistical analysis plan |
| SAR | Statistical analysis report |
| SAS | Statistical Analysis System software |
| SC | Subcutaneous |
| SDV | source data verification |
| SoC | System organ classes |
| SOP | Standard operating procedure |
| SPT | Skin prick test |
| SS | Safety set |
| SUSAR | Suspected unexpected serious adverse reaction |
| t_1/2_ | Elimination half-life |
| T_max_ | Time to peak drug concentration |
| TNSS | Total nose symptom score |
| TOSS | Total ocular symptom score |

**Protocol Synopsis**

| **Title** | A Randomized, Double-blind, Placebo-controlled, Phase II Clinical Study to Evaluate the Efficacy, Safety, and Pharmacokinetics of LP-003 Injection in Moderate-to-Severe Seasonal Allergic Rhinitis Patients with Inadequately Controlled Symptoms under Standard-of-Care |
| --- | --- |
| **Sponsor** | LongBio Pharma (Suzhou) Co., Ltd. |
| **Test Drug** | LP-003 injection |
| **Registration classification** | Category 1 of Therapeutic Biological Products |
| **Study Phase** | Phase Ⅱ |
| **Indication** | Moderate-to-Severe Seasonal Allergic Rhinitis with Inadequately Controlled Symptoms under Standard-of-Care |
| **Leading Site** | Beijing Shijitan Hospital, Capital Medical University, China |
| **Objectives and Endpoints** | \| **Study Objectives** \| **Endpoint Measures** \| \| --- \| --- \| \| **Primary Objective** \| **Primary Endpoint** \| \| To evaluate the preliminary efficacy of different doses of LP-003 injection compared with placebo in moderate to severe seasonal allergic rhinitis patients with inadequately symptoms under standard-of-care. \| - The mean daily total nasal symptom score (TNSS) of LP-003 injection compared to the placebo group during the peak pollen period (PPP).   *Note: The total nasal symptom score is the sum of the scores for four nasal symptoms (nasal itching, nasal congestion, sneezing, and runny nose). Each symptom is rated daily on a severity scale from 0 to 3, where 0 indicates no symptoms, 1 indicates mild symptoms, 2 indicates moderate symptoms, and 3 indicates severe symptoms. Therefore, the maximum total nasal symptom score for one day is 12, and the minimum is 0.* \| \| **Secondary Objectives** \| **Secondary Endpoints** \| \| To evaluate the clinical efficacy of different doses of LP-003 injection in moderate to severe seasonal allergic rhinitis patients with inadequately symptoms under standard-of-care. \| - The mean daily nasal symptom score and rescue medication score (DNSMS) during the peak pollen period (PPP); - The mean daily nasal symptom score and rescue medication score (DNSMS) throughout the entire pollen period (PP); - The mean daily ocular symptom score and rescue medication score (DNOMS) throughout the entire pollen period (PP);   *Note: The total ocular symptom score is the sum of two symptoms (itchy eyes/foreign body sensation/red eyes and tearing) scored according to severity from 0 to 3, where 0 is no symptoms, 1 is mild symptoms, 2 is moderate symptoms, and 3 is severe symptoms. Thus, the maximum daily ocular symptom score is 6 and the minimum is 0.*   - The mean daily rescue medication score during the peak pollen period (PPP); - The mean daily rescue medication score throughout the entire pollen period (PP);   *Note: The rescue medication score records the use of two medications for relieving nasal symptoms (loratadine tablets and montelukast sodium tablets) and one medication for relieving ocular symptoms (emedastine difumarate eye drops). The use of each rescue medication, regardless of dose and frequency, is scored as 1 point.*   - The change in Rhinitis Quality of Life Questionnaire (RQLQ) score from baseline at D1, D29, and D57 during the entire pollen period (PP); - The number of days without nasal symptoms during the entire pollen period (PP).   *Note: Days without nasal symptoms are defined as days when all nasal symptom scores are ≤1.*   - The number of days without rescue medication use during the entire pollen period (PP). - The amount of rescue medication used by subjects during the entire pollen period (PP). \| \| To evaluate the safety of different doses of LP-003 injection in moderate to severe seasonal allergic rhinitis patients with inadequately symptoms under standard-of-care. \| - Adverse events/serious adverse events, vital signs, physical examination, nasal examination, injection site examination, laboratory tests (blood routine, urine routine, blood biochemistry), and 12-lead electrocardiogram. \| \| **Pharmacokinetics Objective** \| **Pharmacokinetic Endpoints** \| \| To evaluate the pharmacokinetic characteristics of different doses of LP-003 injection. \| - For up to 36 subjects, blood samples will be collected before administration on D1 and D29, as well as on D8, D15, D22, D43, D57, D85, and D113, to evaluate the blood concentration and PK parameters of LP-003 after multiple subcutaneous injections. - For other subjects, blood samples will be collected before administration on D1 and D29, as well as on D57 and D113, to evaluate the blood concentration and PK parameters of LP-003 after multiple subcutaneous injections. \| \| **Pharmacodynamics Objective** \| **Pharmacodynamic Endpoints** \| \| To evaluate the pharmacodynamic characteristics of different doses of LP-003 injection. \| - For up to 36 subjects, blood samples will be collected before administration on D1 and D29, as well as on D8, D15, D22, D43, D57, D85, and D113, to evaluate the changes in total serum IgE and free IgE levels compared to baseline at different time points. - For other subjects, blood samples will be collected before administration on D1 and D29, as well as on D57 and D113, to evaluate the changes in total serum IgE and free IgE levels compared to baseline at different time points. \| \| **Immunogenicity Objective** \| **Immunogenicity Endpoints** \| \| To evaluate the Immunogenicity characteristics of different doses of LP-003 injection. \| - Blood samples will be collected from subjects before administration on D1 and D29, as well as on D57, and D113, to evaluate the incidence and titers of anti-drug antibodies (ADA) and neutralizing antibodies (Nab). \| \| **Exploratory objective** \| **Exploratory Endpoints** \| \| To explore the exposure-response relationship after multiple administrations of varying doses of LP-003 injection. \| - If data permits, explore the exposure-response relationship after multiple administrations of varying doses of LP-003 injection. \| |
| **Study design** | This study is a multicenter, randomized, double-blind, placebo-controlled Phase II clinical trial aimed at evaluating the effectiveness, safety, and pharmacokinetic characteristics of LP-003 injection for moderate-to-severe seasonal allergic rhinitis patients with symptoms inadequately controlled by standard-of-care.  The study plans to enroll patients with moderate to severe seasonal allergic rhinitis who have been inadequately controlled by standard-of-care during the past 2 pollen seasons. The number of subjects and the grouping ratio are as follows: a total of 180 subjects, with 120 in the experimental group (80 in the 200 mg dose group and 40 in the 100 mg dose group) and 60 in the control group. Subjects will be randomly assigned to the 200 mg dose group, 100 mg dose group, and control group in a ratio of 4:2:3.  Study Groups   \| **Group** \| **Test Drug** \| **Administration** \| **Number of subjects** \| \| --- \| --- \| --- \| --- \| \| Experimental group 1 \| LP-003 injection (100 mg dose) \| SC，Q4W \| 40 \| \| Experimental group 2 \| LP-003 injection (200 mg dose) \| SC，Q4W \| 80 \| \| Control group \| Placebo \| SC，Q4W \| 60 \| |
| **Study population** | Moderate-to-Severe Seasonal Allergic Rhinitis with Inadequately Controlled Symptoms under Standard-of-Care |
| **Sample Size** | The primary efficacy endpoint of this study is the mean total nasal symptom score (TNSS) during the peak pollen period (PPP). Assuming α = 0.025 (one-sided), power (1-β) = 0.8, the superiority margin is 0, the difference in the mean TNSS during the PPP between the experimental group and the control group (experimental group - control group) is -1, the standard deviation is 2, and the experimental group to control group ratio is 2:1. Using PASS (version 22) software for calculation, the required sample size is 146 subjects (97 in the experimental group and 49 in the control group). Considering an approximate 20% dropout rate, a total of 180 subjects are needed (120 in the experimental group and 60 in the control group). |
| **Study Period** | Each subject will participate in the study for approximately 21 weeks, including a screening period of up to 35 days, an 8-week treatment period, and an 8-week follow-up period. |
| **Study Products** | **Investigational product: LP-003 Injection**  Specification: 100 mg/1.0 mL  Dosage and Administration: Subcutaneous administration, 100 mg or 200 mg per dose, administered once every 4 weeks  Storage Conditions: Store and transport at 2~8℃, protected from light. Do not freeze.  Manufacturer: LongBio Pharma (Suzhou) Co., Ltd.  Supplier: LongBio Pharma (Suzhou) Co., Ltd.  **Control product: LP-003 Injection Placebo**  Specification: 0 mg/1.0 mL  Dosage and Administration: Subcutaneous administration, administered once every 4 weeks  Storage Conditions: Store and transport at 2~8℃, protected from light. Do not freeze.  Manufacturer: LongBio Pharma (Suzhou) Co., Ltd.  Supplier: LongBio Pharma (Suzhou) Co., Ltd. |
| **Study Schedules** | This study includes a screening period, a treatment period, and a follow-up period.  **Screening Period**  All potential participants must provide voluntarily signed informed consent, after which they will enter the screening period, lasting up to 35 days. Participants will undergo a Rhinitis Quality of Life Questionnaire (RQLQ) assessment. The investigator will evaluate the eligibility of the participants based on the inclusion and exclusion criteria. The examinations required for the participants include a physical examination, nasal examination, blood pregnancy test (for women of childbearing age), 12-lead electrocardiogram, vital signs, allergen testing (IgE test results within ≤1 year are acceptable), and laboratory tests. Patients with a history of asthma will also undergo a pulmonary function test. During the screening period, participants must record their nasal symptom scores (TNSS) daily in an electronic diary card. Participants who pass the screening will enter the treatment period.  **Treatment Period**  Participants who pass the screening will enter an 8-week treatment period. The sponsor should predict the local pollen conditions, and participants should receive the first dose 2 weeks before to 1 week after the start of the local pollen season. The pollen start date is defined as the first day of 2 consecutive days with a pollen count ≥1 grain/cm²; the last day is defined as the first day of 3 consecutive days without a pollen count; the pollen peak period is defined as the period between the first and last days with a pollen count ≥30 grains/cm².  On Day 1 (D1), participants will undergo vital signs and laboratory tests (laboratory tests within 7 days are acceptable), check the nasal symptom scores (TNSS) recorded in the electronic diary card during the screening period, and reverify the inclusion and exclusion criteria. Eligible participants will be randomly assigned to two different dose groups and a placebo group, and will receive the corresponding investigational drug according to the study grouping. During the entire pollen season, all participants will be given intranasal glucocorticoid (fluticasone propionate nasal spray, 50 μg/spray, 2 sprays per nostril, once daily) as standard concomitant therapy.  During the treatment period, up to 36 participants in the dense sampling group will have PK and PD blood samples collected before dosing on D1, D8, D15, D22, D29, D43, and D57 visits, and ADA blood samples collected before dosing on D1, D29, and D57 visits. Other participants in the non-dense sampling group will have PK, PD, and ADA blood samples collected before dosing on D1, D29, and D57 visits. If participants do not collect PK and PD blood samples, it will not be reported as a protocol deviation.  All participants must complete the corresponding examinations or tests according to the visit schedule. If participants cannot tolerate rhinitis symptoms during the treatment period, they may use loratadine tablets. If symptoms are still not controlled, they need to return to the hospital for a visit, report to the investigator, and the investigator will decide whether to use montelukast sodium tablets. Participants may choose to use epinastine hydrochloride eye drops to relieve eye symptoms.  During the treatment period, participants should carefully record their nasal symptoms, eye symptoms, and use of rescue medication in the electronic diary card daily. If participants use rescue medication during the treatment period, they should record the TNSS in the electronic diary card before using the rescue medication. During the follow-up visits on D1, D29, and D57, participants will complete the Rhinitis Quality of Life Questionnaire (RQLQ). On D1 and D29, participants will be observed in the hospital for 2 hours after receiving the investigational drug or placebo. During this time, the injection site will be observed, and any local reactions at the injection site will be recorded as adverse events (AEs). Adverse events and concomitant medications/treatments occurring during the trial will be recorded.  **Follow-up Period**  Participants who complete the 8-week treatment period will enter the follow-up period according to the visit schedule. Participants in the dense sampling group will return to the hospital for visits on D85 and D113 to complete PK, PD, and immunogenicity blood sample collection according to the visit schedule. Other participants in the non-dense sampling group will return to the hospital for a visit on D113 to complete PK, PD, and immunogenicity blood sample collection. If participants do not collect PK and PD blood samples, it will not be reported as a protocol deviation. Adverse events and concomitant medications/treatments occurring during the trial will be recorded. |
| **Concomitant Medications/Treatments** | **Allowed Concomitant Medications/Treatments:**  During the entire pollen season, all participants should be given intranasal glucocorticoids (fluticasone propionate nasal spray, 50 μg/spray, 2 sprays per nostril, once daily) as standard concomitant therapy.  Additionally, if the investigator deems that a certain medication is necessary for the participant’s health and is expected not to interfere with the assessment of the investigational drug or interact with it, treatment for complications or adverse events, or symptomatic treatment (including blood products, blood transfusion, infusion, antibiotics, and antidiarrheal drugs) can be administered. Prophylactic treatment medications cannot be used before the first dose, but are allowed after the first dose based on the participant's complications or adverse events. Detailed records should be kept of the name (or treatment method), dosage, frequency, and time of administration of the medications used.  **Rescue Medications/Treatments：**  During the study, if participants find it difficult to tolerate nasal symptoms, they may use loratadine tablets (10 mg/tablet, 1 tablet once daily). If symptoms remain uncontrolled, they should return to the hospital for a visit, and the investigator will decide, based on the participant’s actual situation, whether to use montelukast sodium tablets (10 mg/tablet, 1 tablet once daily). For relieving eye symptoms, participants may choose to use epinastine hydrochloride eye drops (specification: 2.5 mg:5 mL, 1 drop in the affected eye twice daily, which can be increased to four times daily if needed).  Rescue medication should be discontinued once symptoms improve; during the use of rescue medication, continue using the investigational drug and standard concomitant therapy.  **Prohibited Concomitant Medications/Treatments:**   1. Participants are prohibited from using other investigational drugs or investigational devices. 2. The use of similar drugs (e.g., omalizumab) within 6 months before screening is prohibited. 3. The use of systemic glucocorticoids within 4 weeks before the screening period is prohibited. 4. The use of intranasal glucocorticoids, mast cell stabilizers, tricyclic antidepressants, leukotriene receptor antagonists, and antihistamines within 1 week before randomization is prohibited. 5. The use of traditional Chinese medicine for allergic rhinitis within 1 week before randomization is prohibited. 6. The use of allergen immunotherapy within 6 months before the screening period (for those who have not completed immunotherapy) or within 3 years before the screening period (for those who have completed immunotherapy) is prohibited. 7. During the study period, apart from standard concomitant therapy and rescue medication, the use of anticholinergic drugs (oral and intranasal anticholinergics, including ipratropium nasal spray), leukotriene receptor antagonists, antihistamines, mast cell stabilizers, decongestants, nasal saline irrigation, tricyclic antidepressants, anti-allergy herbal medicine, immunosuppressants/immunomodulators, and immunotherapy is prohibited. |
| **Inclusion Criteria** | **Participants who meet all of the following criteria may be included in this clinical trial：**   1. Aged 18-65 years at the time of screening, with no restrictions on gender;. 2. Diagnosed with allergic rhinitis according to the "Chinese Guidelines for the Diagnosis and Treatment of Allergic Rhinitis (2022, revised edition)": a) **Symptoms**: Two or more symptoms including paroxysmal sneezing, clear nasal discharge, nasal itching, and nasal congestion, with symptoms lasting or accumulating for more than 1 hour each day. Eye symptoms may also be present, such as itching, tearing, redness, and burning sensation. b)**Signs:** Common signs include pale and edematous nasal mucosa and watery nasal secretions. c) **Allergen Testing:** Positive for at least one allergen in a skin prick test (SPT) and/or serum specific IgE test, or a positive nasal provocation test (acceptable within 12 months prior to screening). 3. Unsatisfactory symptom control under standard-of-care in two consecutive years in the past(self-reported). During the pollen season, despite using a nasal glucocorticoid recommended by the guidelines or combined with an antihistamine, the total nasal symptom score remains ≥ 6, with nasal congestion ≥ 2. 4. Prior to randomization, participants must have experienced nasal symptoms for ≥ 2 days, or nasal and eye symptoms for ≥ 1 day, and the total nasal symptom score must be ≥ 1. 5. Male participants and their partners or female participants must agree to use one or more non-drug contraceptive methods (such as complete abstinence, intrauterine device, partner sterilization, etc.) during the trial and for 6 months after the trial ends, with no plans to donate sperm or eggs. 6. Agree to participate in this clinical trial and voluntarily sign the informed consent form. |
| **Exclusion Criteria** | **Participants who meet any of the following criteria cannot be included in this clinical trial：**   1. Allergic to the investigational drug or its excipients. 2. Coexisting conditions such as drug-induced rhinitis, vasomotor rhinitis, non-allergic rhinitis with eosinophilia syndrome, acute or chronic rhinosinusitis, dry rhinitis, atrophic rhinitis, severe nasal septum deviation, bronchial asthma, or previous asthma attacks during allergy season that required corticosteroids (mild, exercise-induced asthma not requiring medication or only β-agonist treatment during the study may be included). 3. Patients with perennial allergic rhinitis (seasonal allergic rhinitis complicated with perennial allergic rhinitis and present with seasonal episodes may be included). 4. Any nasal or sinus surgery within one year prior to screening. 5. Presence of glaucoma, cataracts, simple ocular herpes, infectious conjunctivitis, or other eye infections (excluding allergic conjunctivitis). 6. Unresolved and ongoing treatment-requiring local or systemic fungal, bacterial, viral, or parasitic infections, or oral candidiasis within four weeks before screening. 7. Clinically significant conditions (judged by investigators) include but not limited to unstable ischemic heart disease, NYHA Class III/IV left ventricular failure, arrhythmia, uncontrolled hypertension, cerebrovascular disease, neurodegenerative diseases, or other neurological disorders, uncontrolled hypo- or hyperthyroidism, other autoimmune diseases, hypokalemia, high adrenal status; past diagnosis of malignant tumors (except basal cell carcinoma or squamous cell skin cancer); history of myocardial infarction within 12 months before screening. 8. Laboratory Abnormalities at Screening: White blood cell count < 2.5 × 10^9/L; AST or ALT > 2.0 × ULN or total bilirubin > 1.5 × ULN; estimated glomerular filtration rate (eGFR) < 55 mL/min/1.73 m². 9. Treatment with similar experimental drugs (e.g., omalizumab) within six months before screening. 10. Use of systemic corticosteroids within four weeks before screening. 11. Use of intranasal corticosteroids, mast cell stabilizers, tricyclic antidepressants, leukotriene receptor antagonists, antihistamines within one week before randomization. 12. Use of traditional Chinese medicine for allergic rhinitis within seven days before randomization. 13. Use of allergen immunotherapy within six months before screening (for those who have not completed immunotherapy) or within three years before screening (for those who have completed immunotherapy). 14. Inability to discontinue use of anticholinergics (oral and intranasal), leukotriene receptor antagonists, antihistamines, mast cell stabilizers, decongestants, nasal saline rinses, tricyclic antidepressants, anti-allergy herbs, immunosuppressants/immunomodulators, immunotherapy, except for standard concomitant treatment and rescue medication as specified in the protocol. 15. Severe dysfunction of the heart, lungs, liver, or kidneys. 16. Poor compliance, such as poor medication adherence, inability to correctly fill out the diary card, or use of prohibited medications. 17. Comorbid neurological or psychiatric disorders that hinder cooperation or willingness to cooperate; legally defined disabilities (blindness, deafness, muteness, intellectual disability, mental disorders, etc.). 18. Plans to travel outside the local area to non-pollen areas for more than two consecutive days or a total of more than three days during the trial period. 19. Pregnant, breastfeeding, or planning to conceive soon. 20. Participation in another clinical drug trial within the last three months. 21. Any other condition that the investigator believes would make the participant unsuitable for the trial. |
| **Withdrawal criteria** | **Investigator's Decision to Withdraw**  Investigator's decision to withdraw refers to the situation where a subject, already enrolled in the trial, is deemed unsuitable to continue participation by the investigator.   1. Poor compliance by the subject, leading the investigator to determine that it is inappropriate for the subject to continue in the trial. 2. The subject experiences a serious adverse event (SAE), leading the investigator to determine that it is inappropriate for the subject to continue in the trial. 3. Any other situation deemed by the investigator as unsuitable for the subject to continue in the trial.   **Subject's Voluntary Withdrawal from the Trial**  According to the informed consent form, subjects have the right to withdraw from the trial at any time. This includes situations where the subject has not formally withdrawn consent but ceases to accept medication and examinations (also considered as withdrawal or dropout). Efforts should be made to understand and document the reasons for withdrawal, such as perceived poor efficacy, intolerable adverse reactions, inability to continue participating in the trial due to personal reasons, financial constraints, or lost to follow-up without specifying a reason.  Regardless of the reason, medical records of subjects who withdraw from the trial should be retained. |
| **Rejection Criteria** | Before statistical analysis, the principal investigator, sponsor, and statisticians will determine whether individual cases should be rejected. If any of the following situations occur, the three parties will comprehensively assess whether to reject the subject based on the extent of trial completion and reasons for withdrawal, and provide related explanations:   1. The selection of the subject violates the inclusion/exclusion criteria, should not have entered the trial, and affects the efficacy evaluation. 2. During the trial, the subject did not use the investigational drug, or it is impossible to conduct effectiveness and safety evaluations according to the trial protocol, or there is no data for the subject. 3. There are significant deviations or violations of the protocol during the trial, which severely impact the effectiveness and safety of the investigational drug. |
| **Premature Termination Criteria** | Premature termination refers to the complete cessation of a clinical trial before its planned completion according to the protocol. The sponsor has the right to terminate the trial at any time. If the investigator, sponsor, or medical monitor finds that continuing the trial may pose potential harm to the subjects, the clinical trial may be stopped after consultation with all relevant parties.  Reasons for early termination of the trial include, but are not limited to, the following:   1. Severe safety issues are discovered during the trial. 2. The investigational drug is found to have poor or no therapeutic effect, lacking clinical value. 3. Major errors in the clinical trial protocol or serious deviations in its implementation make it difficult to evaluate the drug's effects. 4. The sponsor, the National Medical Products Administration, or the ethics committee orders the termination of the trial for any reason.   If the trial is suspended or terminated early, the investigator must immediately inform the subjects and ensure they receive appropriate treatment and follow-up. The investigator must also promptly notify the sponsor and the hospital's ethics committee, providing a detailed written explanation. Relevant regulatory authorities should also be informed. |
| **Interim Analyses** | This study is an exploratory trial. If necessary, an interim analysis will be conducted at an appropriate time. Considering the characteristics of exploratory trials, the interim analysis will not involve the consumption of type I error. |
| **Statistical Analyses** | **Population for Statistical Analysis:**  Intent-to-Treat (ITT) Set: Includes all randomized subjects according to the ITT principle.  Pharmacokinetic Concentration Set (PKCS): Includes data from subjects who had at least one valid blood drug concentration result after administration of the study drug, used for the analysis of blood drug concentration.  Pharmacokinetic Parameter Set (PKPS): Includes pharmacokinetic parameter data obtained from subjects who received at least one dose of the study drug. This dataset is used for descriptive statistics of the pharmacokinetic parameters of the subjects.  Pharmacodynamic Data Set (PDDS): Includes all randomized subjects who received the study drug and have at least one valid pharmacodynamic indicator data point during the trial.  Safety Set (SS): Includes all randomized subjects who received the study drug and have recorded safety indicators.  Anti-drug Antibody Set (ADAS): Includes subjects who received at least one dose of the study drug and have at least one measurable immunogenicity result. ADAS will be used for immunogenicity analysis.  **Primary Endpoints:**  The mean total score of daily nasal symptom scores during the peak pollen period (PPP) in two groups will be compared using a t-test to assess differences.  **Secondary Endpoints:**  The mean total score of daily nasal symptom scores and rescue medication scores during PPP, and during the entire pollen season (PP), as well as the mean total score of daily eye symptom scores and rescue medication scores during PP, will be compared between the two groups using t-tests.  Changes from baseline in Rhinitis Quality of Life Questionnaire (RQLQ) scores at follow-up times D1, D29, and D57 during the entire pollen season (PP) will be compared between the two groups using a repeated measures mixed effects model.  Mean daily rescue medication scores during PPP, mean daily rescue medication scores during PP, number of days without nasal symptoms during PP, number of days without rescue medication use during PP, and the amount of rescue medication used during PP will be compared between the two groups using rank-sum tests.  **Safety Analysis:**  For each subject experiencing adverse events, categorize the number of subjects for each adverse event (AE) according to the System Organ Class (SOC), Preferred Term (PT) from the Medical Dictionary for Regulatory Activities (MedDRA 25.1 or higher), and adverse event grade (according to CTCAE 5.0 or grading criteria for ocular adverse events). Summarize the number and percentage of subjects experiencing each type of adverse event by treatment group.  Safety assessments: describe continuous variables such as laboratory tests, physical examinations, vital signs, etc., by treatment group with mean ± standard deviation, maximum, minimum, median values, and changes in measurements before and after administration. Describe categorical variables using cross-tabulations to show changes from normal to abnormal before and after administration.  **Pharmacokinetics and Pharmacodynamics:**  Statistically describe the number of cases, mean, standard deviation, coefficient of variation, minimum, median, maximum, and geometric mean of drug concentrations at each time point. Plot concentration-time curves based on sampling time points and average or individual concentrations.  Perform pharmacokinetic analysis based on blood concentration data if feasible.  Statistically describe the number of cases, mean, standard deviation, coefficient of variation, minimum, median, maximum, and geometric mean of pharmacodynamic concentrations at each time point. Plot individual and average efficacy (E)-time (t) curves based on sampling time points.  **Immunogenicity:**  Summarize immunogenicity data at different time points using descriptive statistics and calculate positivity rates.  **Exploratory Endpoints:**  Explore exposure-response relationships after multiple doses of different strengths of LP-003 injection using exploratory analyses. |

**Schedule of Activities for Non-intensive Sampling Group**

| **Visit time**  **Assessment items** | **Screening period** | **Treatment period** | | | | **Follow-up Period** |
| --- | --- | --- | --- | --- | --- | --- |
|  | **V1** | **V2^1^** | **V3** | **V4/Early Withdrawal** | | **V5^13^** |
|  | **D_-35_ ~ D_-1_** | **D_1_** | **D_29_** | **D_57_** | | **D_113_** |
| **Time Window** | **NA** | **±1d** | **±2d** | **±2d** | | **±7d** |
| **General Information** |  |  |  |  | |  |
| Informed Consent Acquisition | X |  |  |  | |  |
| Demographic Data | X |  |  |  | |  |
| Medical History and Medication History | X |  |  |  | |  |
| **Screening/Disqualification Tests** |  |  |  |  | |  |
| Allergen Testing^2^ | X |  |  |  | |  |
| Pulmonary Function Test^3^ | X |  |  |  | |  |
| Vital Signs^4^ | X | X | X | X | |  |
| Physical Examination^5^ | X |  |  | X | |  |
| Nasal Examination^6^ | X |  |  | X | |  |
| Laboratory Tests^7^ | X | X | X | X | |  |
| Pregnancy Test  (Women of Childbearing Potential)^8^ | X | X^7^ |  | X | |  |
| Injection Site Examination^9^ |  | X | X |  | |  |
| 12-lead ECG | X |  |  | X | |  |
| **Efficacy Assessment** |  |  |  |  | |  |
| Nasal Symptom Score^10^ | X | X | | | |  |
| Ocular Symptom Score^10^ |  | X | | | |  |
| Rhinoconjunctivitis Quality of Life Questionnaire  (RQLQ)^11^ | X | X | X | | X |  |
| **Enrollment and Randomization** |  |  |  | |  |  |
| Inclusion/Exclusion Criteria | X | X |  | |  |  |
| Randomization |  | X |  | |  |  |
| **Other Activities** |  |  |  | |  |  |
| PK Blood Sample Collection^12^ |  | X | X | | X | X |
| PD Blood Sample Collection ^12^ |  | X | X | | X | X |
| Immunogenicity Blood Sample Collection ^12^ |  | X | X | | X | X |
| Drug Administration^13^ |  | X | X | |  |  |
| Adverse Events |  | X | | | | |
| Concomitant Medication Use | X | | | | | |

Note:

1. **Sponsorship Requirement:** The sponsor must predict the timing of Visit V2 based on local pollen conditions. The first dose during the treatment period should be administered within 2 weeks before or 1 week after the start of pollen season.
2. **Allergen Testing Results:** If one or more allergen tests are positive before randomization, results from skin prick tests (SPT), intradermal tests, or specific IgE in serum (results from IgE tests within the last year are acceptable) are recognized.
3. **Pulmonary Function Tests:** Only patients with a history of asthma undergo pulmonary function tests.
4. **Vital Signs:** Blood pressure, pulse, and body temperature are measured during screening, prior to D1 dosing, at visits on D29 and D57, and at early withdrawal or end of study.
5. **Physical Examination:** Includes general condition, skin and mucous membranes, head, neck, chest, abdomen, spine and limbs, neurological system, and others.
6. **Nasal Examination:** Conducted during screening and at early withdrawal or end of study. The investigating physician may add additional examination points as deemed necessary. Use of anterior rhinoscopy; further nasal endoscopy may be performed if deemed necessary after anterior rhinoscopy.
7. **Laboratory Tests:** Includes complete blood count, blood biochemistry, urinalysis, and coagulation function; see Appendix 2. Conducted during screening, on D1, D29, D57, and at early withdrawal or end of study. Laboratory tests on D1 may accept results within 7 days.
8. **Blood Pregnancy Test:** Limited to women of childbearing potential (defined as age >54 years with current cessation of menstruation ≥12 months, or those who have undergone hysterectomy, bilateral oophorectomy, or medically confirmed ovarian failure). Conducted during screening and at early withdrawal or end of study; a urine pregnancy test is conducted prior to D1 dosing.
9. **Injection Site Examination:** Following each administration, subjects are observed for 2 hours in the hospital. During this period, the condition of the injection site is observed; any local reactions observed are recorded as adverse events (AE).
10. **Subject Diary Card Use:** Before using the diary card, subjects receive training from on-site staff on how to use the electronic diary. From screening to D57 or early withdrawal, subjects are required to complete the diary card twice daily. The diary card records daily nasal/ocular symptom scores and rescue medication use, including nasal symptoms (sneezing, runny nose, nasal itching, nasal congestion) and ocular symptoms (eye itching/sensation of foreign body/redness, tearing). Additionally, subjects should record nasal symptom scores before using rescue medication. Specifically:
    - Total Nasal Symptom Score (TNSS) is the sum of scores for four symptoms (sneezing, runny nose, nasal itching, nasal congestion) evaluated by the subject. Symptom severity is rated from 0 to 3 (0: no symptoms; 1: mild symptoms, easily tolerated; 2: moderate symptoms, bothersome but tolerable; 3: severe symptoms, intolerable affecting daily life and/or sleep).
    - Total Ocular Symptom Score (TOSS) is the sum of scores for two symptoms (eye itching/sensation of foreign body/redness, tearing) evaluated by the subject. Symptom severity is rated from 0 to 3 (0: no symptoms; 1: mild symptoms, easily tolerated; 2: moderate symptoms, bothersome but tolerable; 3: severe symptoms, intolerable affecting daily life and/or sleep).
    - Usage of two nasal symptom relief medications (levocetirizine tablets and montelukast sodium tablets) and one ocular symptom relief medication (fluorometholone eye drops) should be recorded, including the dose, date, and time of use of rescue medications.
11. **Rhinoconjunctivitis Quality of Life Questionnaire (RQLQ):** Assesses the patient's perception of the quality of life related to allergic rhinitis, including seven domains (activity, sleep, non-nasal/ocular symptoms, practical problems, nasal symptoms, ocular symptoms, and emotions) with 28 items rated on a scale of 0 (no trouble) to 6 (extremely troubled). The overall RQLQ is the average of all scores. Conducted onsite at visits on D1, D29, D57.
12. **PK, PD, Immunogenicity Blood Sample Collection:** Non-confidential group subjects undergo PK, PD, and ADA blood sample collection before D1 dosing, before D29 dosing, and at visits on D57 and D113. If subjects do not have PK or PD blood samples collected, it is not reported as a protocol deviation.
13. **Administration of Study Drugs:** Experimental drugs or comparator drugs are administered separately on D1 and D29. Throughout the pollen season, all subjects receive fluticasone nasal spray as standard adjunctive therapy.

**Schedule of Activities for Intensive Sampling Group**

| **Visit time**  **Assessment items** | **Screening period** | **Treatment Period** | | | | | | | **Follow-up Period** | |
| --- | --- | --- | --- | --- | --- | --- | --- | --- | --- | --- |
|  | **V1** | **V2^1^** | **V3** | **V4** | **V5** | **V6** | **V7** | **V8/Early Withdrawal** | **V9** | **V10** |
|  | **D_-35_ ~ D_-1_** | **D_1_** | **D_8_** | **D_15_** | **D_22_** | **D_29_** | **D_43_** | **D_57_** | **D_85_** | **D_113_** |
| **Time Window** | **NA** | **±1d** | **±1d** | **±2d** | **±2d** | **±2d** | **±2d** | **±2d** | **±7d** | **±7d** |
| **General Information** | | | | | | | | | | |
| Informed Consent Acquisition | X |  |  |  |  |  |  |  |  |  |
| Demographic Data | X |  |  |  |  |  |  |  |  |  |
| Medical History and Medication History | X |  |  |  |  |  |  |  |  |  |
| **Screening/Disqualification Tests** | | | | | | | | | | |
| Allergen Testing^2^ | X |  |  |  |  |  |  |  |  |  |
| Pulmonary Function Test^3^ | X |  |  |  |  |  |  |  |  |  |
| Vital Signs^4^ | X | X |  |  |  | X |  | X |  |  |
| Physical Examination^5^ | X |  |  |  |  |  |  | X |  |  |
| Nasal Examination^6^ | X |  |  |  |  |  |  | X |  |  |
| Laboratory Tests^7^ | X | X |  |  |  | X |  | X |  |  |
| Pregnancy Test  (Women of Childbearing Potential)^8^ | X | X^7^ |  |  |  |  |  | X |  |  |
| Injection Site Examination^9^ |  | X |  |  |  | X |  |  |  |  |
| 12-lead ECG | X |  |  |  |  |  |  | X |  |  |
| **Efficacy Assessment** | | | | | | | | | | |
| Nasal Symptom Score^10^ | X | X | | | | | | |  |  |
| Ocular Symptom Score^10^ |  | X | | | | | | |  |  |
| Rhinoconjunctivitis Quality of Life Questionnaire  (RQLQ)^11^ | X | X |  |  |  | X |  | X |  |  |
| **Enrollment and Randomization** | | | | | | | | | | |
| Inclusion/Exclusion Criteria | X | X |  |  |  |  |  |  |  |  |
| Randomization |  | X |  |  |  |  |  |  |  |  |
| **Other Activities** | | | | | | | | | | |
| PK Blood Sample Collection^12^ |  | X | X | X | X | X | X | X | X | X |
| PD Blood Sample Collection ^12^ |  | X | X | X | X | X | X | X | X | X |
| Immunogenicity Blood Sample Collection ^12^ |  | X |  |  |  | X |  | X |  | X |
| Drug Administration^13^ |  | X |  |  |  | X |  |  |  |  |
| Adverse Events |  | X | | | | | | | | |
| Concomitant Medication Use | X | | | | | | | | | |

Note：

1. The sponsor needs to predict the local pollen conditions to determine the time of visit V2. The first dose during the treatment period should be administered within 2 weeks before the pollen season starts or within 1 week after it begins.
2. Allergen test results: Before randomization, subjects must test positive for one or more allergens. Results from skin prick tests (SPT), intradermal tests, or serum-specific IgE tests are acceptable (IgE test results within one year are acceptable).
3. Pulmonary function test: Only patients with a history of asthma will undergo a pulmonary function test.
4. Vital signs: Blood pressure, pulse, and temperature will be measured during the screening period, before dosing on D1, and during visits on D29, D57, and at withdrawal/early termination.
5. Physical examination: Includes general condition, skin and mucous membranes, head, neck, chest, abdomen, spine and limbs, nervous system, and others.
6. Nasal examination: Conducted during the screening period and at withdrawal/early termination. Additional examination time points may be added as deemed necessary by the study doctor. Anterior rhinoscopy is used, and if necessary, nasal endoscopy may be performed.
7. Laboratory tests: Includes blood routine, blood biochemistry, urinalysis, and coagulation function, as detailed in Appendix 2. Conducted during the screening period, and on visits D1, D29, D57, and at withdrawal/early termination. Laboratory tests on D1 can accept results within 7 days.
8. Pregnancy test: For women of childbearing potential only (women >54 years old who have been amenorrheic for ケ2 months, those who have undergone hysterectomy or bilateral oophorectomy, or those with medically confirmed ovarian failure are considered non-childbearing). Sterilized women do not need a pregnancy test. Conducted during the screening period, at withdrawal/early termination, and a urine pregnancy test before dosing on D1.
9. Injection site examination: After each dose, subjects need to be observed in the hospital for 2 hours. Any local reaction at the injection site should be recorded as an adverse event (AE).
10. Before using the diary card, subjects need to be trained by on-site staff on how to use the electronic diary. From the screening period to D57 or early termination, subjects need to complete the diary card twice daily. The diary records daily nasal/ocular symptom scores and rescue medication use, including nasal symptoms (sneezing, rhinorrhea, nasal itching, nasal congestion) and ocular symptoms (itchy eyes/foreign body sensation/red eyes, tearing). Before using rescue medication, subjects should also perform a nasal symptom score. Specifics:
    - Total Nasal Symptom Score (TNSS) is the sum of the scores for four symptoms (sneezing, rhinorrhea, nasal itching, nasal congestion) evaluated by the subject on a 0-3 scale (0 = no symptoms, 1 = mild symptoms (easily tolerated), 2 = moderate symptoms (annoying but tolerable), 3 = severe symptoms (intolerable, affecting daily life or sleep)).
    - Total Ocular Symptom Score (TOSS) is the sum of the scores for two symptoms (itchy eyes/foreign body sensation/red eyes, tearing) evaluated by the subject on a 0-3 scale (0 = no symptoms, 1 = mild symptoms (easily tolerated), 2 = moderate symptoms (annoying but tolerable), 3 = severe symptoms (intolerable, affecting daily life or sleep)).
    - Record the use of two nasal symptom relief medications (loratadine tablets and montelukast sodium tablets) and one ocular symptom relief medication (epinastine fumarate eye drops). Record the dosage, date, and time of rescue medication use.
11. Rhinoconjunctivitis Quality of Life Questionnaire (RQLQ) assesses the impact of allergic rhinitis on quality of life, including 7 categories (activity, sleep, non-nasal/ocular symptoms, practical problems, nasal symptoms, ocular symptoms, and emotions) with 28 items rated on a 7-point scale (0 = no trouble, 6 = extremely troubled). The overall RQLQ score is the average of all items. RQLQ scoring is conducted on-site during visits on D1, D29, and D57.
12. PK, PD, immunogenicity blood sample collection: For up to 36 subjects in the intensive sampling group, PK and PD blood samples will be collected before dosing on D1, and during visits on D8, D15, D22, before dosing on D29, and during visits on D43, D57, D85, and D113. If a subject does not have PK or PD samples collected, it will not be reported as a protocol deviation. Intensive sampling subjects will have ADA blood samples collected before dosing on D1, and during visits on D29, D57, and D113.
13. Subjects will be administered the study drug or control drug on D1 and D29. Throughout the pollen season, all subjects will receive fluticasone propionate nasal spray as standard concomitant medication.

**1 Introduction**

- 1. **Study background**

Allergic rhinitis (AR), is a non-infectious chronic inflammatory disease of the nasal mucosa primarily mediated by immunoglobulin E (IgE) in atopic individuals following exposure to allergens. According to the type of allergen, it is generally divided into seasonal allergic rhinitis and perennial allergic rhinitis. Epidemiological surveys show that the prevalence of AR has significantly increased in recent years, becoming a major chronic inflammatory respiratory disease that seriously affects patients' quality of life and socio-economic status ^[1]^. Current first-line treatment options for AR include nasal steroids, antihistamines, leukotriene receptor antagonists, etc. Most patients are well controlled, but a small percentage of moderate to severe AR patients have inadequate responses. In recent years, international studies have shown that anti-IgE monoclonal antibodies can be effectively and safely used in the treatment of AR, offering a promising new approach to treating AR ^[2]^.

Anti-IgE antibodies are the most promising class of preparations targeting IgE/FcεRI. Their therapeutic effect is based on their ability to specifically bind and neutralize free IgE in serum, preventing IgE molecules from binding to FcεRI on target cells, thereby inhibiting allergen-induced early/late-phase hypersensitivity reactions. At the same time, they may also block allergen presentation, reducing inflammation caused by Th2 cell activation. In 1987, the first proposal for using anti-IgE antibodies to treat IgE-mediated allergic diseases was made. In 1993, Kolbinger et al. reported the humanization process of a murine monoclonal antibody (MaE11), which is also known as omalizumab or Xolair. The FDA approved omalizumab for asthma treatment in 2003, urticaria treatment in 2014, and allergic rhinitis treatment in 2020. Currently, omalizumab is the only globally approved anti-IgE therapeutic biological agent, approved in Japan for severe AR resistant to both pharmacological and non-pharmacological interventions. Multiple randomized controlled trials have shown that omalizumab treatment for AR can alleviate symptoms, reduce rescue medication use, and improve patient quality of life ^[2, 3-4]^.

LP-003 is a humanized anti-human IgE monoclonal antibody, IgG1-κ, composed of human framework regions and complementarity-determining regions that bind to IgE. It selectively binds to human immunoglobulin E (IgE). Preclinical studies have shown that LP-003 effectively blocks the binding of human IgE to FcεRIα and FcεRII (CD23) receptors in vitro. Its ability to block FcεRIα protein and inhibit activation of RBL-2H3-FcεRIα-NFATLuc cells is significantly stronger than that of the similar drug omalizumab. Pharmacological results in vivo show that 0.5 μg of LP-003 in mice has a greater inhibitory effect on free IgE than 2 μg of omalizumab. In cynomolgus monkeys, different concentrations of LP-003 can inhibit free IgE levels in a dose-dependent manner, with the high-dose group maintaining free IgE levels below the detection limit 28 days after administration. In summary, LP-003 exhibits pharmacological and toxicological similarities to omalizumab, with stronger biological effects.

- 1. **Investigational Product**

The active ingredient in LP-003 injection is a humanized anti-IgE monoclonal antibody, produced by Chinese hamster ovary (CHO) cells in serum-free medium. This protein is composed of two heavy chains, each containing 448 amino acids, and two light chains, each containing 213 amino acids. Each heavy chain contains an N-glycan chain at Asn298, with a molecular weight of 148.2 kDa.

LP-003 selectively binds to human IgE. By specifically binding to a particular region of IgE, it dose-dependently reduces free IgE levels and simultaneously inhibits the binding of IgE to the high-affinity receptor FcεRI on the surface of effector cells (mast cells, basophils). This reduces the activation of inflammatory cells (such as the degranulation of mast cells) and the release of various inflammatory mediators, thereby inhibiting allergen-induced early and late-phase hypersensitivity reactions. It may also block allergen presentation and reduce inflammation caused by Th2 cell activation. The proposed indication is for moderate to severe seasonal allergic rhinitis that is poorly controlled by standard treatment. Therefore, we are conducting this clinical study to investigate the safety and efficacy of LP-003 injection in treating moderate to severe seasonal allergic rhinitis that is inadequately controlled by standard-of-care treatment.

- 1. **Non-clinical Study**

For the non-clinical pharmacological and toxicological evaluation, the following non-clinical studies were completed: target sequence homology comparison across different species, in vitro target binding affinity tests, in vivo pharmacodynamic tests, safety pharmacology, pharmacokinetics (PK), toxicology (including toxicokinetics, immunogenicity, local irritation), in vitro hemolysis, and tissue cross-reactivity tests.

- - 1. **Pharmacological studies**

In *in vitro* tests, the ability of LP-003 and omalizumab to block the binding of human IgE to FcεRIα and FcεRII (CD23) receptors was measured. A rat basophil cell line, RBL-2H3, was transfected with human FcεRIα and NFATLuc reporter genes to establish the RBL-2H3-FcεRIα-NFATLuc cell line. This cell line was used to evaluate the ability of LP-003 and omalizumab to inhibit human IgE or house dust mite allergen-activated human serum in activating cells to release the reporter gene. The results showed that both LP-003 and omalizumab effectively blocked the binding of human IgE to FcεRIα and FcεRII (CD23) receptors and inhibited the expression of the reporter gene. LP-003 demonstrated stronger blocking and cell-binding inhibition capabilities compared to omalizumab, being several times more effective in blocking FcεRIα protein and inhibiting RBL-2H3-FcεRIα-NFATLuc cell activation.

Species cross-reactivity tests and Fc segment function characterization tests showed that LP-003 does not bind to IgE from rats, mice, or dogs but binds to human and cynomolgus monkey IgE with affinities of 2.14 E-10M and 2.94 E-10M, respectively. LP-003 does not induce ADCC, ADCP, or CDC effects and has a higher affinity for human FcRn compared to wild-type IgG1.

*In vivo* pharmacodynamic tests were conducted using four animal models to evaluate the inhibitory effects of LP-003 on IgE and IgE-mediated signaling pathways: a human IgE reconstruction model, inhibition of normal cynomolgus monkey IgE, an immediate-type hypersensitivity model, and an asthma model. Results showed that in the human IgE reconstruction model, 0.5 μg of LP-003 had a much greater inhibitory effect on free IgE than 2 μg of omalizumab. In cynomolgus monkeys, various concentrations of LP-003 inhibited free IgE levels in a dose-dependent manner, with high-dose groups maintaining free IgE levels below the detection limit 28 days post-administration. In the immediate-type hypersensitivity model, all groups of LP-003 effectively inhibited the decrease in mouse body temperature, with 10 μg of LP-003 and 20 μg of omalizumab having comparable effects, while 20 μg of LP-003 maintained normal body temperature. In the asthma model, LP-003 at 3 mg/kg and omalizumab at 6 mg/kg had equivalent inhibitory effects on airway resistance in mice.

In the accompanying toxicology study (cynomolgus monkeys receiving repeated intravenous infusions for 4 weeks, once per week, with a 6-week recovery period), no abnormalities, lesions, or deaths were observed in animals. There were no drug-related changes in blood pressure, ECG, heart rate, respiratory rate, motor function, behavior changes, coordination function, sensory/motor reflexes, or body temperature changes in any dose group before and after administration. These results indicate that LP-003 does not have significant effects on the cardiovascular, respiratory, or central nervous systems.

- - 1. **Pharmacokinetic studies**

Pharmacokinetic studies of LP-003 were conducted in cynomolgus monkeys to evaluate its pharmacokinetic characteristics in vivo, including single-dose and repeated-dose studies. In the single-dose study, 24 cynomolgus monkeys were randomly divided into four groups (3 per gender per group) and administered LP-003 either subcutaneously at doses of 1, 5, and 25 mg/kg, or intravenously at 5 mg/kg. In the repeated-dose study (concurrent with the repeat-dose toxicity test), 30 cynomolgus monkeys were randomly divided into three groups (5 per gender per group) and received intravenous injections of 10, 30, and 100 mg/kg LP-003 once weekly for five weeks.

**Single-dose pharmacokinetic study results:**

After subcutaneous or intravenous administration, no significant gender differences were observed in Cmax and AUC0-t across groups. In the subcutaneous administration groups, the mean Cmax and AUC0-t increased with the dosage, and this increase was proportional to the dosage in the medium and high-dose groups. After intravenous administration, the peak concentration (Cmax) was reached approximately 5 minutes post-administration. After subcutaneous administration, the peak concentrations were reached at approximately 6-72 hours for the 1 mg/kg group, 24-120 hours for the 5 mg/kg group, and 72-168 hours for the 25 mg/kg group.

Due to the detection of anti-drug antibodies (ADA) in 4/6 animals in the intravenous group, 2/6 in the low-dose subcutaneous group, 5/6 in the medium-dose group, and 4/6 in the high-dose group, there was significant individual variation in blood drug concentrations. For ADA-negative animals, the pharmacokinetic parameters over 0-1320 hours were calculated as follows: Cmax was 3 μg/mL, 91.6 μg/mL, 356 μg/mL, and 149 μg/mL for the 1 mg/kg, 5 mg/kg, and 25 mg/kg subcutaneous groups and the 5 mg/kg intravenous group, respectively. The AUC0-1320h values were 209 hμg/mL, 41800 hμg/mL, 195000 hμg/mL, and 18200 hμg/mL, respectively. The half-life (t1/2) values were 81.7 hours, 135 hours, 173 hours, and 140 hours, respectively, and clearance (CL) values were 3.30 mL/h/kg, 0.119 mL/h/kg, 0.145 mL/h/kg, and 0.268 mL/h/kg, respectively.

Considering the minimal impact of ADA within the first week post-administration, pharmacokinetic parameters for 0-144 hours were also calculated. The results for all animals showed Cmax values of 3.65 μg/mL, 68.4 μg/mL, 360 μg/mL, and 147 μg/mL, and AUC0-144h values of 286 hμg/mL, 5770 hμg/mL, 39000 hμg/mL, and 8330 hμg/mL for the 1 mg/kg, 5 mg/kg, and 25 mg/kg subcutaneous groups and the 5 mg/kg intravenous group, respectively. The t1/2 values were 97.1 hours, 12.7 hours, N/A, and 111 hours, respectively, and the clearance values were 3.64 mL/h/kg, 1.6 mL/h/kg, N/A, and 0.483 mL/h/kg. The bioavailability calculated from the AUCs over this period for the 5 mg/kg intravenous and subcutaneous groups was 69.3%.

**Repeated-dose pharmacokinetic study results**:

After administration, no significant gender differences were observed in Cmax and AUC0-t across groups. The mean Cmax and AUC0-t values increased with dosage in the subcutaneous groups, and this increase was proportional to the dosage. The t1/2 values for the subcutaneous 1, 5, and 25 mg/kg groups and the intravenous 5 mg/kg group were 81.7, 135, 173, and 140 hours, respectively. After intravenous administration, the peak concentration (Cmax) was reached approximately 5 minutes post-administration.

The Cmax and AUC0-t values within the first 7 days (0-144 hours) post a single intravenous 5 mg/kg dose were proportional to the toxicokinetic data within the first 7 days following the first administration of repeated intravenous doses of 10, 30, and 100 mg/kg, indicating that LP-003 exhibited linear pharmacokinetic characteristics over the 5 mg/kg to 100 mg/kg dose range with no significant gender differences. With repeated intravenous administration, LP-003 accumulated in the bodies of cynomolgus monkeys (accumulation ratios between 2.8-3.0), with no significant differences in accumulation ratios across dose groups.

**Tables 1, 2, and 3** summarize the pharmacokinetic parameters of LP-003 for single intravenous and subcutaneous doses and repeated intravenous doses, respectively.

**Table 1: Main Pharmacokinetic Parameters (Mean) of LP-003 in Cynomolgus Monkeys After Single Intravenous and Subcutaneous Administration**

| Administration Route | Dose | Animals (n) | Calculated animals (n) | t_1/2_ | T_max_ | C_max_ | AUC_0-t_ | AUC_0-∞_ | Vd | CL | MRT_0-t_ |
| --- | --- | --- | --- | --- | --- | --- | --- | --- | --- | --- | --- |
|  |  |  |  | (h) | (h) | (μg/mL) | (h*μg/mL) | (h*μg/mL) | (mL/kg) | (mL/h/kg) | (h) |
| s.c. | 1 mg//kg | 6^a^ | 4 | 81.7 | 31.5 | 3.00 | 209 | 349 | 495 | 3.30 | 64.8 |
| s.c. | 5 mg//kg | 6^a^ | 1 | 135 | 120 | 91.6 | 41800 | 42000 | 23.2 | 0.119 | 395 |
| s.c. | 25 mg//kg | 6^a^ | 2 | 173 | 156 | 356 | 195000 | 200000 | 34.7 | 0.145 | 372 |
| i.v. | 5 mg//kg | 6^a^ | 2 | 140 | 0.0833 | 149 | 18200 | 18700 | 54.2 | 0.268 | 179 |

a：After administration, ADA (anti-drug antibodies) were detected in 4/6 animals in the intravenous administration group, 2/6 animals in the low-dose subcutaneous group, 5/6 animals in the medium-dose subcutaneous group, and 4/6 animals in the high-dose subcutaneous group. Pharmacokinetic parameters were not calculated for ADA-positive animals. Due to the remaining number of animals (1 in the subcutaneous group and 2 in the intravenous group at 5 mg/kg) being too low, the bioavailability could not be calculated.

**Table 2: Main Pharmacokinetic Parameters (Mean) of LP-003 in Cynomolgus Monkeys After Single Intravenous and Subcutaneous Administration (0-144 Hours)**

| Administration Route | Dose | Sex | Animals (n) | t_1/2_ | T_max_ | C_max_ | AUC_0-t_ | AUC_0-∞_ | Vd | CL | MRT_0-t_ |
| --- | --- | --- | --- | --- | --- | --- | --- | --- | --- | --- | --- |
|  |  |  |  | (h) | (h) | (μg/mL) | (h*μg/mL) | (h*μg/mL) | (mL/kg) | (mL/h/kg) | (h) |
| SC | 1 mg/kg | F | 3 | 46.9 | 18 | 3.26 | 233 | 347 | 254 | 3.52 | 43.9 |
|  |  | M | 3 | 197 | 50 | 4.05 | 340 | 259 | 1100 | 3.86 | 64.6 |
|  |  | Sum | 6 | 97.1 | 34 | 3.65 | 286 | 318 | 537 | 3.64 | 54.3 |
| SC | 5 mg/kg | F | 3 | -^a^ | 120 | 92.6 | 7590 | -^a^ | -^a^ | -^a^ | 93.6 |
|  |  | M | 3 | 12.7 | 88 | 44.1 | 3960 | 3130 | 29.4 | 1.6 | 75.3 |
|  |  | Sum | 6 | 12.7 | 104 | 68.4 | 5770 | 3130 | 29.4 | 1.6 | 84.4 |
| SC | 25 mg/kg | F | 3 | -^a^ | 88 | 380 | 39400 | -^a^ | -^a^ | -^a^ | 72.8 |
|  |  | M | 3 | -^a^ | 128 | 340 | 38500 | -^a^ | -^a^ | -^a^ | 81.9 |
|  |  | Sum | 6 | -^a^ | 108 | 360 | 39000 | -^a^ | -^a^ | -^a^ | 77.3 |
| IV | 5 mg/kg | F | 3 | 87.6 | 0.0833 | 151 | 7300 | 12000 | 48.5 | 0.626 | 46.8 |
|  |  | M | 3 | 145 | 24.1 | 143 | 9360 | 18900 | 54.9 | 0.269 | 65 |
|  |  | Sum | 6 | 111 | 12.1 | 147 | 8330 | 14800 | 51 | 0.483 | 55.9 |

-^a^： Due to the data being collected only for 0-144 hours post-administration, some pharmacokinetic parameters could not be calculated. The bioavailability was calculated to be 69.3% based on the AUC (0-144 hours) for the 5 mg/kg subcutaneous and intravenous groups.

**Table 3: Pharmacokinetic Parameters of Repeat Intravenous Administration of LP-003 in Cynomolgus Monkeys (First Dose) (Mean)**

| Dose | Sex | Animals (n) | t_1/2_ | T_max_ | C_max_ | AUC_0-t_ | AUC_0-∞_ | Vd | CL | MRT_0-t_ |
| --- | --- | --- | --- | --- | --- | --- | --- | --- | --- | --- |
|  |  |  | (h) | (h) | (μg/mL) | (h*μg/mL) | (h*μg/mL) | (mL/kg) | (mL/h/kg) | (h) |
| 10 mg/kg | F | 5 | 104 | 0.0833 | 330 | 24100 | 37600 | 39 | 0.331 | 61.2 |
|  | M | 5 | 127 | 0.0833 | 313 | 20300 | 39200 | 42.5 | 0.48 | 54.6 |
|  | Sum | 10 | 116 | 0.0833 | 321 | 22200 | 38400 | 40.7 | 0.405 | 57.9 |
| 30 mg/kg | F | 5 | 214 | 0.0833 | 845 | 74400 | 181000 | 51 | 0.169 | 72.5 |
|  | M | 5 | 212 | 0.0833 | 1100 | 95600 | 224000 | 40.7 | 0.145 | 71.6 |
|  | Sum | 10 | 213 | 0.0833 | 972 | 85000 | 202000 | 45.8 | 0.157 | 72.1 |
| 100 mg/kg | F | 5 | 212 | 0.0833 | 3520 | 303000 | 715000 | 43.5 | 0.148 | 73 |
|  | M | 5 | 356 | 0.0833 | 3120 | 273000 | 1020000 | 49.8 | 0.132 | 74 |
|  | Sum | 10 | 284 | 0.0833 | 3320 | 288000 | 865000 | 46.7 | 0.14 | 73.5 |

- - 1. **Toxicology Studies**

During the 4-week intravenous infusion toxicity test in cynomolgus monkeys (once weekly, a total of 5 doses), no animal deaths were observed. Clinical observations, body weight, food consumption, clinical pathology (hematology, coagulation, plasma biochemistry), and macroscopic morphology did not reveal any significant toxicity reactions related to LP-003. The maximum tolerated dose (MTD) for intravenous injection of LP-003 in cynomolgus monkeys was greater than 200 mg/kg.

During the 4-week intravenous injection of LP-003, followed by a 6-week recovery period, accompanied by safety pharmacology, toxicokinetics, immunogenicity, and local administration irritation evaluation, no animal deaths were observed. Clinical observations, ophthalmic examinations, body weight, food consumption, body temperature (rectal temperature), clinical pathology (hematology, coagulation, plasma biochemistry, lymphocyte immune phenotyping, immunoglobulins and complement, urine dry chemistry, and urine sediment), and cytokine results (IL-2, IL-4, IL-5, IL-6, TNF-α, IFN-γ) did not show any changes related to LP-003.

Pathological examination (macroscopic observation of organs post-necropsy, organ weight, and histopathological examination) results at the end of the dosing period showed that two male animals in the 100 mg/kg group and one female animal in the 10 mg/kg group had enlarged spleens. Additionally, one male and one female animal in the 100 mg/kg group had multifocal white discoloration of the liver. These changes were not observed at the end of the recovery period and were considered background lesions for cynomolgus monkeys of this age group. Histopathological examination did not reveal any treatment-related changes. No abnormalities were observed at the administration site. The No Observed Adverse Effect Level (NOAEL) was 100 mg/kg. After the fourth dose in the 100 mg/kg group, the mean Cmax for males and females was 6900 µg/mL and 8500 µg/mL, respectively, and the mean AUC0-t for males and females was 772000 hµg/mL and 968000 hµg/mL, respectively. In vitro hemolysis tests showed that LP-003 did not cause hemolysis or aggregation of human red blood cells, indicating no potential hemolytic toxicity. Tissue cross-reactivity tests showed no specific binding of LP-003 to cynomolgus monkey or human tissues.

Separate reproductive toxicity studies have not been conducted. In the 4-week repeat-dose toxicity test, macroscopic observation, weight, and histopathological examination of the reproductive organs (male and female) of cynomolgus monkeys in all dose groups did not reveal any treatment-related changes.

In summary, non-clinical pharmacology and toxicology studies have confirmed that LP-003 can inhibit free IgE or block the binding of IgE to its receptors and downstream signaling pathways, improving allergic symptoms associated with IgE-mediated Type I hypersensitivity reactions. LP-003 demonstrated superior pharmacological effects compared to omalizumab and did not exhibit any unexpected adverse reactions, indicating good safety.

- 1. **Clinical Study**
     1. **Phase I Clinical Study Overview**

The Phase I clinical trial involved a single-dose and multiple-dose escalating study of LP-003 injection in healthy adult volunteers. The purpose was to evaluate the safety, tolerability, and pharmacokinetic characteristics of single and multiple injections of different doses of LP-003 injection in healthy subjects.

The SAD study was a single-center, randomized, double-blind, placebo-controlled, single ascending dose trial design. Five dose groups were set up: 0.3 mg/kg (S1 group, preliminary test group), 1.0 mg/kg (S2 group), 3.0 mg/kg (S3 group), 6.0 mg/kg (S4 group), and 10.0 mg/kg (S5 group). The 0.3 mg/kg group was a preliminary test group, recruiting 2 subjects (administered the test drug without randomization). For the S2-S4 dose groups, 10 subjects were recruited for each group (8 in the test drug group and 2 in the placebo group). The S5 dose group recruited 8 subjects (6 in the test drug group and 2 in the placebo group), with each dose group receiving a single administration.

The MAD study included three dose groups: 2.0 mg/kg, 4.0 mg/kg, and 6.0 mg/kg. Each group recruited 10 subjects (8 in the test drug group and 2 in the placebo group), with administrations every two weeks for a total of four doses.

The SAD trial has completed testing for the five dose groups. The MAD trial is ongoing (as of the time of drafting this protocol). Based on the data collected so far, most adverse events were grade I or mild, with no serious or severe adverse events observed. The results indicate that LP-003 injection is safe and tolerable within the dose range of 0.3 mg/kg to 10 mg/kg. For more details, see the safety section of the Phase I clinical trial.

- - 1. **Summary of Clinical Studies on Similar Products**

**Omalizumab（XOLAIR^®^）**

Omalizumab (XOLAIR®) is a humanized anti-IgE monoclonal antibody drug developed by Novartis. It was first approved in the United States in 2003 for the treatment of moderate to severe persistent asthma in adults and adolescents. Subsequently, it was approved for chronic idiopathic urticaria in 2014 and for the treatment of severe chronic rhinosinusitis with nasal polyps in adults in 2018. Omalizumab was later marketed in the EU and Japan, and in 2017, it was approved in China. Currently, omalizumab is the only approved anti-IgE biological agent worldwide. In Japan, it is approved for severe allergic rhinitis that is unresponsive to drug and non-drug interventions.

In Phase II and Phase III clinical trials for asthma and nasal polyps, omalizumab was administered based on baseline IgE levels and body weight of the subjects, with dosing frequencies of every 2 weeks or every 4 weeks, and treatment cycles of 28 weeks or 24 weeks. For chronic urticaria, Phase II and Phase III trials used fixed dosing regimens of 150 mg or 300 mg every 4 weeks, with treatment cycles of 12 weeks or 24 weeks. Multiple clinical studies have shown that omalizumab has a good overall safety profile, with adverse event rates comparable to the control group. Common adverse reactions include fever, headache, injection site pain, swelling, erythema, and itching, most of which are mild to moderate and transient. Rare allergic reactions such as bronchospasm, hypotension, syncope, urticaria, and/or angioedema of the larynx or tongue have been observed. Most reactions occur within 2 hours after the first and subsequent injections, but some reactions occur after 2 hours, even up to 24 hours post-injection.

A randomized, double-blind, placebo-controlled Phase III clinical trial was conducted in patients with severe AR who had not achieved adequate control of their symptoms despite using standard AR treatments for the past 2 years^[5]^. The study involved 346 subjects who were randomly assigned to the treatment group and the placebo control group. Subjects received omalizumab based on their serum total IgE levels (IU/mL) and body weight (kg) during the screening period, while all subjects continued to receive antihistamines and nasal corticosteroids as standard of care (SoC) treatment. The results showed that the treatment group had a lower mean Nasal Symptom Score (3.65±1.56) during the peak pollen period compared to the control group (4.70±2.18), with a statistically significant difference between the two groups (p<0.001). The treatment group also showed reduced mean rescue medication scores (0.2±0.024) and improved Rhinoconjunctivitis Quality of Life Questionnaire (RQLQ) scores compared to the control group. Safety results were similar between the two treatment groups, with the most commonly reported adverse events (AEs, incidence ≥2%) being nasopharyngitis, pharyngitis, and influenza. No positive anti-omalizumab antibodies were detected in subjects, indicating very low immunogenicity of omalizumab.

Post-marketing reports indicate that the incidence of severe allergic reactions in patients exposed to omalizumab is about 0.2%^[10]^. A history of severe allergic reactions unrelated to omalizumab may be a risk factor for severe allergic reactions after using omalizumab. The observed overall incidence of malignancies in clinical trials of omalizumab was consistent with that reported for the general population, indicating no increased risk of malignancies associated with the drug.

Reproductive studies in cynomolgus monkeys showed that subcutaneous injections of omalizumab up to 75 mg/kg per week during the period of organogenesis (at least 8 times the maximum clinical dose over 4 weeks, based on mg/kg) did not result in maternal toxicity, embryotoxicity, or teratogenicity^[6]^. Additionally, no adverse effects on fetal or neonatal growth were observed when administered during late pregnancy, delivery, and lactation.

**Ligelizumab**

Arm JP^[7]^ investigated the pharmacokinetics, pharmacodynamics, and safety of Ligelizumab in atopic healthy subjects. The SAD (Single Ascending Dose) study involved intravenous infusion with dose ranges from 0.1 to 10 mg/kg. A total of 73 subjects were enrolled, with 60 completing the study. The MAD (Multiple Ascending Dose) study involved subcutaneous injections with dose ranges from 0.2 to 4 mg/kg, administered every two weeks for a total of four doses. A total of 110 subjects were enrolled, with 96 completing the study. The most common adverse events in both SAD and MAD studies were headache and upper respiratory tract infections. Most adverse events, excluding injection site reactions, were mild to moderate in severity and unrelated to the study drug.

In addition, two Phase II clinical trials (NCT02477332 and NCT02649218) were conducted on patients with chronic spontaneous urticaria (CSU). These studies were multicenter, randomized, double-blind, placebo- and active-controlled dose-finding studies in patients with refractory CSU not adequately controlled by H1 antihistamines^[4,8]^. The trials aimed to evaluate the efficacy and safety of monthly subcutaneous injections of Ligelizumab as an add-on therapy. A total of 382 patients were randomized to receive either monthly subcutaneous injections of Ligelizumab (24 mg, 72 mg, 240 mg), 300 mg of Omalizumab, placebo for 20 weeks, or a single dose of 120 mg Ligelizumab. The results showed that by week 12, 30%, 51%, and 42% of patients receiving Ligelizumab 24 mg, 72 mg, and 240 mg, respectively, achieved complete resolution of urticaria, compared to 26% in the Omalizumab group and 0% in the placebo group. The dose-response relationship was confirmed. Additionally, at week 12, 30%, 44%, and 40% of patients receiving Ligelizumab 24 mg, 72 mg, and 240 mg, respectively, achieved complete symptom control, compared to 26% in the Omalizumab group and 0% in the placebo group. The results also indicated that the highest proportion of patients achieving complete control of CSU symptoms was in the 72 mg Ligelizumab group compared to those receiving Omalizumab or placebo. Two Phase III clinical trials (NCT03580369 and NCT03580356) were conducted to further evaluate the efficacy and safety of Ligelizumab (QGE031) compared to placebo and Omalizumab in over 2000 adult and adolescent (≥12 years) patients with chronic spontaneous urticaria inadequately controlled by H1 antihistamines. These studies were multicenter, randomized, double-blind, active- and placebo-controlled, parallel-group studies^[9]^. Patients were randomly assigned to receive 72 mg Ligelizumab, 120 mg Ligelizumab, 300 mg Omalizumab, or placebo every 4 weeks for one year. Patients initially randomized to placebo switched to 120 mg Ligelizumab starting from week 24 until the end of the 52-week treatment period. The primary endpoint was the change in Urticaria Activity Score over 7 days (UAS7) from baseline to week 12. The results of these studies showed that by week 12, Ligelizumab demonstrated superiority over placebo in the primary endpoint but did not show superiority over Omalizumab.

**Other IgE Monoclonal Antibodies under Development**

The main IgE monoclonal antibodies currently under development are FB825, UB-221, XmAb®7195, and DM-101. Among these, only UB-221 and FB825 are in Phase II clinical trials, while the others are in Phase I clinical trials.

The Phase I clinical study of FB825 used intravenous administration with single ascending doses (SAD) ranging from 0.003 to 10 mg/kg. The Phase I clinical study of UB-221 also used intravenous administration with SAD ranging from 0.02 to 10 mg/kg. The Phase II clinical trial of UB-221 is currently ongoing, designed as a double-blind, placebo-controlled study aimed at evaluating the efficacy of UB-221 as a long-term add-on therapy for the treatment of chronic spontaneous urticaria (CSU). In the Phase I clinical study of XmAb®7195, intravenous administration was used for SAD, while subcutaneous administration was used for multiple ascending doses (MAD), with a dosing frequency of once every four weeks. The Phase I clinical study of DM-101 used subcutaneous administration for SAD.

- 1. **Rationale of the study**

LP-003 injection, developed by LongBio (Suzhou) Co., Ltd., is a humanized anti-human IgE monoclonal antibody. This antibody is of the IgG1-κ type, composed of a human framework region and murine antibody complementarity-determining regions that bind to IgE. It selectively binds to human immunoglobulin E (IgE), preventing IgE molecules from binding to FcεRI on target cells, thereby inhibiting allergen-induced early and late phase hypersensitivity reactions. A similar product, Omalizumab (XOLAIR®), is the only approved treatment for severe allergic rhinitis (AR) unresponsive to both drug and non-drug interventions, currently available only in Japan. Numerous randomized controlled trials have demonstrated that Omalizumab can alleviate symptoms, reduce the use of rescue medication, and improve the quality of life in patients with severe AR unresponsive to other treatments, showing significant efficacy.

To date, non-clinical studies of LP-003 injection have shown that it has similarities to Omalizumab in terms of pharmaceutical and pharmacological toxicology, with even stronger biological effects. Phase I clinical trial results in healthy adult volunteers involving single and multiple ascending doses indicate that LP-003 injection is safe and tolerable within the tested dose range. Therefore, LP-003 injection is expected to be significantly effective in treating patients with moderate to severe seasonal allergic rhinitis who respond inadequately to standard treatments.

According to the "Drug Registration Regulation" and supporting documents "Notice on the Classification and Application Requirements of Biological Products Registration" (2020 No. 43) issued by the National Medical Products Administration (NMPA), LP-003 is registered as a Class 1 therapeutic biological product. Based on the drug clinical trial approval notice issued by the NMPA, we plan to conduct a randomized, double-blind, placebo-controlled Phase II clinical study to evaluate the efficacy, safety, and pharmacokinetic characteristics of LP-003, providing support for subsequent clinical trials.

- 1. **Overall risk/benefit assessment**
     1. **Known Potential Risks**

Currently, a Phase I clinical study of LP-003 injection is ongoing, with blinding not yet lifted. It is not yet possible to determine whether adverse events related to the study drug are caused by LP-003 injection. Therefore, based on existing Phase I data, the product is assessed as having no serious risks, as no severe adverse events have occurred in Phase I.

Based on preclinical studies of LP-003 injection and safety data from clinical studies of the same target drugs such as Omalizumab and Ligelizumab, potential risks include:

- **Systemic and local reactions**: Injection site swelling, injection site erythema, injection site pain, injection site itching, fever, flu-like illness, fatigue.
- **Immune system disorders:** Allergic reactions (e.g., urticaria, rash, bronchospasm, wheezing, anaphylactic reactions, angioedema of the throat or tongue), cytokine release syndrome (e.g., fever, hypotension).
- **Gastrointestinal disorders:** Upper abdominal pain, signs and symptoms of indigestion, diarrhea, nausea, vomiting.
- **Nervous system disorders:** Headache, dizziness, somnolence, abnormal sensations.
  - 1. **Known Potential Benefits**

The pharmacological mechanism of LP-003 injection is well-defined in preclinical studies, showing similarities in pharmaceutical and pharmacological toxicology to Omalizumab and demonstrating stronger biological effects. Phase I clinical trial results in healthy adult volunteers, involving single and multiple ascending doses from 0.3 mg/kg to 10 mg/kg, indicate that LP-003 injection is safe and well-tolerated. Additionally, clinical applications of similar marketed products have shown encouraging efficacy in treating severe seasonal allergic rhinitis unresponsive to standard treatments.

Therefore, LP-003 injection is expected to exhibit good safety and provide substantial therapeutic benefits in patients with severe seasonal allergic rhinitis unresponsive to standard treatments.

- - 1. **Assessment of Potential Risks and Benefits**

Currently, first-line treatments for AR in China include nasal steroids, antihistamines, leukotriene receptor antagonists, etc. Most patients achieve good control, but a small subset of moderate to severe AR patients have suboptimal outcomes. In recent years, international studies have shown that anti-IgE monoclonal antibodies can be effective and safe in treating AR, representing a novel approach to AR treatment. Preclinical data indicate that LP-003 injection has good safety. Combined with existing Phase I clinical trial data, which did not report severe adverse events or serious adverse events, and safety data from similar drugs, potential risks have been identified, and corresponding risk control measures have been formulated, making overall risks manageable. According to various literature and reports, clinical applications of similar products after market launch have demonstrated significant therapeutic benefits for moderate to severe seasonal allergic rhinitis patients who are unresponsive to standard treatments.

In conclusion, LP-003 injection is an effective treatment for moderate to severe seasonal allergic rhinitis patients who are unresponsive to standard treatments, showing significant clinical benefits in this population. Compared to similar products, LP-003 injection does not increase safety risks. The results suggest that the benefit-risk ratio of LP-003 injection remains positive, supporting further clinical research. For more potential risks and contingency measures, refer to the Investigator's Brochure and Risk Management Plan.

**2 Study Objectives and Endpoints**

| **Study Objectives** | **Endpoint Measures** |
| --- | --- |
| **Primary Objective** | **Primary Endpoint** |
| To evaluate the preliminary efficacy of different doses of LP-003 injection compared with placebo in moderate to severe seasonal allergic rhinitis patients with inadequately symptoms under standard-of-care. | - The mean daily total nasal symptom score (TNSS) of LP-003 injection compared to the placebo group during the peak pollen period (PPP).   *Note: The total nasal symptom score is the sum of the scores for four nasal symptoms (nasal itching, nasal congestion, sneezing, and runny nose). Each symptom is rated daily on a severity scale from 0 to 3, where 0 indicates no symptoms, 1 indicates mild symptoms, 2 indicates moderate symptoms, and 3 indicates severe symptoms. Therefore, the maximum total nasal symptom score for one day is 12, and the minimum is 0.* |
| **Secondary Objectives** | **Secondary Endpoints** |
| To evaluate the clinical efficacy of different doses of LP-003 injection in moderate to severe seasonal allergic rhinitis patients with inadequately symptoms under standard-of-care. | - The mean daily nasal symptom score and rescue medication score (DNSMS) during the peak pollen period (PPP); - The mean daily nasal symptom score and rescue medication score (DNSMS) throughout the entire pollen period (PP); - The mean daily ocular symptom score and rescue medication score (DNOMS) throughout the entire pollen period (PP);   *Note: The total ocular symptom score is the sum of two symptoms (itchy eyes/foreign body sensation/red eyes and tearing) scored according to severity from 0 to 3, where 0 is no symptoms, 1 is mild symptoms, 2 is moderate symptoms, and 3 is severe symptoms. Thus, the maximum daily ocular symptom score is 6 and the minimum is 0.*   - The mean daily rescue medication score during the peak pollen period (PPP); - The mean daily rescue medication score throughout the entire pollen period (PP);   *Note: The rescue medication score records the use of two medications for relieving nasal symptoms (loratadine tablets and montelukast sodium tablets) and one medication for relieving ocular symptoms (emedastine difumarate eye drops). The use of each rescue medication, regardless of dose and frequency, is scored as 1 point.*   - The change in Rhinitis Quality of Life Questionnaire (RQLQ) score from baseline at D1, D29, and D57 during the entire pollen period (PP); - The number of days without nasal symptoms during the entire pollen period (PP).   *Note: Days without nasal symptoms are defined as days when all nasal symptom scores are ≤1.*   - The number of days without rescue medication use during the entire pollen period (PP). - The amount of rescue medication used by subjects during the entire pollen period (PP). |
| To evaluate the safety of different doses of LP-003 injection in moderate to severe seasonal allergic rhinitis patients with inadequately symptoms under standard-of-care. | - Adverse events/serious adverse events, vital signs, physical examination, nasal examination, injection site examination, laboratory tests (blood routine, urine routine, blood biochemistry), and 12-lead electrocardiogram. |
| **Pharmacokinetics Objective** | **Pharmacokinetic Endpoints** |
| To evaluate the pharmacokinetic characteristics of different doses of LP-003 injection. | - For up to 36 subjects, blood samples will be collected before administration on D1 and D29, as well as on D8, D15, D22, D43, D57, D85, and D113, to evaluate the blood concentration and PK parameters of LP-003 after multiple subcutaneous injections. - For other subjects, blood samples will be collected before administration on D1 and D29, as well as on D57 and D113, to evaluate the blood concentration and PK parameters of LP-003 after multiple subcutaneous injections. |
| **Pharmacodynamics Objective** | **Pharmacodynamic Endpoints** |
| To evaluate the pharmacodynamic characteristics of different doses of LP-003 injection. | - For up to 36 subjects, blood samples will be collected before administration on D1 and D29, as well as on D8, D15, D22, D43, D57, D85, and D113, to evaluate the changes in total serum IgE and free IgE levels compared to baseline at different time points. - For other subjects, blood samples will be collected before administration on D1 and D29, as well as on D57 and D113, to evaluate the changes in total serum IgE and free IgE levels compared to baseline at different time points. |
| **Immunogenicity Objective** | **Immunogenicity Endpoints** |
| To evaluate the Immunogenicity characteristics of different doses of LP-003 injection. | - Blood samples will be collected from subjects before administration on D1 and D29, as well as on D57, and D113, to evaluate the incidence and titers of anti-drug antibodies (ADA) and neutralizing antibodies (Nab). |
| **Exploratory objective** | **Exploratory Endpoints** |
| To explore the exposure-response relationship after multiple administrations of varying doses of LP-003 injection. | - If data permits, explore the exposure-response relationship after multiple administrations of varying doses of LP-003 injection. |

**3 Study Design**

**3.1 Overall design**

This study is a multicenter, randomized, double-blind, placebo-controlled Phase II clinical trial aimed at evaluating the effectiveness, safety, and pharmacokinetic characteristics of LP-003 injection for moderate-to-severe seasonal allergic rhinitis patients with symptoms inadequately controlled by standard-of-care.

The study plans to enroll patients with moderate to severe seasonal allergic rhinitis who have been inadequately controlled by standard-of-care during the past 2 pollen seasons. The number of subjects and the grouping ratio are as follows: a total of 180 subjects, with 120 in the experimental group (80 in the 200 mg dose group and 40 in the 100 mg dose group) and 60 in the control group. Subjects will be randomly assigned to the 200 mg dose group, 100 mg dose group, and control group in a ratio of 4:2:3.

**Table 4. Study Groups**

| **Group** | **Test Drug** | **Administration** | **Number of subjects** |
| --- | --- | --- | --- |
| Experimental group 1 | LP-003 injection (100 mg dose) | SC，Q4W | 40 |
| Experimental group 2 | LP-003 injection (200 mg dose) | SC，Q4W | 80 |
| Control group | Placebo | SC，Q4W | 60 |

**3.1.1 Selection of Dosage**

According to previous research results, LP-003 injection is safe and tolerable within the dose range of 0.3 mg/kg to 10 mg/kg. Given the convenience and compliance associated with using a fixed dose in clinical trials, this study plans to explore the safety and efficacy of administering LP-003 injection at fixed doses. Ultimately, 100 mg per dose (Q4W) and 200 mg per dose (Q4W) will be selected as the study doses.

**3.1.2 Selection of Control Group**

According to the "Allergic Rhinitis: Developing Drug Products for Treatment Guidance for Industry" (September 2018 edition) issued by FDA, general recommendations for the design of phase III trials for allergic rhinitis (SAR and PAR) include conducting double-blind, placebo-controlled, parallel-group studies. This trial intends to include patients with seasonal allergic rhinitis who have had suboptimal responses to previous standard treatments. All subjects will receive conventional standard effective medications as standard concomitant treatments. Additionally, during the trial, subjects will be allowed to use specified rescue medications based on the severity of their symptoms to ensure their safety. Therefore, this trial will use a placebo as the control drug.

**3.1.3 The setting of administration time**

According to the mechanism of action of anti-IgE monoclonal antibodies, they primarily work by specifically binding to IgE, reducing free IgE levels, and inhibiting the binding of IgE to the high-affinity receptor FcεRⅠ. This blocks IgE-mediated hypersensitivity reactions and inflammatory cascades, thereby providing a preventive and blocking effect on the disease. Typically, such drugs (e.g., omalizumab) require some time to start showing efficacy, and anti-IgE monoclonal antibody cannot inhibit IgE already bound to the cell surface FcεRI. Therefore, anti-IgE monoclonal antibody treatment should begin before the pollen season when symptoms are mild or before the inflammatory response occurs, to maximize the drug's efficacy.

This study does not include a lead-in period before administering anti-IgE monoclonal antibody treatment for the following reasons:

1. The purpose of a lead-in period is to exclude subjects whose condition can be effectively controlled with other medications. Given the mechanism of anti-IgE monoclonal antibodies, even if a lead-in period screens out subjects with poor control during the allergy season, an immune response would already have been triggered, and administering the anti-IgE monoclonal antibody at this point would not maximize its efficacy.
2. The pollen season typically reaches its peak within 1-2 weeks. If a one-week lead-in period is set, considering the enrollment time, most subjects would be approaching or past the pollen peak when they start anti-IgE monoclonal antibody treatment, limiting the observed therapeutic effect.
3. For patients with moderate to severe AR who have had suboptimal responses to standard treatment in previous pollen seasons, symptoms are usually mild at the beginning of the pollen season and worsen during the peak. A lead-in period before the treatment phase may affect the enrollment of these patients.

In summary, based on the timing described in the package insert of similar drugs such as omalizumab (XOLAIR®) for administering treatment only during the pollen season, and considering the long half-life of the drug and the mechanism of action of anti-IgE monoclonal antibodies, this study design schedules the first dose to be administered 2 weeks before to 1 week after the expected start of the local pollen season.

Additionally, the study includes patients with a history of suboptimal control on standard treatment based on past medical/treatment history without a lead-in period. Although there may be cases where patients with previously poor control achieve control during the current pollen season, leading to reduced efficacy differences between the treatment and control groups, this conservative efficacy estimate is acceptable in scientific methodology and regulatory review and is commonly used in clinical trials.

**3.2 Randomization**

An independent statistician, unrelated to this study, will use SAS (version 9.4 or higher) software to perform block randomization and generate the subject randomization table (i.e., the subject randomization code file). The number of subjects and grouping ratio are as follows: a total of 180 subjects, with 120 in the experimental group (80 in the 200 mg dose group and 40 in the 100 mg dose group) and 60 in the control group. Subjects will be randomly assigned to the 200 mg dose group, 100 mg dose group, and control group in a ratio of 4:2:3.

During screening, each subject will be identified using a screening number, represented as S+center number+three-digit Arabic number, such as S01001. Subjects who pass the screening will be assigned a randomization number in the order they pass the screening. The randomization number is represented by a three-digit Arabic number, such as 001.

Subjects who withdraw or are withdrawn from the clinical trial for any reason, whether or not they have taken the investigational product, will retain their randomization number and will not be allowed to re-enter the trial.

**3.3 Blinding**

**3.3.1 Blinding Design**

This study adopts a randomized double-blind design, which means that the subjects, researchers, monitors, and data analysts are unaware of the allocation of the treatment. The sponsor or their designated unit will provide the investigational drug and placebo, ensuring that the appearance and weight of the placebo are similar to those of the investigational drug.

**3.3.2 Drug Blinding**

An independent statistician, unrelated to this study, will use the PLAN procedure of SAS (version 9.4 or higher) software to perform block randomization and generate the drug randomization table (i.e., the drug blinding document). The drug number labels will be attached to the corresponding investigational drug or placebo packaging.

The drug blinding process should be documented (see the "Blinding Records" document) and signed by all participants involved in the blinding process.

**3.3.3 Emergency Unblinding**

This trial does not prepare paper emergency letters. If emergency unblinding is necessary, it will be performed in the central randomization system, with the date and reason for unblinding recorded. This information must also be promptly documented in the original medical records and case report forms.

**3.3.4 Blinding Document Preservation**

After completing the drug blinding, the independent statistician will seal the subject blinding document and the drug blinding document, affix the statistical unit's official seal, and the clinical research team leader unit (for verifying the randomization system's output blinding document) and the bio-detection unit (for detection purposes) will each keep a copy.

**3.3.4 Unblinding**

**1) Final Unblinding**

This trial adopts a one-time unblinding method. Once all data have been blindly reviewed and the database is ready to be locked, unblinding will be performed by the research unit and the sponsor. The personnel responsible for maintaining the blinding will provide the grouping information to the statistics department for analysis.

**2) Interim Unblinding**

If one interim analysis is conducted for this project (see Chapter 10.11 for details), it will be carried out by an independent statistician. The blinding information will not be disclosed to personnel other than the non-blinded statistician.

**3) Pharmacovigilance Unblinding**

To quickly report Suspected Unexpected Serious Adverse Reactions (SUSAR) to national drug review agencies, potential SUSAR reports confirmed by medical reviewers can be unblinded for individual subjects by accessing the IWRS. This information will not be disclosed to anyone outside the pharmacovigilance department. Subjects unblinded for pharmacovigilance reasons can continue in the trial if the investigator determines that no further safety issues are at risk; they will not be considered dropouts.

**4) Emergency Unblinding**

Emergency unblinding may be considered under the following circumstances, including but not limited to:

- When the patient experiences a serious adverse reaction;
- When the patient develops a severe complication;
- When symptoms worsen and emergency measures must be taken.

In the event of an emergency requiring rescue measures and it is necessary to know the treatment the subject is receiving, emergency unblinding can be performed in the IWRS system with the consent of the principal investigator. The unblinder's identity, date, and time of unblinding, as well as a clear explanation of the reason for unblinding, must be recorded. The sponsor and lead unit must be notified within 24 hours of the emergency unblinding, and the reason for unblinding must be explained. Subjects undergoing emergency unblinding will be considered dropouts.

**3.4 Trial Duration**

Each subject will participate in this trial for approximately 21 weeks, including a screening period of up to 35 days, an 8-week treatment period, and an 8-week follow-up period.

## Definition of Trial Completion

The clinical trial is considered complete when no further examinations are needed for the subjects or when the last visit of the last subject is finished. Completion refers to either the end of the last visit or the completion of all steps listed in the trial flow chart.

**4 Selection and Withdrawal of Subjects**

**4.1 Study Population**

Moderate-to-severe seasonal allergic rhinitis with inadequately controlled symptoms under standard-of-care.

**4.2 Inclusion Criteria**

Participants who meet all of the following criteria may be included in this clinical trial：

1. Aged 18-65 years at the time of screening, with no restrictions on gender;.
2. Diagnosed with allergic rhinitis according to the "Chinese Guidelines for the Diagnosis and Treatment of Allergic Rhinitis (2022, revised edition)": a) **Symptoms**: Two or more symptoms including paroxysmal sneezing, clear nasal discharge, nasal itching, and nasal congestion, with symptoms lasting or accumulating for more than 1 hour each day. Eye symptoms may also be present, such as itching, tearing, redness, and burning sensation. b)**Signs:** Common signs include pale and edematous nasal mucosa and watery nasal secretions. c) **Allergen Testing:** Positive for at least one allergen in a skin prick test (SPT) and/or serum specific IgE test, or a positive nasal provocation test (acceptable within 12 months prior to screening).
3. Unsatisfactory symptom control under standard-of-care in two consecutive years in the past(self-reported). During the pollen season, despite using a nasal glucocorticoid recommended by the guidelines or combined with an antihistamine, the total nasal symptom score remains ≥ 6, with nasal congestion ≥ 2.
4. Prior to randomization, participants must have experienced nasal symptoms for ≥ 2 days, or nasal and eye symptoms for ≥ 1 day, and the total nasal symptom score must be ≥ 1.
5. Male participants and their partners or female participants must agree to use one or more non-drug contraceptive methods (such as complete abstinence, intrauterine device, partner sterilization, etc.) during the trial and for 6 months after the trial ends, with no plans to donate sperm or eggs.
6. Agree to participate in this clinical trial and voluntarily sign the informed consent form.

**4.3 Exclusion Criteria**

**Participants who meet any of the following criteria cannot be included in this clinical trial：**

1. Allergic to the investigational drug or its excipients.
2. Coexisting conditions such as drug-induced rhinitis, vasomotor rhinitis, non-allergic rhinitis with eosinophilia syndrome, acute or chronic rhinosinusitis, dry rhinitis, atrophic rhinitis, severe nasal septum deviation, bronchial asthma, or previous asthma attacks during allergy season that required corticosteroids (mild, exercise-induced asthma not requiring medication or only β-agonist treatment during the study may be included).
3. Patients with perennial allergic rhinitis (seasonal allergic rhinitis complicated with perennial allergic rhinitis and present with seasonal episodes may be included).
4. Any nasal or sinus surgery within one year prior to screening.
5. Presence of glaucoma, cataracts, simple ocular herpes, infectious conjunctivitis, or other eye infections (excluding allergic conjunctivitis).
6. Unresolved and ongoing treatment-requiring local or systemic fungal, bacterial, viral, or parasitic infections, or oral candidiasis within four weeks before screening.
7. Clinically significant conditions (judged by investigators) include but not limited to unstable ischemic heart disease, NYHA Class III/IV left ventricular failure, arrhythmia, uncontrolled hypertension, cerebrovascular disease, neurodegenerative diseases, or other neurological disorders, uncontrolled hypo- or hyperthyroidism, other autoimmune diseases, hypokalemia, high adrenal status; past diagnosis of malignant tumors (except basal cell carcinoma or squamous cell skin cancer); history of myocardial infarction within 12 months before screening.
8. Laboratory Abnormalities at Screening: White blood cell count < 2.5 × 10^9/L; AST or ALT > 2.0 × ULN or total bilirubin > 1.5 × ULN; estimated glomerular filtration rate (eGFR) < 55 mL/min/1.73 m².
9. Treatment with similar experimental drugs (e.g., omalizumab) within six months before screening.
10. Use of systemic corticosteroids within four weeks before screening.
11. Use of intranasal corticosteroids, mast cell stabilizers, tricyclic antidepressants, leukotriene receptor antagonists, antihistamines within one week before randomization.
12. Use of traditional Chinese medicine for allergic rhinitis within seven days before randomization.
13. Use of allergen immunotherapy within six months before screening (for those who have not completed immunotherapy) or within three years before screening (for those who have completed immunotherapy).
14. Inability to discontinue use of anticholinergics (oral and intranasal), leukotriene receptor antagonists, antihistamines, mast cell stabilizers, decongestants, nasal saline rinses, tricyclic antidepressants, anti-allergy herbs, immunosuppressants/immunomodulators, immunotherapy, except for standard concomitant treatment and rescue medication as specified in the protocol.
15. Severe dysfunction of the heart, lungs, liver, or kidneys.
16. Poor compliance, such as poor medication adherence, inability to correctly fill out the diary card, or use of prohibited medications.
17. Comorbid neurological or psychiatric disorders that hinder cooperation or willingness to cooperate; legally defined disabilities (blindness, deafness, muteness, intellectual disability, mental disorders, etc.).
18. Plans to travel outside the local area to non-pollen areas for more than two consecutive days or a total of more than three days during the trial period.
19. Pregnant, breastfeeding, or planning to conceive soon.
20. Participation in another clinical drug trial within the last three months.
21. Any other condition that the investigator believes would make the participant unsuitable for the trial.

**4.4 Withdrawal criteria**

**4.4.1 Investigator's Decision to Withdraw**

Investigator's decision to withdraw refers to the situation where a subject, already enrolled in the trial, is deemed unsuitable to continue participation by the investigator.

1. Poor compliance by the subject, leading the investigator to determine that it is inappropriate for the subject to continue in the trial.
2. The subject experiences a serious adverse event (SAE), leading the investigator to determine that it is inappropriate for the subject to continue in the trial.
3. Any other situation deemed by the investigator as unsuitable for the subject to continue in the trial.

**4.4.2** **Subject's Voluntary Withdrawal from the Trial**

According to the informed consent form, subjects have the right to withdraw from the trial at any time. This includes situations where the subject has not formally withdrawn consent but ceases to accept medication and examinations (also considered as withdrawal or dropout). Efforts should be made to understand and document the reasons for withdrawal, such as perceived poor efficacy, intolerable adverse reactions, inability to continue participating in the trial due to personal reasons, financial constraints, or lost to follow-up without specifying a reason.

Regardless of the reason, medical records of subjects who withdraw from the trial should be retained.

**4.5 Rejection Criteria**

Before statistical analysis, the principal investigator, sponsor, and statisticians will determine whether individual cases should be rejected. If any of the following situations occur, the three parties will comprehensively assess whether to reject the subject based on the extent of trial completion and reasons for withdrawal, and provide related explanations:

1. The selection of the subject violates the inclusion/exclusion criteria, should not have entered the trial, and affects the efficacy evaluation.
2. During the trial, the subject did not use the investigational drug, or it is impossible to conduct effectiveness and safety evaluations according to the trial protocol, or there is no data for the subject.
3. There are significant deviations or violations of the protocol during the trial, which severely impact the effectiveness and safety of the investigational drug.

**4.6 Screening failure**

All subjects who signed the informed consent form but did not enter randomization will be considered as screening failures. The information of screening failure subjects needs to be recorded in the eCRF, and the investigator should retain, but is not limited to, the following original documents:

1. Signed informed consent form;
2. Demographic information of the subject;
3. Reason for screening failure.

**4.7 Premature Termination Criteria**

Premature termination refers to the complete cessation of a clinical trial before its planned completion according to the protocol. The sponsor has the right to terminate the trial at any time. If the investigator, sponsor, or medical monitor finds that continuing the trial may pose potential harm to the subjects, the clinical trial may be stopped after consultation with all relevant parties. Reasons for early termination of the trial include, but are not limited to, the following:

1. Severe safety issues are discovered during the trial.
2. The investigational drug is found to have poor or no therapeutic effect, lacking clinical value.
3. Major errors in the clinical trial protocol or serious deviations in its implementation make it difficult to evaluate the drug's effects.
4. The sponsor, the National Medical Products Administration, or the ethics committee orders the termination of the trial for any reason.

If the trial is suspended or terminated early, the investigator must immediately inform the subjects and ensure they receive appropriate treatment and follow-up. The investigator must also promptly notify the sponsor and the hospital's ethics committee, providing a detailed written explanation. Relevant regulatory authorities should also be informed.

**5 Treatment**

**5.1 Basic Information on Investigational Drugs**

**Experimental group:**

LP-003 injection

Specification: 100 mg/1.0 mL

Dosage and Administration: Subcutaneous administration, 100 mg or 200 mg per dose, administered once every 4 weeks

Batch number: XXX

Validity: 24 months from the date of production

Storage Conditions: Store and transport at 2~8℃, protected from light. Do not freeze.

Sponsor: LongBio Pharma (Suzhou) Co., Ltd.

**Placebo control group:**

LP-003 injection placebo

Specification: 0 mg/1.0mL

Dosage and Administration: Subcutaneous administration, once every 4 weeks

Batch number: XXX

Validity: 24 months from the date of production

Storage Conditions: Store and transport at 2~8℃, protected from light. Do not freeze.

Sponsor: LongBio Pharma (Suzhou) Co., Ltd.

*Note: The investigational drug is provided free of charge by the sponsor. The sample batch number "XXX" in the drug information indicates that the batch number has not yet been determined. The actual drug packaging label will specify the batch number used in practice, and the protocol will not be updated with batch number information.*

**5.2 Administration Methods**

Subcutaneous administration of LP-003 injection (or LP-003 injection placebo) preparation process is detailed in the drug usage SOP.

LP-003 injection should be removed from the refrigerator and used within 10 minutes to 2 hours at room temperature. Multiple subcutaneous injections at different sites on the subject are allowed (if the injection volume exceeds 2 mL). Recommended injection sites include the outer upper arm, front and middle of the thigh, and abdomen (avoiding the area within 5 cm around the navel), with a minimum interval of 2.5 cm between injections.

LP-003 injection (or LP-003 injection placebo) will be administered via multiple subcutaneous injections at different sites on the subject. Specific details are as follows:

**Experimental group 1 (LP-003 injection, 100 mg/dose):** Each subject will receive 1 vial of LP-003 injection (100 mg/1.0 mL) and 1 vial of LP-003 injection placebo (0 mg/1.0 mL) per dose, administered subcutaneously (SC), once every 4 weeks (Q4W), for a total of 2 doses.

**Experimental group 2 (LP-003 injection, 200 mg/dose):** Each subject will receive 2 vials of LP-003 injection (100 mg/1.0 mL each) per dose, administered subcutaneously (SC), once every 4 weeks (Q4W), for a total of 2 doses.

**Control group:** Each subject will receive 2 vials of LP-003 injection placebo (0 mg/1.0 mL each) per dose, administered subcutaneously (SC), once every 4 weeks (Q4W), for a total of 2 doses.

For detailed administration methods, refer to the drug usage SOP.

**5.3 Drug Management**

**5.3.1 Drug Packaging and Labeling**

All investigational drugs will be packaged and labeled by LongBio Pharma (Suzhou) Co., Ltd. in accordance with Good Clinical Practice (GCP) guidelines and applicable national regulations.

The drug labels will have a standardized format and may include the following information: protocol number, drug number, drug name, specification, usage and dosage, package quantity (for external packaging boxes only), batch number, expiration date, storage conditions, supplier, and a note stating "For Clinical Trial Use Only."

**5.3.2 Drug Supply and Storage**

The investigational drugs will be shipped to the clinical trial sites by a professional transportation company designated by the sponsor, according to the specified transportation conditions. The drugs will be received and properly handled and stored by designated personnel (typically the trial drug management personnel) at the clinical trial sites. The drugs must be stored in a secure location accessible only to the trial drug management personnel. Upon receipt, all investigational drugs must be stored according to the instructions on the drug labels.

**5.3.3 Drug Distribution, Record Keeping, and Return**

The investigational drugs used in this trial will be provided free of charge by the sponsor and distributed to the clinical trial sites as planned. The clinical trial sites will assign dedicated personnel to be responsible for the receipt, storage, distribution, collection, and corresponding records of the investigational drugs.

The investigational drugs will be centrally stored, managed, and distributed by the designated personnel at the clinical trial sites. The responsible personnel at the clinical trial sites will provide written confirmation upon receipt of the investigational drugs and will use the drugs as per the protocol requirements. The responsible personnel will maintain records of the receipt, distribution, and return of the investigational drugs according to the standard operating procedures of the clinical trial sites.

Researchers or their designated personnel must agree not to provide the investigational drugs to any non-enrolled subjects, or any physicians or scientists not authorized for this trial.

Upon completion or termination of the trial, unless authorized by the sponsor to destroy the drugs, all unused or partially used investigational drugs must be returned to the sponsor.

**5.4 Medication Compliance**

During the study period, the medication usage for the investigational treatment will be recorded in the eCRF. Any deviations from the prescribed medication regimen will also be documented in the eCRF, including the dates and reasons for the deviations.

Medication compliance is defined as the actual dose of the drug taken by the subject during the study period divided by the prescribed dose, then multiplied by 100%. It is calculated using the following formula:

Medication Compliance (%) = (Actual Dose Taken / Prescribed Dose) × 100%

**5.5 Concomitant Medications/Treatments**

**5.5.1 Allowed Concomitant Medications/Treatments**

During the entire pollen season, all participants should be given intranasal glucocorticoids (fluticasone propionate nasal spray, 50 μg/spray, 2 sprays per nostril, once daily) as standard concomitant therapy.

Additionally, if the investigator deems that a certain medication is necessary for the participant’s health and is expected not to interfere with the assessment of the investigational drug or interact with it, treatment for complications or adverse events, or symptomatic treatment (including blood products, blood transfusion, infusion, antibiotics, and antidiarrheal drugs) can be administered. Prophylactic treatment medications cannot be used before the first dose, but are allowed after the first dose based on the participant's complications or adverse events. The name (or treatment method), dosage, frequency, and time of administration of the medications used should be detailed in the eCRF.

**Rescue Medications/Treatments：**

During the study, if participants find it difficult to tolerate nasal symptoms, they may use loratadine tablets (10 mg/tablet, 1 tablet once daily). If symptoms remain uncontrolled, they should return to the hospital for a visit, and the investigator will decide, based on the participant’s actual situation, whether to use montelukast sodium tablets (10 mg/tablet, 1 tablet once daily). For relieving eye symptoms, participants may choose to use epinastine hydrochloride eye drops (specification: 2.5 mg:5 mL, 1 drop in the affected eye twice daily, which can be increased to four times daily if needed).

Rescue medication should be discontinued once symptoms improve; during the use of rescue medication, continue using the investigational drug and standard concomitant therapy.

**5.5.2 Prohibited Concomitant Medications/Treatments**

During the trial, subjects are not allowed to use the following medications:

- Subjects are prohibited from using other investigational drugs or devices.
- The use of drugs similar to the investigational drug (e.g., omalizumab) is prohibited within 6 months before screening.
- Systemic corticosteroids are prohibited within 4 weeks before the screening period.
- The use of intranasal corticosteroids, mast cell membrane stabilizers, tricyclic antidepressants, leukotriene receptor antagonists, and antihistamines is prohibited within 1 week before randomization.
- Traditional Chinese medicine treatments for allergic rhinitis are prohibited within 1 week before randomization.
- Allergen immunotherapy is prohibited within 6 months before the screening period for those who have not completed immunotherapy, or within 3 years before the screening period for those who have completed immunotherapy.
- During the study, except for standard concomitant medications and rescue medications, the use of anticholinergic drugs (oral and intranasal anticholinergic drugs, including ipratropium nasal spray), leukotriene receptor antagonists, antihistamines, mast cell membrane stabilizers, decongestants, nasal saline rinses, tricyclic antidepressants, anti-allergic Chinese herbs, immunosuppressants/immunomodulators, and immunotherapy is prohibited.

**5.6 Post-Trial Treatment**

Subjects will not continue to receive the investigational drug treatment after the trial ends. Upon completion or early termination of the trial, all subjects can receive appropriate treatment as prescribed by the investigator.

If necessary, the investigator must provide follow-up medical care for subjects who withdraw early from the trial or refer them to suitable medical care facilities.

**5.7 Treatment of Overdose of Investigational Drug**

An overdose is defined as the administration of a dose of the investigational drug higher than the protocol-specified dose, resulting in clinical signs and symptoms.

If an overdose occurs, the investigator should use their judgment to decide whether additional treatment is necessary.

**6 Trial Procedures and Visit Schedule**

**6.1 Trial Procedures**

This trial consists of the screening period, treatment period/early termination visits, and follow-up period. The visit schedule and procedures to be conducted at each visit should follow the trial flow chart in this protocol. All procedures and evaluations conducted during visits should be recorded in the source documents and eCRF.

During the study, unscheduled visits can be arranged if necessary, based on the subject's request or the investigator's decision. The investigator should inform subjects to contact the investigator immediately if serious adverse events occur. If adverse events (AEs) are suspected, subjects may return to the center for necessary examinations or for AE follow-up. Subjects must ensure effective contraception during the trial and for 6 months after the last administration of the investigational drug (including their partners). During this period, they should use one or more contraceptive methods during sexual intercourse. Details of the contraceptive methods are provided in Appendix 1.

**First Phase: Screening Period (D-35~D-1)**

The screening period lasts up to 35 days. Subjects enter the trial after signing the informed consent form. Demographic information, general questions, medical history, and medication history are collected, and inclusion/exclusion criteria are determined. Subjects must complete a Rhinitis Quality of Life Questionnaire (RQLQ) score once. Researchers evaluate subjects' eligibility based on the inclusion/exclusion criteria, and subjects undergo the following tests: physical examination, nasal examination, blood pregnancy test (for women of childbearing age), 12-lead ECG, vital signs, allergen testing (IgE test results within ≤1 year are acceptable), and laboratory tests. Patients with a history of asthma also need to undergo a lung function test. During the screening period, subjects must record their nasal symptom scores (TNSS) daily in an electronic diary card. Subjects who meet the screening criteria will enter the treatment period.

**Second Phase: Treatment Period (D1~D57)**

The treatment period lasts for 57 days. Subjects who meet the screening criteria will enter an 8-week treatment period. Based on local pollen forecasts, the sponsor should ensure that the first dose is administered between 2 weeks before the start of the pollen season and within 1 week after the start of the pollen season. The start of the pollen season is defined as the first day of two consecutive days with pollen counts ≥1 grain/cm²; the last day is defined as the first of three consecutive days without pollen counts; the peak pollen period is defined as the dates between the first and last day when pollen counts are ≥30 grains/cm².

On D1, subjects undergo vital signs and laboratory tests (laboratory tests within 7 days are acceptable) and review the nasal symptom scores (TNSS) recorded in the electronic diary card during the screening period. Eligibility criteria are verified again, and subjects who meet the criteria are randomly assigned to two different dosage groups and a placebo group. They will be administered the corresponding medication based on the study group. During the entire pollen season, all subjects should be given intranasal corticosteroids (fluticasone propionate nasal spray, 50 μg/spray, two sprays per nostril, once daily) as standard adjunctive treatment.

During the treatment period, up to 36 subjects in the intensive sampling group will have PK and PD blood samples collected on D1 before dosing, D8, D15, D22, D29 before dosing, D43, and D57 visits, and ADA blood samples collected on D1 before dosing, D29 before dosing, and D57 visits. Other non-intensive sampling group subjects will have PK, PD, and ADA blood samples collected on D1 before dosing, D29 before dosing, and D57 visits. If any subject does not have PK or PD blood samples collected, it will not be reported as a protocol deviation.

All subjects should complete the corresponding tests or assessments according to the visit schedule. During the treatment period, if subjects cannot tolerate rhinitis symptoms, they may use loratadine tablets; if symptoms remain uncontrolled, they should return to the center for a visit, report to the researcher, and the researcher will decide whether montelukast sodium tablets can be used. Subjects may use emedastine difumarate eye drops to relieve ocular symptoms.

During the treatment period, subjects should carefully record their nasal symptoms, eye symptoms, and rescue medication use daily in the electronic diary card. If subjects use rescue medication during the treatment period, they should record the nasal symptom scores (TNSS) in the electronic diary card before using the rescue medication. During the D1, D29, and D57 follow-up visits, subjects should complete the Rhinitis Quality of Life Questionnaire (RQLQ) scoring. On D1 and D29, after receiving the investigational drug or placebo, subjects should be observed in the hospital for 2 hours. The injection site should be monitored, and any local reactions should be recorded as adverse events (AEs). All adverse events and concomitant medications/treatments occurring during the trial should be recorded.

**Third Phase: Follow-up Period (D58~D113)**

The follow-up period lasts for 57 days. Subjects who completed the 8-week treatment period will enter the post-treatment follow-up period according to the visit schedule. Subjects in the intensive sampling group should return for visits on D85 and D113 for PK, PD, and immunogenicity blood sample collection according to the visit plan. Other non-intensive sampling group subjects should return for a visit on D113 for PK, PD, and immunogenicity blood sample collection. If any subject does not have PK or PD blood samples collected, it will not be reported as a protocol deviation. All adverse events and concomitant medications/treatments occurring during the trial should be recorded.

The visit schedule and assessments are detailed in the "Intensive Sampling Group and Non-Intensive Sampling Group Trial Flow Chart." If a subject withdraws or discontinues the trial early, relevant evaluations should be conducted as much as possible according to the trial requirements.

**6.2 Visit Schedule for Non-Intensive Sampling Group**

**6.2.1 Screening Period**

**Visit 1: The following items need to be completed during the screening period:**

- Signing the informed consent form
- Collecting demographic information
- Collecting medical history and medication history
- Allergen testing
- Lung function test
- Nasal examination
- Vital signs
- Physical examination
- Pregnancy test (for women of childbearing age)
- 12-lead ECG
- Laboratory tests (complete blood count, urinalysis, blood biochemistry, coagulation function)
- Nasal symptom scores (TNSS)
- Rhinitis Quality of Life Questionnaire (RQLQ)
- Concomitant medications
- Confirmation of inclusion/exclusion criteria

***Note:*** *1) allergen testing can accept IgE test results within the last year; 2) only patients with a history of asthma need to undergo the lung function test.*

**6.2.2** **Treatment Period**

**Visit 2: D1 (First administration day counted as D1)**

- Confirmation of enrollment and randomization
- Administration of medication
- Vital signs
- Urine pregnancy test
- Laboratory tests (complete blood count, blood biochemistry, urinalysis, coagulation function)
- Injection site examination (within 2.0 hours post-administration)
- Rhinitis Quality of Life Questionnaire (RQLQ)
- PK, PD, and immunogenicity blood sample collection
- Adverse events
- Concomitant medications

***Note:*** *Laboratory tests (complete blood count, blood biochemistry, urinalysis, coagulation function) can accept results from within the past 7 days.*

**Visit 3: Day 29 (D29 ± 2 days)**

- Administration of medication
- Vital signs
- Laboratory tests (complete blood count, blood biochemistry, urinalysis, coagulation function)
- Injection site examination (within 2.0 hours post-administration)
- Rhinitis Quality of Life Questionnaire (RQLQ)
- PK, PD, and immunogenicity blood sample collection
- Adverse events
- Concomitant medications

**Visit 4: Day 57 (D57 ± 2 days)**

- Nasal examination
- Vital signs
- Physical examination
- Urine pregnancy test (for women of childbearing potential)
- 12-lead electrocardiogram
- Laboratory tests (complete blood count, urinalysis, blood biochemistry, coagulation function)
- Rhinitis Quality of Life Questionnaire (RQLQ)
- PK, PD, and immunogenicity blood sample collection
- Adverse events
- Concomitant medications

**6.2.3 Follow-up Period**

**Visit 5: Day 113 (D113 ± 7 days)**

- PK, PD, and immunogenicity blood sample collection
- Adverse events
- Concomitant medications

All adverse events occurring during the trial must be monitored and recorded. If necessary, appropriate treatment and management should be provided, and all concomitant medications should be recorded. All adverse events must be followed up until recovery, alleviation, stabilization, or loss to follow-up.

**6.2.4 Early Withdrawal**

Steps to be completed for an early withdrawal visit include:

- Nasal examination
- Vital signs
- Physical examination
- Pregnancy test (for women of childbearing potential)
- 12-lead electrocardiogram
- Laboratory tests (complete blood count, urinalysis, blood biochemistry, coagulation function)
- Nasal symptom score (TNSS)
- Ocular symptom score (TOSS)
- Rhinitis Quality of Life Questionnaire (RQLQ)
- PK, PD, and immunogenicity blood sample collection
- Adverse events
- Concomitant medications

**6.3 Intensive Sampling Group Visit Schedule**

**6.3.1 Screening Period**

**Visit 1: The following visit items need to be completed during the screening period:**

- Signing the informed consent form
- Collecting demographic information
- Gathering medical history and medication history
- Allergen testing
- Pulmonary function test
- Nasal examination
- Vital signs
- Physical examination
- Pregnancy test (for women of childbearing potential)
- 12-lead electrocardiogram
- Laboratory tests (complete blood count, urinalysis, blood biochemistry, coagulation function)
- Nasal symptom score (TNSS)
- Rhinitis Quality of Life Questionnaire (RQLQ)
- Concomitant medications
- Confirmation of inclusion/exclusion criteria

***Notes:*** *1) allergen testing can accept IgE test results within the past year; 2) pulmonary function tests are only required for patients with a history of asthma.*

**6.3.2 Treatment Period**

**Visit 2: D1 (First administration day counted as D1)**

- Confirmation of inclusion/exclusion criteria, randomization
- Administration of medication
- Vital signs
- Urine pregnancy test
- Laboratory tests (complete blood count, blood biochemistry, urinalysis, coagulation function)
- Injection site inspection (within 2 hours post-dose)
- Rhinitis Quality of Life Questionnaire (RQLQ)
- Pharmacokinetics (PK), Pharmacodynamics (PD), and immunogenicity blood sample collection
- Adverse events
- Concomitant medications

***Notes:*** *Laboratory tests (complete blood count, blood biochemistry, urinalysis, coagulation function) can accept results within 7 days.*

**Visit 3: Day 8 (D8 ± 1 day)**

- Pharmacokinetics (PK) and Pharmacodynamics (PD) blood sample collection
- Adverse events
- Concomitant medications

**Visit 4: Day 15 (D15 ± 2 days)**

- Pharmacokinetics (PK) and Pharmacodynamics (PD) blood sample collection
- Adverse events
- Concomitant medications

**Visit 5: Day 22 (D22 ± 2 days)**

- Pharmacokinetics (PK) and Pharmacodynamics (PD) blood sample collection
- Adverse events
- Concomitant medications

**Visit 6: Day 29 (D29 ± 2 days)**

- Administration of medication
- Vital signs
- Laboratory tests (complete blood count, blood biochemistry, urinalysis, coagulation function)
- Injection site inspection (within 2 hours post-dose)
- Rhinitis Quality of Life Questionnaire (RQLQ)
- Pharmacokinetics (PK), Pharmacodynamics (PD), and immunogenicity blood sample collection
- Adverse events
- Concomitant medications

**Visit 7: Day 43 (D43 ± 2 days)**

- Pharmacokinetics (PK) and Pharmacodynamics (PD) blood sample collection
- Adverse events
- Concomitant medications

**Visit 8: Day 57 (D57 ± 2 days)**

- Nasal examinations
- Vital signs
- Physical examinations
- Urine pregnancy test (for women of childbearing potential)
- Laboratory tests (complete blood count, blood biochemistry, urinalysis, coagulation function)
- Injection site inspection (within 2 hours post-dose)
- Rhinitis Quality of Life Questionnaire (RQLQ)
- Pharmacokinetics (PK), Pharmacodynamics (PD), and immunogenicity blood sample collection
- Adverse events
- Concomitant medications

**6.3.3 Follow-up Period**

**Visit 9: Day 85 (D85 ± 7 days)**

- Pharmacokinetics (PK) and Pharmacodynamics (PD) blood sample collection
- Adverse events
- Concomitant medications

**Visit 10: Day 113 (D113 ± 7 days)**

- Pharmacokinetics (PK) and Pharmacodynamics (PD) blood sample collection
- Adverse events
- Concomitant medications

All adverse events occurring during the trial must be monitored and recorded. If necessary, appropriate treatment and management should be provided, and all concomitant medications should be recorded. All adverse events must be followed up until recovery, alleviation, stabilization, or loss to follow-up.

**6.3.4 Early Withdrawal**

Steps to be completed for an early withdrawal visit include:

- Nasal examination
- Vital signs
- Physical examination
- Pregnancy test (for women of childbearing potential)
- 12-lead electrocardiogram
- Laboratory tests (complete blood count, urinalysis, blood biochemistry, coagulation function)
- Nasal symptom score (TNSS)
- Ocular symptom score (TOSS)
- Rhinitis Quality of Life Questionnaire (RQLQ)
- PK, PD, and immunogenicity blood sample collection
- Adverse events
- Concomitant medications

**7 Efficacy and Safety Assessment**

The visit arrangements during the trial are detailed in the Flow Chart for Intensive and Non-intensive Sampling Groups.

**7.1 Efficacy Endpoint Assessment**

**7.1.1 Total Nasal Symptom Score (TNSS)**

From Screening to Day 57 or until early withdrawal, subjects are required to record their Total Nasal Symptom Score (TNSS) twice daily (morning and evening) in a diary card. Additionally, subjects should also perform a TNSS assessment before using rescue medication. The TNSS comprises the sum of scores for four nasal symptoms (nasal itching, nasal congestion, sneezing, rhinorrhea), each rated on a severity scale of 0 to 3: 0 indicates no symptom, 1 indicates mild symptoms, 2 indicates moderate symptoms, and 3 indicates severe symptoms. Thus, the maximum TNSS score per day is 12, and the minimum is 0.

**Table 5. Total Nasal Symptom Score (TNSS)**

| **Symptom/Score** | **0** | **1** | **2** | **3** |
| --- | --- | --- | --- | --- |
| nasal itching | no symptom | mild symptoms, easy to tolerate | obvious symptoms, troublesome, still tolerable | intolerable symptoms affecting daily life and/or sleep |
| nasal congestion | no symptom | mild symptoms, easy to tolerate | obvious symptoms, troublesome, still tolerable | intolerable symptoms affecting daily life and/or sleep |
| sneezing | no symptom | mild symptoms, easy to tolerate | obvious symptoms, troublesome, still tolerable | intolerable symptoms affecting daily life and/or sleep |
| rhinorrhea | no symptom | mild symptoms, easy to tolerate | obvious symptoms, troublesome, still tolerable | intolerable symptoms affecting daily life and/or sleep |

**7.1.2 Total Nasal Symptom Score (TOSS)**

From D1 to D57 or until early withdrawal, subjects are required to record eye symptom scores (TOSS) twice daily (morning and evening) on diary cards. The total score for eye symptoms is the sum of scores for two symptoms (itchiness/sensation of foreign body/redness and tearing). Each symptom is rated on a severity scale from 0 to 3, where 0 indicates no symptoms, 1 indicates mild symptoms, 2 indicates moderate symptoms, and 3 indicates severe symptoms. Thus, the maximum daily total score for eye symptoms is 6, and the minimum is 0.

**Table 6. Total Ocular Symptom Score (TOSS)**

| **Symptom/Score** | **0** | **1** | **2** | **3** |
| --- | --- | --- | --- | --- |
| itchiness/sensation of foreign body/redness | no symptom | mild symptoms, easy to tolerate | obvious symptoms, troublesome, still tolerable | intolerable symptoms affecting daily life and/or sleep |
| tearing | no symptom | mild symptoms, easy to tolerate | obvious symptoms, troublesome, still tolerable | intolerable symptoms affecting daily life and/or sleep |

**7.1.3 Rescue Medication Treatment Score**

From D1 to D57 or until early withdrawal, subjects are required to record the use of rescue medications on diary cards. This primarily includes recording the use of two nasal symptom relievers (loratadine tablets and montelukast sodium tablets) and one eye symptom reliever (emestine fumarate eye drops). The rescue medication score is calculated based on the use of each type of rescue medication, regardless of dosage or frequency, each scored as 1 point.

**Table 7. Rescue Medication Treatment Score**

| Rescue Medication | Score (each day) | |
| --- | --- | --- |
| loratadine tablets and | 1 | |
| montelukast sodium tablets | 1 |  |
| Emestine fumarate eye drops | 1 | |

**7.1.4 Rhinoconjunctivitis Quality of Life Questionnaire (RQLQ) Assessment**

During the screening period, and at visits on D1, D29, and D57, subjects are required to undergo on-site RQLQ scoring. The Rhinoconjunctivitis Quality of Life Questionnaire (RQLQ) assesses the impact of allergic rhinitis on patients' quality of life, covering seven domains (activity limitations, sleep problems, non-nasal/eye symptoms, practical problems, nasal symptoms, eye symptoms, and emotional impact) with a total of 28 items rated on a 7-point scale (0 = not troubled at all, 6 = extremely troubled). Detailed content is available in Appendix 3.

**7.2 Safety Assessment**

**7.2.1 Vital Signs**

This includes temperature, pulse rate, and blood pressure. The investigator may adjust specific assessments based on the patient's condition. Blood pressure and pulse are measured after at least 5 minutes of rest.

**7.2.2 Physical Examination**

A complete physical examination is conducted during the screening period, at the end of the study or upon early withdrawal.

Physical examination includes general condition, skin and mucous membranes, head, neck, chest, abdomen, spine and limbs, neurological system, and others.

After administration, attention should be paid to any discomfort in the subject's skin, and observation of injection site reactions: observe for symptoms such as induration, itching, erythema, pain, swelling, etc. If any abnormalities occur, it is recommended to promptly conduct relevant specialized examinations.

Newly occurring abnormalities judged by the investigator to have clinical significance or abnormalities significantly worsened compared to baseline should be recorded as adverse events.

**7.2.3 Nasal Examination**

Nasal examination is conducted during the screening period, at the end of the study or upon early withdrawal, to determine the presence of nasal mucosal erosion, nasal septal ulcers or perforations, or other nasal cavity diseases that the investigator determines may affect the deposition of intranasal medications, such as acute or chronic sinusitis, drug-induced rhinitis, nasal polyps, or nasal septum abnormalities.

**7.2.4 Laboratory Tests**

Laboratory tests (including blood routine, blood biochemistry, urine routine, and coagulation function) will be conducted according to the visit schedule.

Any laboratory parameter abnormality that is clinically relevant and requires action regarding study treatment (e.g., the appearance of clinical symptoms or signs, or requiring therapeutic intervention), regardless of whether it is specifically required by the study protocol, should be recorded on the adverse event page of the electronic data capture (EDC) system. Laboratory data will be summarized using the Common Terminology Criteria for Adverse Events (NCI CTCAE v5.0). Other analyses will be conducted at the investigator's discretion. Refer to Appendix 2 for specific laboratory test details.

**7.2.5 12-Lead ECG**

ECG examinations should be performed after the subject has rested for at least 5 minutes. If ECG abnormalities are found, the investigator and/or authorized research personnel may decide to retest based on the clinical condition of the subject and evaluate the test results. Unscheduled visits for ECG examinations can also be arranged if clinically indicated.

**7.2.6 Adverse Events**

See "8 Adverse Events and Serious Adverse Events" for details.

**7.3 PK Sample Collection**

It is planned to collect blood samples from up to 36 subjects before dosing on Day 1, Day 8, Day 15, Day 22, Day 29, Day 43, Day 57, Day 85, and Day 113. For other subjects, blood samples will be collected before dosing on Day 1 and Day 29, Day 57, and Day 113. If a subject does not have PK blood samples collected, it will not be reported as a protocol deviation. Blood samples should be collected using tubes containing coagulant, with at least 4 mL of blood collected each time. The separated serum from the collected blood samples will be used for PK testing and backup. The blood concentration and PK parameters of LP-003 after multiple subcutaneous injections will be evaluated.

**7.4 PD Sample Collection**

It is planned to collect blood samples from up to 36 subjects before dosing on Day 1, Day 8, Day 15, Day 22, Day 29, Day 43, Day 57, Day 85, and Day 113. For other subjects, blood samples will be collected before dosing on Day 1 and Day 29, Day 57, and Day 113. If a subject does not have PD blood samples collected, it will not be reported as a protocol deviation. Blood samples should be collected using tubes containing coagulant, with at least 3.5 mL of blood collected each time. The separated serum from the collected blood samples will be used for PD testing and backup. Changes in the total serum IgE and free IgE levels of subjects at different testing time points compared to baseline will be evaluated. If data permits, PD parameters will be calculated.

**7.5 Immunogenicity Testing**

It is planned to collect blood samples from subjects before dosing on Day 1, Day 29, Day 57, and Day 113. Blood samples should be collected using tubes containing coagulant, with at least 4 mL of blood collected each time. The separated serum from the collected blood samples will be used for ADA testing and backup. The incidence of anti-drug antibodies (ADA) and neutralizing antibodies (Nab), as well as antibody titers, will be evaluated.

**7.6 Exploratory Endpoints**

If data permits, exploratory analysis of the exposure-response relationship after multiple doses of different concentrations of LP-003 injection will be conducted.

**8 Adverse Events and Serious Adverse Events**

**8.1 Definition of Adverse Events**

An **adverse event (AE)** is defined as any untoward medical occurrence in a subject who has received the investigational product. This can manifest as symptoms, signs, diseases, or laboratory abnormalities, but does not necessarily have a causal relationship with the investigational product.

Therefore, an adverse event can be any unfavorable or unintended sign (including abnormal laboratory results), symptom, or disease temporally associated with the use of the investigational product, whether or not it is related to the investigational treatment.

AEs do not include the following situations:

- Conditions or laboratory abnormalities that existed or were detected before screening and did not worsen further.
- Situations where no adverse medical event occurred (e.g., hospitalization for elective surgery, social reasons, and/or convenience of the subject).
- Any medical condition or clinically significant laboratory abnormality occurring after the consent form is signed but before the first dose of the investigational treatment, which is unrelated to the study treatment, is not considered an AE. Such situations are considered pre-existing and should be recorded as medical history.

**8.2 Definition of Adverse Drug Reactions**

An adverse drug reaction is any harmful or unintended reaction that may be related to the investigational product occurring in a clinical trial. There must be at least a reasonable possibility of a causal relationship between the investigational product and the adverse event, meaning the connection cannot be ruled out.

**8.3 Acquisition of Adverse Event Information**

Investigators should report all adverse events directly observed or spontaneously reported by subjects in concise language. Additionally, after the start of the trial, subjects should be regularly asked about adverse events as specified in the protocol.

**8.4 Recording Adverse Events**

From the definition of an adverse event, only those adverse medical occurrences that happen after the use of the investigational product are considered "adverse events." However, for the purpose of collecting "safety information" in clinical trials, all adverse medical occurrences that happen after the subject signs the Informed Consent Form (ICF) should be collected and recorded until the end of the follow-up period. Regardless of severity or relationship to the investigational product, they should be recorded in the original documents and the appropriate pages of the Case Report Form (CRF).

Medical conditions occurring from the time the subject signs the ICF to before receiving the investigational product should be considered baseline and recorded as medical history/concomitant diseases in the electronic CRF, not as adverse events, unless they meet one of the following conditions:

- Any injury/damage caused by clinical laboratory procedures;
- Adverse events caused by withdrawal related to the study protocol;
- Adverse events caused by medications other than the investigational product used as part of the treatment protocol.

All adverse events occurring from the time of dosing until the end of the follow-up period should be recorded in the original documents and the CRF. During the trial, all adverse events should be truthfully recorded in the original documents and the CRF, including clinical manifestation characteristics, severity, onset time, end time, measures taken, and outcomes, and their relationship to the investigational product should be determined. Any adverse event occurring during the trial should be recorded in the designated adverse event section of the CRF. The investigator should ensure complete recording of any adverse events occurring during the trial.

If possible, adverse events should be recorded as a specific diagnosis. If a specific diagnosis cannot be recorded, individual abnormal signs, symptoms, or test results should be recorded separately. If a subject's symptoms, signs, or test results existing during the screening period do not worsen after receiving the investigational product, they should not be reported as adverse events. If they worsen after receiving the investigational product, they should be recorded as adverse events. If the same adverse event occurs more than once in a subject, and the subject recovers between events, each occurrence should be recorded separately. If the severity of an adverse event changes during its occurrence, it should be recorded separately.

The outcomes of adverse events can be described as follows:

- Recovered/Resolved: The "end date of (serious) adverse event" should be noted.
- Recovering/Resolving: The event has not completely resolved, but the subject is in the recovery stage. Follow-up is needed.
- Not Recovered/Not Resolved: The event is ongoing.
- Recovered/Resolved with Sequelae: Only applicable if the subject has long-term or lifelong sequelae, such as blindness caused by diabetes or hemiplegia caused by a stroke. The "end date of (serious) adverse event" should be noted.
- Fatal: The "end date of (serious) adverse event" should be noted. The time of death should be recorded if the adverse event was fatal.
- Unknown: The investigator is unable to determine the outcome of the adverse event, such as if the subject is lost to follow-up.

If the outcome of the adverse event is assessed as "recovering/resolving," "not recovered/not resolved," or "unknown," the end date of the adverse event can be temporarily omitted. If the outcome is assessed as "recovered/resolved" or "recovered/resolved with sequelae," the end date of the adverse event must be recorded.

After the subject completes the clinical study, the investigator should follow up on the outcome of adverse events that may be related to the investigational product until they reach a stable state.

**8.5 Criteria for Determining the Severity of Adverse Events**

The severity of adverse events is determined based on the NCI CTCAE 5.0 standards. If an unlisted adverse event occurs, the criteria in Table 8-1 can be referred to:

**Table 8 Criteria for Determining Adverse Events**

| **Grade of Adverse Event** | **Description of Severity** |
| --- | --- |
| Grade 1 | Mild; asymptomatic or mild symptoms; only clinical or diagnostic observations; no intervention needed. |
| Grade 2 | Moderate; minimal, local, or noninvasive intervention required; limiting age-appropriate instrumental activities of daily living (IADL); IADL refers to preparing meals, shopping for clothes, using the telephone, managing finances, etc. |
| Grade 3 | Severe or medically significant but not immediately life-threatening; hospitalization or prolongation of hospitalization indicated; disabling; limiting self-care activities of daily living (ADL); ADL refers to bathing, dressing, feeding, toileting, taking medication, etc., and does not include being bedridden. |
| Grade 4 | Life-threatening consequences; urgent intervention indicated. |
| Grade 5 | Death related to the adverse event. |

**8.6 Criteria for Determining the Relationship Between Adverse Events and the Investigational Product**

The investigator should comprehensively analyze the specific circumstances of the AE, the subject's medical history, concomitant diseases, and concomitant medications to determine the causal relationship between the AE and the investigational product. The investigator should conduct a causal analysis of the symptoms that occur during the medication process to assess the possible association between the AE and the investigational product.

According to the criteria for determining the causal relationship between the investigational product and the adverse event, the relationship can be classified into five levels: definitely related, probably related, possibly related, probably unrelated, and definitely unrelated. The categories of definitely related, probably related, and possibly related are considered adverse drug reactions.

1. **Definitely Related:** The event is consistent with the known type of reaction to the suspected drug, occurs in a reasonable time sequence after drug administration, diminishes or disappears after dose reduction or discontinuation, and reoccurs after re-administration.
2. **Probably Related:** The event is consistent with the known type of reaction to the suspected drug, occurs in a reasonable time sequence after drug administration, diminishes or disappears after dose reduction or discontinuation, but the subject's clinical status or other causes could also explain the event.
3. **Possibly Related:** The event is consistent with the known type of reaction to the suspected drug, occurs in a reasonable time sequence after drug administration, diminishes slightly or not at all after dose reduction or discontinuation, but the subject's clinical status or other causes could also explain the event.
4. **Probably Unrelated:** The event is not consistent with the known type of reaction to the suspected drug, does not occur in a reasonable time sequence after drug administration, and the subject's clinical status or other causes are more likely to produce the event.
5. **Definitely Unrelated:** The event is not consistent with the known type of reaction to the suspected drug, does not occur in a reasonable time sequence after drug administration, the subject's clinical status or other causes can explain the reaction, and the event diminishes or disappears after ruling out clinical symptoms or other causes.

## Follow-up of Adverse Events

All unresolved adverse events at the end of the trial or upon early withdrawal of the subject must be followed up until one of the following situations occurs, which means observation may still be required after the final visit as per the protocol:

1. The event resolves;
2. The event stabilizes;
3. The event returns to baseline level;
4. The event can be attributed to medications other than the investigational product or is unrelated to the trial activities;
5. No further information can be obtained (e.g., the subject refuses to provide more information, or the subject is lost to follow-up despite the researcher's best efforts).

**8.8 Actions Related to the Investigational Product**

- **Permanent Discontinuation**: The investigational product is permanently discontinued due to a specific AE.
- **Dose Unchanged**: The specific AE does not require discontinuation of the investigational product.
- **Dose Reduction**: The dose of the investigational product is reduced due to an AE.
- **Unknown**: This category is used when the action taken is uncertain.
- **Not Applicable**: The investigational product is discontinued for reasons other than a specific AE, such as study termination, subject death, or discontinuation of the investigational product prior to the occurrence of the AE.
- **Temporary Interruption**: The investigational product is temporarily interrupted (paused) due to a specific AE (including subject-initiated interruption) and is resumed thereafter.

**8.9 Definition of Serious Adverse Events**

A **serious adverse event (SAE)** is defined as an adverse medical event that occurs after a subject receives the investigational product and results in death, is life-threatening, causes permanent or significant disability or loss of function, requires hospitalization or prolongs existing hospitalization, or results in a congenital anomaly or birth defect.

- Results in death;
- Life-threatening:

*Note: The definition of "life-threatening" in SAE refers to the subject being at immediate risk of death at the time of the adverse event, not to an event that could hypothetically become life-threatening if more severe.*

- Requires hospitalization or prolongs existing hospitalization:

*Note: The reason for this condition must be due to the adverse event and not due to elective surgery, non-medical reasons, etc. Complications occurring during hospitalization are considered AEs. If complications prolong the current hospitalization or meet other SAE criteria, the event should be considered an SAE.*

- Permanent or significant disability or loss of function:

*Note: The term "disability" means that the outcome of the adverse event significantly interferes with or disrupts the subject's normal life and activities.*

**Congenital anomaly or birth defect**;

**Other important medical events**:

Medical and scientific judgment must be used to decide whether other situations warrant expedited reporting. Important medical events that may not immediately result in death, be life-threatening, or require hospitalization but necessitate medical intervention to prevent one of these outcomes are usually considered serious. For example, important treatments in the emergency room or at home for allergic bronchospasm, cachexia or convulsions without hospitalization, drug dependence or abuse.

The terms "severe" and "serious" are not synonymous. "Severe" is often used to describe the intensity (severity) of a specific event (the intensity of an AE as per NCI CTCAE V5.0, see section 8.5 for details). Each AE's severity and seriousness should be independently assessed and documented in the eCRF.

If an event does not qualify as an AE based on the above definition, it cannot be considered an SAE, even if it meets serious criteria (e.g., hospitalization due to signs/symptoms of the disease under study).

**8.10 Handling Subjects in the Event of an SAE**

If a subject experiences a Serious Adverse Event (SAE), and the researcher determines that it is inappropriate for the subject to continue participating in the trial, the subject should be withdrawn from the trial and given active treatment.

**8.11 Recording and Reporting SAEs**

If an SAE occurs during the trial, the researcher must immediately report all SAEs in writing to the sponsor, except for those serious adverse events that the trial protocol or other documents (such as the investigator’s brochure) specify do not require immediate reporting. Subsequently, a detailed written follow-up report should be provided in a timely manner. The researcher must report the SAE to the sponsor's designated project manager immediately upon becoming aware of it. The researcher should complete the "Serious Adverse Event Report Form" as comprehensively as possible, documenting the name, severity, onset time, duration, measures taken, and outcome of the serious adverse event, and sign and date the form.

**Reporting Pathway for Serious Adverse Events:**

| **Unit** | **Contact** | **Contact Information** |
| --- | --- | --- |
| LongBio Pharma (Suzhou) Co., Ltd. | Hongzhou Yang | Tel: 13564951337  Email: yanghz@longbio.com |

* Upon receiving safety information related to the clinical trial provided by the sponsor, the researcher should promptly acknowledge receipt and review it, consider adjusting the subject's treatment accordingly, communicate with the subject as necessary, and report any suspected unexpected serious adverse reactions to the ethics committee.

If the researcher cannot promptly become aware of the SAE (e.g., if the subject first seeks treatment at an external hospital), they should report it immediately upon becoming aware and document the time they first learned of the SAE.

For all SAEs, the researcher is responsible for following up and providing information to the sponsor within the reporting timelines specified above. Additionally, the sponsor may require the researcher to quickly collect specific supplementary information, which may be more detailed than that recorded on the SAE report form. Typically, this information should include a detailed description of the SAE to enable a complete medical evaluation and an initial independent judgment of the possible cause. In addition, other potential causes such as information on concomitant medications and concomitant diseases must be provided. For reports involving death, the researcher should provide additional necessary materials to the ethics committee and the sponsor or their designated representative, such as autopsy reports and final medical reports. SAE reports and follow-up reports should include the subject's identification code in the clinical trial, not the subject's real name, citizen identification number, address, or other identifying information.

**8.12 Definition of Suspected Unexpected Serious Adverse Reaction**

A Suspected Unexpected Serious Adverse Reaction (SUSAR) refers to a serious adverse reaction that is both suspected and unexpected, where the nature and severity of the clinical manifestation exceed the information available in the Investigator’s Brochure, package insert of a marketed drug, or summary of product characteristics.

**8.13 Reporting of Suspected Unexpected Serious Adverse Reactions**

According to the "Good Clinical Practice for Drugs" (2020 version), the sponsor must immediately analyze and evaluate any safety-related information from any source, including severity, relevance to the investigational drug, and whether it is an expected event. The sponsor must promptly report the SUSAR to all participating investigators and clinical trial institutions, ethics committees, and report the SUSAR to the drug regulatory authorities and health authorities.

Upon receiving safety information related to the clinical trial provided by the sponsor, the investigator should promptly acknowledge receipt and review it, consider adjusting the subject's treatment accordingly, communicate with the subject as necessary, and report any SUSAR provided by the sponsor to the ethics committee. The content of individual safety reports for unexpected serious adverse reactions should be reported according to the relevant requirements of ICH "E2B(R3): Clinical Safety Data Management: Data Elements for Transmission of Individual Case Safety Reports". The relevant terms should be coded using ICH "M1: Medical Dictionary for Regulatory Activities (MedDRA)".

According to the "Standards and Procedures for Expedited Reporting of Safety Data during Drug Clinical Trials", for fatal or life-threatening unexpected serious adverse reactions, the applicant should report as soon as possible after first awareness, but no later than 7 days, with follow-up information reported within the next 8 days. For non-fatal or non-life-threatening unexpected serious adverse reactions, the applicant should report as soon as possible after first awareness, but no later than 15 days.

**Reporting Pathways:**

1) National Medical Products Administration (NMPA) Center for Drug Evaluation (CDE) Reporting Method

Transmission Method: Individual safety report electronic documents must meet ICH E2B(R3) requirements and can be transmitted via GATEWAY/XML file transfer.

2) National Health Commission

- Postal Code: 100810
- Phone: 010-68792201
- Fax: 010-68792734
- Email: saefax@163.com (attachments must be in PDF format)

3) Ethics Committee of Beijing Shijitan Hospital

- Phone/Fax: 010-63926342
- Address: 10 Tielu Road, Yangfangdian, Haidian District, Beijing
- Email: bjsjtyy@sina.com

4) Drug Clinical Trial Institution Office of Beijing Shijitan Hospital

- Phone: 010-63926343
- Address: 10 Tielu Road, Yangfangdian, Haidian District, Beijing
- Email: bjsjtyygb@163.com

5) Researcher: Xueyan Wang

- Phone: 15391378519
- Address: 10 Tielu Road, Yangfangdian, Haidian District, Beijing
- Email: Wanxueyan2018@163.com

*Note: SUSARs for each sub-center should be reported by the sponsor according to the requirements of each sub-center.*

**8.14 Pregnancy Events**

Pregnancy itself is not considered an adverse event, but medical events observed in the mother or fetus/newborn will be classified as adverse events.

If a female subject or the partner of a male subject becomes pregnant during the administration of the investigational drug and for six months after the last administration (consistent with the contraceptive period in the inclusion/exclusion criteria), the investigator must immediately fill out a pregnancy event form and submit it to the sponsor. Pregnancy events need to be followed up to one month after the pregnancy outcome.

Any serious adverse event occurring during pregnancy must be recorded in the serious adverse event report form (e.g., severe maternal complications, therapeutic abortion, ectopic pregnancy, stillbirth, neonatal death, congenital anomalies, birth defects) and reported according to the serious adverse event reporting procedures.

All neonatal deaths occurring within one month of birth, regardless of cause, should be reported as SAEs. Additionally, any infant death after one month of age should also be reported if the investigator believes the death may be related to the investigational drug.

**9 Data Management**

This project utilizes an electronic data capture management system.

eCRF Design: The data manager designs the eCRF according to the protocol, which includes all specified data points except external data. The eCRF is exported directly from the EDC system in PDF format.

Data Management Plan (DMP): Based on the finalized protocol and project contract, the data manager drafts the DMP. The DMP is a dynamic document that can be modified and updated during the trial as necessary.

Data Verification Plan (DVP): The data manager prepares the DVP for data verification based on the clinical trial protocol, project contract, and eCRF.

Database Establishment: Database builders create the database, configure logical check rules, and set up system functionalities. System administrators manage permissions based on user roles.

Database Testing: Database testing includes data entry, export, logical checks, eCRF interface, and system functionality tests.

Training: The data manager provides training to relevant personnel, covering system operation skills and/or project requirements. Specific training content depends on personnel roles and prior experience.

Database Deployment: Once database testing is complete and all preparations are finalized, and confirmed by the sponsor, the database is officially deployed.

Data Collection: Researchers or their authorized CRCs enter data into the data management system using independent accounts.

Source Data Verification (SDV): Clinical monitors are responsible for 100% SDV to verify information entered into the database against source data.

Data Verification: Data managers, medical personnel, statisticians, etc., collectively conduct data review according to the DVP. Any issues identified during review are queried as necessary. Researchers or authorized CRCs respond to queries, which are closed after confirmation by the query issuer. If issues persist, queries are reopened until resolved.

Data Review and Blinded Review: Depending on the project, data reviews are conducted during data management, and issues discovered during these reviews are addressed. Before finalizing the database, a blinded review is conducted.

Database Locking: At the end of the trial, after completion of the database lock checklist review, key researchers, sponsors, statisticians, data managers, monitors, and other relevant personnel jointly approve the database locking. If significant data errors are found after locking, a joint assessment is made regarding their potential impact on safety and efficacy analyses. If deemed significant, the database may be unlocked for correction; otherwise, records are maintained in statistical analysis and clinical summary reports.

Data Management Report (DMR): The data manager summarizes the data management process, operational specifications, and management quality in the DMR.

Quality Assurance in Data Management: Quality assurance measures include pre-deployment QC, post-deployment data checks, and pre-lock quality assessments to ensure the quality of data and data management processes.

**10 Statistical Analysis**

**10.1 Sample Size**

The primary efficacy endpoint of this study is the mean total daily nasal symptom score during peak pollen season (PPP). Assuming α=0.025 (one-sided), power (1-β)=0.8, non-inferiority margin of 0, and a mean difference (experimental group - control group) of -1 in the mean total daily nasal symptom score during PPP, with a standard deviation of 2. The ratio of experimental group to control group is 2:1. Using PASS (Version 22) software for calculation, the sample size is 146 subjects (97 in the experimental group and 49 in the control group). Considering approximately 20% dropout rate, a total of 180 subjects are needed (120 in the experimental group and 60 in the control group).

**10.2 Analysis Sets**

Intention-to-Treat Analysis Set (ITT): According to the intention-to-treat principle, this set includes all randomized subjects.

Pharmacokinetic Concentration Set (PKCS): Includes data from subjects who had at least one valid blood drug concentration result after receiving the investigational drug, used for pharmacokinetic concentration analysis.

Pharmacokinetic Parameter Set (PKPS): Includes pharmacokinetic parameter data from subjects who received the study drug at least once, used for descriptive statistical analysis of pharmacokinetic parameters of subjects.

Pharmacodynamic Data Set (PDDS): Includes data from all randomized subjects who received the study drug and had at least one valid pharmacodynamic endpoint data during the trial.

Safety Set (SS): Includes data from all randomized subjects who received the study drug and had safety endpoint records.

Anti-drug Antibody Analysis Set (ADAS): Includes data from subjects who received at least one dose of the study drug and had at least one measurable immunogenicity result. ADAS will be used for immunogenicity analysis.

**10.3** **Hypothesis Testing**

Comparison type: superiority test; significance level: one-sided 0.025.

Null hypothesis H_0_：*μ_1_*-*μ_0_*≥ δ

Alternative hypothesis H_1_：*μ_1_-μ_0_*＜ δ

Where *μ1* is the mean total daily nasal symptom score during peak pollen season (PPP) for the experimental group, *μ0* is the mean total daily nasal symptom score during PPP for the control group, and δ is the superiority margin, δ = 0.

If the upper limit of the (1-2α)% confidence interval for the difference between the two groups (experimental group - control group) is < δ, then H0 is rejected, and H1 is accepted.

**10.4 General Principles**

Unless otherwise specified, all statistical tests are two-sided with a significance level of 0.05. Descriptive statistics for continuous variables include the number of cases, mean, standard deviation, median, quartiles, minimum, and maximum values. Categorical variables are described using counts and percentages.

**10.5 Subject Distribution**

Tables and flowcharts describe the number and percentage of subjects screened, randomized, and completed the trial.

Tables describe the number and percentage of subjects in each analysis set.

Lists of subjects who withdrew from the trial early and those not included in each data set.

Tables describe protocol deviations for each subject.

**10.6 Demographic and Baseline Characteristics Analysis**

Uses ITT analysis.

Describes demographic and baseline characteristics. For continuous variables, calculate the number of cases, mean, standard deviation, median, maximum, and minimum values. For categorical and ordinal variables, calculate the number of cases and percentages.

**10.7 Compliance Analysis**

The calculation method for drug compliance is as follows: Drug compliance (%) = (actual dose / planned dose) × 100%.

**10.8 Concomitant Medications**

The frequency of concomitant medications is summarized according to ATC classification.

**10.9 Efficacy Analysis**

**10.9.1 Primary Estimation Objectives**

Statistical analyses is based on ITT.

**1) Main Statistical Analyses of Primary Estimation Objectives**

The mean total daily nasal symptom scores during peak pollen season (PPP) for the two groups will be compared using a t-test.

Sensitivity analysis explores the robustness of statistical inferences made by the primary estimation method against deviations and limitations of the data. Sensitivity analysis considers one assumption at a time to avoid difficulty in determining which assumption causes the differences in results. Specific strategies are detailed in the statistical analysis plan.

Supplementary Analysis: Missing values will not be imputed, and hypothetical strategies will not be imputed in supplementary analysis.

**2) Primary Estimation Objectives Defined as Follows:**

**Target Population:** Patients with moderate to severe seasonal allergic rhinitis whose symptoms are inadequately controlled under standard-of-care treatment as defined by the protocol inclusion and exclusion criteria.

**Treatment:** Randomly assigned study drug 100 mg/dose, study drug 200 mg/dose, or placebo, administered once every 4 weeks for a total of two doses.

**Target Endpoint:** Mean total daily nasal symptom score (TNSS) during peak pollen season (PPP).

**Concomitant Events/Treatment Strategies:**

| **Concomitant Event** | **Treatment Strategy** | **Notes** |
| --- | --- | --- |
| Early termination of treatment | **Therapy strategy**  Data collection and usage continue even if a concomitant event occurs. | Reflecting clinical practice |
| Use of rescue medication or prohibited medication as determined by data review | **Hypothetical strategy**  Data after the concomitant event is not used and is imputed accordingly. | This does not reflect the actual efficacy of the study drug. |

**Summary at the Population Level**: The difference in mean total daily nasal symptom scores between the two groups during peak pollen season (PPP).

**10.9.2 Secondary Estimation Objectives**

**1) Statistical Analysis of Secondary Estimation Objectives**

The mean total daily nasal symptom score and rescue medication treatment score during peak pollen season (PPP) (DNSMS) will be compared between the two groups using a t-test.

The mean total daily nasal symptom score and rescue medication treatment score during the entire pollen season (PP) (DNSMS) will also be compared between the two groups using a t-test.

The mean total daily ocular symptom score and rescue medication treatment score during the entire pollen season (PP) (DNOMS) will be compared between the two groups using a t-test.

Changes from baseline in Rhinoconjunctivitis Quality of Life Questionnaire (RQLQ) scores at D1, D29, and D57 follow-up visits during the entire pollen season (PP) will be compared between the two groups using a repeated measures mixed-effects model.

The mean daily rescue medication treatment score during peak pollen season (PPP), the mean daily rescue medication treatment score during the entire pollen season (PP), the number of days without nasal symptoms during the entire pollen season (PP), the number of days without using rescue medication during the entire pollen season (PP), and the amount of rescue medication used during the entire pollen season (PP) will be compared between the two groups using the rank-sum test.

**2）Secondary Estimation Objectives Defined as Follows:**

**Secondary Estimation Objective 1:**

**Target Population:** Patients with moderate to severe seasonal allergic rhinitis whose symptoms are inadequately controlled under standard-of-care treatment as defined by the protocol inclusion and exclusion criteria.

**Treatment:** Randomly assigned study drug 100 mg/dose, study drug 200 mg/dose, or placebo, administered once every 4 weeks for a total of two doses.

**Target Endpoint:** The mean total daily nasal symptom score and rescue medication treatment score (DNSMS) during peak pollen season (PPP).

**Concomitant Events/Treatment Strategies:**

| **Concomitant Event** | **Treatment Strategy** | **Notes** |
| --- | --- | --- |
| Early termination of treatment | **Therapy strategy**  Data collection and usage continue even if a concomitant event occurs. | Reflecting clinical practice |
| Use of prohibited medication as determined by data review | **Hypothetical strategy**  Data after the concomitant event is not used and is imputed accordingly. | This does not reflect the actual efficacy of the study drug. |

**Summary at the Population Level**: The difference in mean total daily nasal symptom score and rescue medication treatment score (DNSMS) between the two groups during peak pollen season (PPP).

**Secondary Estimation Objective 2:**

**Target Population:** Patients with moderate to severe seasonal allergic rhinitis whose symptoms are inadequately controlled under standard-of-care treatment as defined by the protocol inclusion and exclusion criteria.

**Treatment:** Randomly assigned study drug 100 mg/dose, study drug 200 mg/dose, or placebo, administered once every 4 weeks for a total of two doses.

**Target Endpoint:** The mean total daily nasal symptom score and rescue medication treatment score (DNSMS) during the entire pollen season (PP).

**Concomitant Events/Treatment Strategies:**

| **Concomitant Event** | **Treatment Strategy** | **Notes** |
| --- | --- | --- |
| Early termination of treatment | **Therapy strategy**  Data collection and usage continue even if a concomitant event occurs. | Reflecting clinical practice |
| Use of prohibited medication as determined by data review | **Hypothetical strategy**  Data after the concomitant event is not used and is imputed accordingly. | This does not reflect the actual efficacy of the study drug. |

**Summary at the Population Level**: The difference in mean total daily nasal symptom score and rescue medication treatment score (DNSMS) between the two groups during the entire pollen season (PP).

**Secondary Estimation Objective 3:**

**Target Population:** Patients with moderate to severe seasonal allergic rhinitis whose symptoms are inadequately controlled under standard-of-care treatment as defined by the protocol inclusion and exclusion criteria.

**Treatment:** Randomly assigned study drug 100 mg/dose, study drug 200 mg/dose, or placebo, administered once every 4 weeks for a total of two doses.

**Target Endpoint:** The mean total daily ocular symptom score and rescue medication treatment score (DNOMS) during the entire pollen season (PP).

**Concomitant Events/Treatment Strategies:**

| **Concomitant Event** | **Treatment Strategy** | **Notes** |
| --- | --- | --- |
| Early termination of treatment | **Therapy strategy**  Data collection and usage continue even if a concomitant event occurs. | Reflecting clinical practice |
| Use of prohibited medication as determined by data review | **Hypothetical strategy**  Data after the concomitant event is not used and is imputed accordingly. | This does not reflect the actual efficacy of the study drug. |

**Summary at the Population Level**: The difference in mean total daily ocular symptom score and rescue medication treatment score (DNOMS) between the two groups during the entire pollen season (PP).

**Secondary Estimation Objective 4:**

**Target Population:** Patients with moderate to severe seasonal allergic rhinitis whose symptoms are inadequately controlled under standard-of-care treatment as defined by the protocol inclusion and exclusion criteria.

**Treatment:** Randomly assigned study drug 100 mg/dose, study drug 200 mg/dose, or placebo, administered once every 4 weeks for a total of two doses.

**Target Endpoint:** The mean daily rescue medication treatment score during peak pollen season (PPP).

**Concomitant Events/Treatment Strategies:**

| **Concomitant Event** | **Treatment Strategy** | **Notes** |
| --- | --- | --- |
| Early termination of treatment | **Therapy strategy**  Data collection and usage continue even if a concomitant event occurs. | Reflecting clinical practice |
| Use of prohibited medication as determined by data review | **Hypothetical strategy**  Data after the concomitant event is not used and is imputed accordingly. | This does not reflect the actual efficacy of the study drug. |

**Summary at the Population Level**: The difference in mean daily rescue medication treatment score between the two groups during peak pollen season (PPP).

**Secondary Estimation Objective 5:**

**Target Population:** Patients with moderate to severe seasonal allergic rhinitis whose symptoms are inadequately controlled under standard-of-care treatment as defined by the protocol inclusion and exclusion criteria.

**Treatment:** Randomly assigned study drug 100 mg/dose, study drug 200 mg/dose, or placebo, administered once every 4 weeks for a total of two doses.

**Target Endpoint:** The mean daily rescue medication treatment score during the entire pollen season (PP).

**Concomitant Events/Treatment Strategies:**

| **Concomitant Event** | **Treatment Strategy** | **Notes** |
| --- | --- | --- |
| Early termination of treatment | **Therapy strategy**  Data collection and usage continue even if a concomitant event occurs. | Reflecting clinical practice |
| Use of prohibited medication as determined by data review | **Hypothetical strategy**  Data after the concomitant event is not used and is imputed accordingly. | This does not reflect the actual efficacy of the study drug. |

**Summary at the Population Level**: The difference in mean daily rescue medication treatment score between the two groups during the entire pollen season (PP).

**Secondary Estimation Objective 6:**

**Target Population:** Patients with moderate to severe seasonal allergic rhinitis whose symptoms are inadequately controlled under standard-of-care treatment as defined by the protocol inclusion and exclusion criteria.

**Treatment:** Randomly assigned study drug 100 mg/dose, study drug 200 mg/dose, or placebo, administered once every 4 weeks for a total of two doses.

**Target Endpoint:** Changes from baseline in Rhinoconjunctivitis Quality of Life Questionnaire (RQLQ) scores at D1, D29, and D57 follow-up visits during the entire pollen season (PP).

**Concomitant Events/Treatment Strategies:**

| **Concomitant Event** | **Treatment Strategy** | **Notes** |
| --- | --- | --- |
| Early termination of treatment | **Therapy strategy**  Data collection and usage continue even if a concomitant event occurs. | Reflecting clinical practice |
| Use of rescue medication or prohibited medication as determined by data review | **Hypothetical strategy**  Data after the concomitant event is not used and is imputed accordingly. | This does not reflect the actual efficacy of the study drug. |

**Summary at the Population Level**: Differences in changes from baseline in Rhinoconjunctivitis Quality of Life Questionnaire (RQLQ) scores at D1, D29, and D57 follow-up visits between the two groups during the entire pollen season (PP).

**Secondary Estimation Objective 7:**

**Target Population:** Patients with moderate to severe seasonal allergic rhinitis whose symptoms are inadequately controlled under standard-of-care treatment as defined by the protocol inclusion and exclusion criteria.

**Treatment:** Randomly assigned study drug 100 mg/dose, study drug 200 mg/dose, or placebo, administered once every 4 weeks for a total of two doses.

**Target Endpoint:** Number of days without nasal symptoms in subjects during the entire pollen season (PP).

**Concomitant Events/Treatment Strategies:**

| **Concomitant Event** | **Treatment Strategy** | **Notes** |
| --- | --- | --- |
| Early termination of treatment | **Therapy strategy**  Data collection and usage continue even if a concomitant event occurs. | Reflecting clinical practice |
| Use of rescue medication or prohibited medication as determined by data review | **Hypothetical strategy**  Data after the concomitant event is not used and is imputed accordingly. | This does not reflect the actual efficacy of the study drug. |

**Summary at the Population Level**: Differences in the number of days without nasal symptoms between the two groups during the entire pollen season (PP).

**Secondary Estimation Objective 8:**

**Target Population:** Patients with moderate to severe seasonal allergic rhinitis whose symptoms are inadequately controlled under standard-of-care treatment as defined by the protocol inclusion and exclusion criteria.

**Treatment:** Randomly assigned study drug 100 mg/dose, study drug 200 mg/dose, or placebo, administered once every 4 weeks for a total of two doses.

**Target Endpoint:** Number of days without using rescue medication in subjects during the entire pollen season (PP).

**Concomitant Events/Treatment Strategies:**

| **Concomitant Event** | **Treatment Strategy** | **Notes** |
| --- | --- | --- |
| Early termination of treatment | **Therapy strategy**  Data collection and usage continue even if a concomitant event occurs. | Reflecting clinical practice |
| Use of prohibited medication as determined by data review | **Hypothetical strategy**  Data after the concomitant event is not used and is imputed accordingly. | This does not reflect the actual efficacy of the study drug. |

**Summary at the Population Level:** Differences in the number of days without using rescue medication between the two groups during the entire pollen season (PP).

**Secondary Estimation Objective 9:**

**Target Population:** Patients with moderate to severe seasonal allergic rhinitis whose symptoms are inadequately controlled under standard-of-care treatment as defined by the protocol inclusion and exclusion criteria.

**Treatment:** Randomly assigned study drug 100 mg/dose, study drug 200 mg/dose, or placebo, administered once every 4 weeks for a total of two doses.

**Target Endpoint:** Amount of rescue medication used by subjects during the entire pollen season (PP).

**Concomitant Events/Treatment Strategies:**

| **Concomitant Event** | **Treatment Strategy** | **Notes** |
| --- | --- | --- |
| Early termination of treatment | **Therapy strategy**  Data collection and usage continue even if a concomitant event occurs. | Reflecting clinical practice |
| Use of prohibited medication as determined by data review | **Hypothetical strategy**  Data after the concomitant event is not used and is imputed accordingly. | This does not reflect the actual efficacy of the study drug. |

**Summary at the Population Level:** Differences in the amount of rescue medication used between the two groups during the entire pollen season (PP).

**10.10 Safety Analyses**

安全性分析采用安全性分析集。

**10.10.1 Primary Statistical Analyses of Adverse Events**

Safety analysis utilizes the safety analysis set.

Only adverse events occurring during the treatment period are analyzed for safety in this trial.

Preferred terms and System Organ Class (SOC) classifications will be encoded using MedDRA (version 25.1 or above).

Post-treatment adverse events are defined as those occurring or worsening after the first administration of the investigational drug.

Adverse events, adverse reactions, serious adverse events, serious adverse reactions, adverse events leading to withdrawal, and adverse reactions leading to withdrawal will be summarized by group for the following: occurrences, number of cases, and incidence rates. They will also be summarized by formulation group, SOC, and Preferred Term (PT) for the occurrences, number of cases, and incidence rates of adverse events, adverse reactions, serious adverse events, serious adverse reactions, adverse events leading to withdrawal, and adverse reactions leading to withdrawal. Furthermore, they will be summarized by group, severity, SOC, and PT for occurrences, number of cases, and incidence rates of adverse events and adverse reactions. The statistical analysis of adverse event incidence will ensure that each subject is counted no more than once per SOC and PT.

**10.10.2 Primary Statistical Analyses of Clinical Safety Evaluation**

Observations of safety data such as vital signs and changes relative to baseline (if applicable) will be descriptively analyzed by treatment group and/or time points.

Changes in clinical assessments like laboratory tests, 12-lead electrocardiograms, and physical examinations pre- and post-administration will be cross-tabulated. Lists of laboratory tests, 12-lead electrocardiogram results, and abnormal findings for all subjects will be provided.

**10.10.3 Pharmacokinetic and Pharmacodynamic Analyses**

Descriptive statistics for drug concentration values at each time point will include occurrences, mean, standard deviation, coefficient of variation, minimum, median, maximum, and geometric mean. Blood concentration-time curves will be plotted based on sampling time points and average or individual concentrations.

If data permits, pharmacokinetic analysis will be conducted based on blood concentration data.

Descriptive statistics for pharmacodynamic concentration values at each time point will include occurrences, mean, standard deviation, coefficient of variation, minimum, median, maximum, and geometric mean. Individual and average pharmacodynamic intensity (E)-time (t) curves will be plotted based on sampling time points.

**10.10.4 Immunogenicity Analyses**

Descriptive statistics will be used to summarize immunogenicity data at different time points and calculate positivity rates.

**10.10.5 Exploratory Analyses**

Exploratory analysis will investigate exposure-response relationships following multiple administrations of different doses of LP-003 injection.

**10.11 Interim Analyses**

This study is exploratory, and if necessary, interim analyses will be conducted in due course. Considering the exploratory nature of the trial, interim analyses will not consume Type I error.

**10.12 Multiple Testing Adjustment**

As this study is exploratory, no multiple testing adjustments will be applied.

**10.13 Handling of Missing, Unused, and Illogical Data**

Details on handling missing data are outlined in the statistical analysis plan.

Generally, all data will be used for statistical analysis or listed.

Illogical data will be discussed during data review meetings to decide whether to exclude them based on specific circumstances or conduct sensitivity analyses.

**10.14 Procedures for Deviations from the Planned Statistical Analysis Plan**

Updates to the Statistical Analysis Plan (SAP) may occur due to updates in the clinical trial protocol or eCRF versions, but the SAP must be finalized before database lock. After database lock, any deviations from the original SAP will only be considered for sensitivity or supplementary analyses.

**10.15 Analysis Software and General Requirements**

WinNonLin (version 8.3 or above) will be used for pharmacokinetic parameter calculations, while SAS (version 9.4 or above) will be used for other analyses. For detailed statistical analysis plans, refer to the SAP.

**11 Quality Control and Quality Assurance**

**11.1 Clinical Monitoring**

The sponsor is responsible for organizing and conducting this clinical trial, including clinical monitoring and auditing. Qualified clinical monitors will be regularly assigned to visit clinical trial sites to ensure compliance with GCP and the protocol, and to verify the authenticity, completeness, and accuracy of the raw data. Investigators will cooperate with clinical monitors to address any issues identified during monitoring visits. After each monitoring visit, the clinical monitor must write a monitoring report and/or a follow-up letter to the investigator.

Prior to the initiation of the clinical trial (e.g., during site initiation visits or investigator meetings), the sponsor's representative or designated personnel will review the trial protocol and other standard operating procedures with the investigator and study staff.

The eCRF is not the original document. All data entered in the case report form must be traceable back to the original records (electronic or paper) in the subject's file or electronic database.

During the trial, monitors will visit clinical trial sites to monitor:

- Completeness, consistency, and accuracy of eCRF entries
- Confirmation of source data
- Quality control of eCRF entries
- Progress of subject enrollment
- Compliance with the protocol and GCP
- Correct storage, dispensation, and accountability of investigational medicinal products according to the protocol

Investigators and key study personnel must cooperate during monitoring visits.

Investigators must retain original records for each trial subject, including medical records and visit records containing demographic and medical information, laboratory data, ECGs, and results of any other assessments. A signed informed consent form must also be retained.

Subjects' identities in original documents must be kept confidential.

Study documents such as the trial protocol, investigator's brochure, protocol amendments, and any other essential regulatory documents must be securely stored together.

Investigators must allow monitors to review all relevant original documents to verify the consistency of data entered in the eCRF with the original documents. According to monitoring standards, it is mandatory to verify 100%:

- Existence of informed consent forms
- Compliance with inclusion/exclusion criteria
- Written records of serious adverse events
- Records of all major endpoint data

Additionally, a specific monitoring plan will be developed for this trial to verify the consistency between eCRF and source data. No information regarding subjects' identities from the original documents should be disclosed.

The sponsor, based on trial progress and the results of quality control personnel/monitors, may conduct audits on various aspects of the clinical trial process, data, reports, and calculations.

Monitors and inspectors from ethics committees and drug regulatory authorities, contracted research organizations appointed by the sponsor, may directly access the trial site to review source data, source documents, and reports.

Investigators (and/or authorized CRCs) are responsible for completing the eCRF, while monitors are responsible for reviewing the eCRF and explaining and assisting in resolving any data queries.

For specific details of clinical monitoring, refer to the monitoring plan document.

**11.2 Quality Control**

Before the start of the clinical trial, all trial personnel must undergo training on the trial protocol. They should thoroughly read and understand the content of this clinical trial protocol, grasp the principles of GCP, and adhere strictly to the protocol in terms of recording methods and judgment criteria.

Quality control will be conducted on all participating clinical trial sites. If specific issues are identified at any clinical trial site, corrective actions must be established.

Qualified designated personnel must execute the quality control measures.

For specific details of quality control, refer to the Quality Control Plan document.

**11.3 Quality Assurance**

Sponsors and investigators should establish their respective quality assurance systems, fulfill their responsibilities, and strictly adhere to the clinical trial protocol using appropriate standard operating procedures to ensure the implementation of quality control and quality assurance systems for the clinical trial.

To ensure that the trial is conducted in accordance with the protocol, GCP guidelines, and all applicable regulations, in addition to routine monitoring procedures, when necessary, the sponsor or a third party delegated by the sponsor will conduct audits of the clinical trial to assess compliance with GCP. For specific details of quality assurance, refer to the Quality Assurance Plan document.

**12 Ethical Requirements**

**12.1 Ethical Committee Approval**

The implementation of this clinical trial must adhere to the Declaration of Helsinki (2013 version), Good Clinical Practice (GCP) guidelines (2020 version), relevant regulations, and the ethical committee's review opinions.

Prior to the trial initiation, the investigator must obtain written approval from the relevant regulatory authorities for the trial protocol, informed consent documents, patient recruitment procedures, and other written materials provided to patients. Ethical committee approval is required before the implementation of this trial can commence. Any modifications to the trial protocol, informed consent documents, or other ethical documents during the clinical trial must be approved again by the ethical committee. The investigator is responsible for regularly submitting trial progress reports as required by the ethical committee and submitting a trial completion report for ethical committee review upon trial conclusion.

**12.2 Informed Consent**

The investigator or their designated representative will be responsible for explaining the background of the study, information related to the investigational product, the trial protocol, and the benefits and risks of participating in the trial to each trial subject, their legal guardian, or witness. Written informed consent signed by the trial subject themselves, their legal guardian, and the investigator must be obtained before the trial subject enters the trial (prior to screening).

The final informed consent document should include: the purpose of the trial, trial procedures, obligations of the trial subject, foreseeable benefits and risks or inconveniences to the trial subject from participating in the trial, provision for treatment and appropriate compensation for trial-related harm, access to trial data, and confidentiality of patient information, among others.

The informed consent document should obtain written approval from the relevant regulatory authorities according to regulations and should be written in a language understandable to the trial subject. The trial subject or their legal guardian, the investigator or their representative conducting the informed consent process must sign and date the informed consent document. The original informed consent document shall be retained by the investigator, and a copy shall be provided to the trial subject. In the event of significant new data related to the investigational product, the informed consent document must be revised in writing, submitted to the relevant regulatory authorities for approval, and the informed consent document must be signed again.

**13** **Risk Assessment and Risk Management**

Considering the safety of the subjects, it is necessary to closely monitor the potential adverse events that subjects may experience during the trial, and promptly take necessary measures as determined by the investigator to ensure the safety of the subjects once adverse reactions occur.

Risk control measures are as follows:

1) **Subject Selection:** Strict adherence to inclusion/exclusion criteria.

2) **Informed Consent:** Clearly inform subjects in the informed consent document about the potential risks associated with participation in this trial.

3) **Emergency Preparedness:** Before the trial begins, ensure that emergency medications are available, within expiration dates, and regularly checked during the trial.

4) **Emergency Plan:** Develop and finalize an emergency plan before the trial begins.

5) **Monitoring and Management of Adverse Events:**Throughout the trial, clinical trial investigators at the clinical trial institution will monitor adverse events. During monitoring, observe subjects for adverse events at any time, and promptly provide symptomatic treatment for any adverse events that occur. Follow-up visits are conducted outside of the monitoring period to determine if there are any other adverse events.

Follow-up is required even after discontinuation of the investigational product if adverse events or their sequelae persist.

1. **Regular Monitoring:** During the trial, investigators regularly check subjects' vital signs and general condition. Promptly take measures to handle and record adverse events if subjects experience them, ensuring subject safety.
2. **Serious Adverse Events:** In the event of a serious adverse event, follow hospital protocols for emergency medical transport.

**14 Study Management**

**14.1 Training**

Before the trial begins, monitors will contact the clinical trial institution to provide training to the staff on the trial protocol, trial requirements, and applicable management regulations, in accordance with the Good Clinical Practice (GCP) guidelines.

**14.2 Clinical Monitoring**

Monitors will conduct regular visits to the clinical trial institution to ensure:

1. The data is genuine, accurate, and complete.
2. The safety and rights of the subjects are protected.
3. The trial is conducted according to the currently approved protocol and GCP guidelines.

Researchers and the head of the medical institution agree to allow monitors direct access to all relevant documents.

监查员将定期对临床试验机构进行监查，以确保：

（1）数据真实、准确、完整；

**14.3 Audits**

To ensure this trial complies with the GCP guidelines (2020 version) and related management regulations, the sponsor and Shanghai RenZhi Medical Research Co., Ltd. may conduct quality assurance audits of the records at the clinical trial institution. During the audit, researchers (and the clinical trial institution) must agree to allow auditors direct access to all relevant documents, and auditors may discuss any issues or related matters with the researchers or other staff at any time. Personnel from the sponsor and the contract research organization authorized by the sponsor may directly access source data, source documents, and reports at the trial site for monitoring and auditing.

**14.4 Closing the Clinical Trial Institution**

According to GCP guidelines and standard operating procedures, monitors should work with researchers or clinical trial institution staff to close the clinical trial institution after the trial is completed or terminated.

If the trial is suspended or terminated due to safety reasons, the sponsor will notify all researchers, heads of medical institutions, and other clinical trial institutions involved in the trial. The sponsor will promptly notify the relevant regulatory authorities of the decision to suspend/terminate the trial and the reasons for it. If required, the researcher or head of the medical institution must also promptly notify the Institutional Ethics Committee (IEC) and provide the reasons for the trial suspension or termination.

**14.5 Recording and Preservation of Study Data**

**14.5.1 Source Data and Source Documents**

In this trial, source data includes clinical findings, observations, and other relevant activity records needed to reconstruct and evaluate the clinical trial. Original data is contained within source documents.

Source documents in clinical research are the original records, documents, and data (e.g., hospital medical records, medical images, laboratory records, memos, subject diaries or evaluation forms, drug dispensing records, instrument-generated data, microfilms, photographic negatives, X-rays, subject files, and documents and records related to clinical trials maintained by pharmacies, laboratories, and medical departments, including certified copies). Source documents must be retained to support the information provided in the electronic Case Report Forms (eCRFs).

**14.5.2 Record Preservation by the Clinical Trial Institution**

**(1) Materials Related to the Ethics Committee**

Personnel responsible for record preservation at the clinical trial institution must retain all records of ethical reviews from the Ethics Committee until 5 years after the trial is terminated or completed. If the sponsor wishes to retain records for a longer period, both parties will discuss and decide on the retention period and method. If there are any changes in the document preservation at the clinical trial institution, the responsible person or researcher needs to contact the sponsor.

**(2) Materials Related to Trial Implementation**

Personnel responsible for record preservation at the clinical trial institution must retain essential clinical trial documents until 5 years after the investigational product is approved for marketing. If the sponsor wishes to retain records for a longer period, both parties will discuss and decide on the retention period and method. If there are any changes in the document preservation at the clinical trial institution, the responsible person or researcher needs to contact the sponsor.

**14.6 Confidentiality**

All information obtained during the trial operations and related to the health status of the subjects will be considered confidential. Disclosure of any such information requires written consent.

Researchers must ensure the anonymity of each subject. Subjects should not be identified by name in submitted eCRFs and other documents. Instead, subjects will be identified by their unique subject code. The assigned subject code is intended to ensure the confidentiality of all research documents. Subjects will retain this unique code throughout the trial. Researchers will maintain an independent log of these codes. Without violating confidentiality principles and relevant regulations, monitors, auditors, Ethics Committees, and drug regulatory authority inspectors can review the subjects' original medical records to verify the trial process and data. Confidential matters related to subject identification records will not be publicly disclosed. If clinical trial results are published, the subjects' identity information will remain confidential.

**14.7 Protocol Amendments**

Any changes or amendments to the trial protocol must receive prior approval from both the sponsor and the Independent Ethics Committee (IEC).

In special circumstances, amendments required to ensure the safety of the subjects can be implemented before IEC approval. In such cases, the investigator must inform the sponsor of the measures taken and notify the IEC of the clinical trial institution within 5 working days. These amendments must also be formally approved as part of the protocol revisions.

| **Protocol/Amendment Number** | **Version Number/Date** | **Amendment Details** |
| --- | --- | --- |
| P10-LP003-02 | V1.0/2023.02.27 | Initial version |

**14.8 Protocol Compliance**

All requirements specified in the trial protocol must be strictly followed. Any intentional or unintentional deviations or violations of the protocol and GCP principles will be classified as protocol deviations or violations. If a deviation is discovered during monitoring, the monitor or investigator must complete a protocol deviation record, detailing the time of discovery, the time and process of the event, the reason, and the corresponding corrective measures. This record must be signed by the investigator and reported to both the IEC and the sponsor. In data statistics and summary reports, the investigator must analyze and report the impact of protocol deviations or violations on the final data and conclusions.

If serious protocol violations occur, an assessment should be conducted. If necessary, the sponsor may terminate the trial prematurely.

**14.9 Registration and Publication of Clinical Trial Results**

With the written consent of the sponsor, the investigator may publish information or results from the clinical trial in scientific journals or other publications, or use them for educational or other research activities.

Materials provided to the investigator by the sponsor are considered non-public information and must be kept confidential. No one is allowed to disclose such information to others without written permission from the sponsor and the clinical trial institution. After the trial is completed, if related trial papers are to be published, they must receive the approval of both the sponsor and the clinical trial institution. The order of authorship will also be decided through mutual agreement.

**14.10 Conflict of Interest Policy**

Clinical trials should not be influenced by any existing or foreseeable factors, such as pharmaceutical companies. Therefore, any individuals involved in the design, execution, analysis, publication, or other aspects of this trial must disclose and manage any actual conflicts of interest. Additionally, during the design and execution of the trial, individuals with potential conflicts of interest will be required to manage such situations appropriately to prevent them from occurring.

**15 Responsibility and Insurance**

**15.1 GCP Responsibility**

The responsibilities of the sponsor, monitors, and investigators are consistent with GCP, relevant guidelines, and Chinese regulatory requirements. Investigators are responsible for adhering to the responsibilities outlined in GCP, including distributing the investigational product according to the approved protocol or signed amendments, and ensuring the safe storage and handling of the investigational product throughout the trial.

**15.2 Benefits to Subjects**

All examinations and trial-related procedures for subjects participating in this trial are free of charge. Subjects enrolled in the trial will be provided with transportation reimbursement for each visit to the hospital. This compensation will be given to subjects based on their progress in completing the trial.

**15.3 Insurance**

The sponsor will purchase clinical trial liability insurance for this clinical trial in accordance with relevant laws and regulations in China. Costs associated with the treatment of adverse events related to the trial will be covered by the sponsor/insurance company.

**16 References**

[1] Guidelines for the Diagnosis and Treatment of Allergic Rhinitis in China (2022, Revised Edition), Nasology Group of the Editorial Committee of the Chinese Journal of Otolaryngology Head and Neck Surgery/Nasology Group of the Otolaryngology Head and Neck Surgery Branch of the Chinese Medical Association.

[2] Ye Jing, Xu Rui, Qiu Qianhui, et al. Expert Consensus on Anti-IgE Monoclonal Antibody Treatment for Allergic Rhinitis (2022, Nanchang) [J]. Chinese Journal of Clinical Immunology and Allergy, 2022, 16(05):458-468.

[3] Maurer M, Giménez-Arnau A, Sussman G, et al. Ligelizumab for Chronic Spontaneous Urticaria [J]. N Engl J Med, 2019, 381:1321-32.

[4] Harris1 JM, Maciuca1 R, Bradley MS, et al. A randomized trial of the efficacy and safety of quilizumab in adults with inadequately controlled allergic asthma [J]. Respiratory Research 2016; 17:29.

[5] Okubo K , Okano M , Sato N , et al. Add-On Omalizumab for Inadequately Controlled Severe Pollinosis Despite Standard-of-Care: A Randomized Study[J]. The Journal of Allergy and Clinical Immunology In Practice, 2020, 8(9).

[6] Package Insert for Omalizumab Injection. Xolair®.

[7] Arm JP, Bottoli I, Skerjanec A, Floch D, Groenewegen A, Maahs S, Owen CE, Jones I, Lowe PJ. Pharmacokinetics, pharmacodynamics and safety of QGE031 (ligelizumab), a novel high-affinity anti-IgE antibody, in atopic subjects. Clin Exp Allergy. 2014 Nov;44(11):1371-85.

[8] Wedi B. Ligelizumab for the treatment of chronic spontaneous urticaria.[J].Expert Opin Biol Ther. 2020 Aug;20(8):853-861.

[9] Novartis provides an update on Phase III ligelizumab (QGE031) studies in chronic spontaneous urticaria (CSU).Retrieved Dec 20, 2021.From https://www.novartis.com/news/media-releases/novartis-provides-update-phase-iii-ligelizumab-qge031-studies-chronic-spontaneous-urticaria-csu

[10] Technical Review Report for the Marketing Application of Omalizumab Injection (JXSS1400005) - CDE.

**Appendix 1: Contraception Measures, Definition of Women of Childbearing Potential, and Contraception Requirements**

**1. Definition of Women of Childbearing Potential**

Women who are over 54 years old and have been amenorrheic for ≥12 months, or those who have undergone a hysterectomy, bilateral oophorectomy, or have medically confirmed ovarian failure at any age, are considered non-childbearing potential women.

Women who have not undergone hysterectomy or bilateral oophorectomy and do not have medically confirmed ovarian failure, aged ≤54 years (including those with any duration of amenorrhea), are considered women of childbearing potential.

**2. Contraception Requirements for Female Participants (and Their Male Partners)**

- Before inclusion, women of childbearing potential must have a blood pregnancy test at screening that meets trial requirements. From the start of the trial drug administration until 6 months after the last dose of the trial drug, you and/or your partner must use at least one of the following contraceptive methods:
- The preferred lifestyle is complete abstinence, not accepting periodic abstinence (e.g., calendar method, ovulation method, symptothermal method, post-ovulation method).
- Partner vasectomy or removal (at least 90 days from the date of surgery).
- Intrauterine device (failure rate <1%).
- Double protection method (condom, contraceptive sponge, diaphragm/cervical cap with spermicide gel or cream).
- Menopause for 2 years, surgical sterilization (bilateral tubal ligation, bilateral oophorectomy, or hysterectomy). Postmenopausal definition: more than 2 years since menopause, appropriate age, and follicle-stimulating hormone levels indicating postmenopausal status (confirmed during the screening period).

**3. Contraception Requirements for Male Participants (and Their Female Partners)**

From the first dose of the trial drug to 6 months after the last dose, all male participants must agree to consistently and correctly use condoms to prevent sperm donation. If their female partner is of childbearing potential (as defined above), she must use one of the contraceptive methods listed above from the first dose of the trial drug to 6 months after the last dose.

- For men, you must undergo surgical sterilization (at least 90 days from the date of surgery) or you and/or your partner must use at least one of the following contraceptive methods:
- The preferred lifestyle is complete abstinence, not accepting periodic abstinence.
- Partner use of intrauterine device (failure rate <1%).
- Partner use of oral, injectable, vaginal, or implantable hormonal contraceptives.
- Double protection method (condom, contraceptive sponge, diaphragm/cervical cap with spermicide gel or cream).
- Male participants must agree to avoid sperm donation for 6 months after the last dose of the trial drug.

**4. Procedures to Follow in Case of Pregnancy**

If a participant (or their partner) becomes pregnant at any time during the trial or if a participant becomes pregnant within 6 months after the last dose of the trial drug (or if the partner of a male participant becomes pregnant within 6 months), the participant will notify the researcher as instructed. Pregnant or suspected pregnant participants must immediately report this information to the researcher and discontinue the trial drug. The pregnant partner of a participant or the pregnant participant must report this information immediately.

**Appendix 2: Laboratory Tests**

| Complete Blood Count | Red Blood Cell (RBC) Count, Hemoglobin (HGB), Platelet (PLT) Count, White Blood Cell (WBC) Count, Neutrophil (Neu) Count, Lymphocyte (Lym) Count, Eosinophil Count, Basophil Count |
| --- | --- |
| Blood Biochemistry | Alanine Aminotransferase (ALT), Aspartate Aminotransferase (AST), Alkaline Phosphatase (ALP), Gamma-Glutamyl Transferase (GGT), Total Bilirubin (TBIL), Direct Bilirubin (DBIL), Total Protein (TP), Albumin (ALB), Urea or Blood Urea Nitrogen (BUN), Creatinine (Cr), Creatine Kinase Isoenzyme (CK), Lactate Dehydrogenase (LDH), Blood Glucose (GLU), Potassium (K+), Sodium (Na+), Chloride (Cl-), Calcium (Ca2+), Magnesium (Mg2+), Uric Acid (UA) |
| Urinalysis | Glucose (GLU), Ketones (KET), Bilirubin (BIL), Urobilinogen (UBG), Protein (PRO), pH, Red Blood Cells, White Blood Cells |
| Coagulation Function | Prothrombin Time (PT), Thrombin Time (TT), Activated Partial Thromboplastin Time (APTT), Fibrinogen (FIB) |
| Pregnancy Test | Human Chorionic Gonadotropin (HCG) |

**Appendix 3: Rhinoconjunctivitis Quality of Life Questionnaire (RQLQ)**

**Rhinoconjunctivitis Quality of Life Questionnaire**

**Activities**

We would like you to think back over the past 7 days and recall how your nasal/eye symptoms have affected your life. We want to understand which activities you engaged in that were limited by your nasal/eye symptoms. This limitation means you did these activities less, or not as well, or did not enjoy them as much as usual. These activities should be ones you do regularly, are important to your daily life, and are things you will frequently do throughout the study.

Below is a list of activities that might be limited by nasal/eye symptoms. We hope this helps you identify the three main activities that have been restricted in the past 7 days due to your nasal/eye symptoms.

| 1、Riding a bicycle | 2、Reading | 3、Shopping |
| --- | --- | --- |
| 4、Doing household repairs | 5、Housework | 6、Entering or exiting air-conditioned rooms |
| 7、Watching TV | 8、Exercising or working out | 9、Morning exercises |
| 10、Using a computer | 11、Playing table tennis | 12、Playing with pets |
| 13、Playing with children or grandchildren | 14、Participating in team sports | 15、Driving |
| 16、Singing | 17、Engaging in normal social activities | 18、Sexual activity |
| 19、Playing badminton | 20、Chatting | 21、Eating |
| 22、Using a vacuum cleaner | 23、Visiting friends or relatives | 24、Going for a walk |
| 25、Taking children to and from school | 26、Outdoor activities | 27、Working |
| 28、Sitting outside | 29、Taking children to the park | 30、Being in a smoking environment |

Please write down your top 3 main activities on the lines below and use the checkboxes to indicate how much each activity has been affected by your nasal/eye symptoms over the past 7 days.

|  | No Trouble | Almost No Trouble | Some Trouble | Moderate Trouble | Quite a Bit of Trouble | A Lot of Trouble | Extremely Troubled | Did Not Do Activity |
| --- | --- | --- | --- | --- | --- | --- | --- | --- |
|  | 0 | 1 | 2 | 3 | 4 | 5 | 6 | 9 |
| 1、 | **□** | **□** | **□** | **□** | **□** | **□** | **□** | **□** |
| 2、 | **□** | **□** | **□** | **□** | **□** | **□** | **□** | **□** |
| 3、 | **□** | **□** | **□** | **□** | **□** | **□** | **□** | **□** |

**Sleep**

Over the past 7 days, to what extent have your nasal/eye symptoms troubled you with the following sleep problems?

|  | No Trouble | Almost No Trouble | Some Trouble | Moderate Trouble | Quite a Bit of Trouble | A Lot of Trouble | Extremely Troubled |
| --- | --- | --- | --- | --- | --- | --- | --- |
|  | 0 | 1 | 2 | 3 | 4 | 5 | 6 |
| 4、Difficulty falling asleep | **□** | **□** | **□** | **□** | **□** | **□** | **□** |
| 5、Waking up during the night | **□** | **□** | **□** | **□** | **□** | **□** | **□** |
| 6、Poor sleep at night | **□** | **□** | **□** | **□** | **□** | **□** | **□** |

**Non-Nasal/Eye Symptoms**

In the past 7 days, to what extent have you been troubled by the following issues due to your nasal/eye symptoms?

|  | No Trouble | Almost No Trouble | Some Trouble | Moderate Trouble | Quite a Bit of Trouble | A Lot of Trouble | Extremely Troubled |
| --- | --- | --- | --- | --- | --- | --- | --- |
|  | 0 | 1 | 2 | 3 | 4 | 5 | 6 |
| 7、Lack of energy | **□** | **□** | **□** | **□** | **□** | **□** | **□** |
| 8、Thirst | **□** | **□** | **□** | **□** | **□** | **□** | **□** |
| 9、Decreased work ability | **□** | **□** | **□** | **□** | **□** | **□** | **□** |
| 10、Fatigue | **□** | **□** | **□** | **□** | **□** | **□** | **□** |
| 11、Difficulty concentrating | **□** | **□** | **□** | **□** | **□** | **□** | **□** |
| 12、Headache | **□** | **□** | **□** | **□** | **□** | **□** | **□** |
| 13、Exhaustion | **□** | **□** | **□** | **□** | **□** | **□** | **□** |

**Practical Problems**

In the past 7 days, to what extent have you been troubled by the following issues due to your nasal/eye symptoms?

|  | No Trouble | Almost No Trouble | Some Trouble | Moderate Trouble | Quite a Bit of Trouble | A Lot of Trouble | Extremely Troubled |
| --- | --- | --- | --- | --- | --- | --- | --- |
|  | 0 | 1 | 2 | 3 | 4 | 5 | 6 |
| 14、Inconvenience due to needing to carry tissues or handkerchiefs | **□** | **□** | **□** | **□** | **□** | **□** | **□** |
| 15、Need to rub your nose/eyes | **□** | **□** | **□** | **□** | **□** | **□** | **□** |
| 16、Need to blow your nose repeatedly | **□** | **□** | **□** | **□** | **□** | **□** | **□** |

**Nasal Symptoms**

In the past 7 days, to what extent have you been troubled by the following symptoms?

|  | No Trouble | Almost No Trouble | Some Trouble | Moderate Trouble | Quite a Bit of Trouble | A Lot of Trouble | Extremely Troubled |
| --- | --- | --- | --- | --- | --- | --- | --- |
|  | 0 | 1 | 2 | 3 | 4 | 5 | 6 |
| 17、Nasal congestion/blockage | **□** | **□** | **□** | **□** | **□** | **□** | **□** |
| 18、Runny nose | **□** | **□** | **□** | **□** | **□** | **□** | **□** |
| 19、Sneezing | **□** | **□** | **□** | **□** | **□** | **□** | **□** |
| 20、Post-nasal drip | **□** | **□** | **□** | **□** | **□** | **□** | **□** |

**Eye Symptoms**

In the past 7 days, to what extent have you been troubled by the following symptoms?

|  | No Trouble | Almost No Trouble | Some Trouble | Moderate Trouble | Quite a Bit of Trouble | A Lot of Trouble | Extremely Troubled |
| --- | --- | --- | --- | --- | --- | --- | --- |
|  | 0 | 1 | 2 | 3 | 4 | 5 | 6 |
| 21、Itchy eyes | **□** | **□** | **□** | **□** | **□** | **□** | **□** |
| 22、Watery eyes | **□** | **□** | **□** | **□** | **□** | **□** | **□** |
| 23、Eye pain | **□** | **□** | **□** | **□** | **□** | **□** | **□** |
| 24、Swollen eyes | **□** | **□** | **□** | **□** | **□** | **□** | **□** |

**Emotions**

In the past 7 days, how often have you been troubled by the following emotional problems due to your nasal/eye symptoms?

|  | No Trouble | Almost No Trouble | Some Trouble | Moderate Trouble | Quite a Bit of Trouble | A Lot of Trouble | Extremely Troubled |
| --- | --- | --- | --- | --- | --- | --- | --- |
|  | 0 | 1 | 2 | 3 | 4 | 5 | 6 |
| 25、Feeling upset | **□** | **□** | **□** | **□** | **□** | **□** | **□** |
| 26、Feeling impatient or restless | **□** | **□** | **□** | **□** | **□** | **□** | **□** |
| 27、Feeling irritable | **□** | **□** | **□** | **□** | **□** | **□** | **□** |
| 28、Feeling embarrassed due to symptoms | **□** | **□** | **□** | **□** | **□** | **□** | **□** |
