# Supplementary material for: LP‐003, a novel high‐affinity anti‐IgE antibody for inadequately controlled seasonal allergic rhinitis: A multicenter, randomized, double‐blind, placebo‐controlled phase 2 clinical trial
Source: Clin Transl Allergy. 2025 Jun 22;15(6):e70074. doi: 10.1002/clt2.70074 (PMC12183111; doi:10.1002/clt2.70074)
Supplement: Supplementary file 4 — Appendix D [file CLT2-15-e70074-s003.docx]

**Appendix D. Inclusion and exclusion criteria.**

**Inclusion Criteria**

**Participants who meet all of the following criteria may be included in this clinical trial：**

1. Aged 18-65 years at the time of screening, with no restrictions on gender;.
2. Diagnosed with allergic rhinitis according to the "Chinese Guidelines for the Diagnosis and Treatment of Allergic Rhinitis (2022, revised edition)": a) **Symptoms**: Two or more symptoms including paroxysmal sneezing, clear nasal discharge, nasal itching, and nasal congestion, with symptoms lasting or accumulating for more than 1 hour each day. Eye symptoms may also be present, such as itching, tearing, redness, and burning sensation. b)**Signs:** Common signs include pale and edematous nasal mucosa and watery nasal secretions. c) **Allergen Testing:** Positive for at least one allergen in a skin prick test (SPT) and/or serum specific IgE test, or a positive nasal provocation test (acceptable within 12 months prior to screening).
3. Unsatisfactory symptom control under standard-of-care in two consecutive years in the past(self-reported). During the pollen season, despite using a nasal glucocorticoid recommended by the guidelines or combined with an antihistamine, the total nasal symptom score remains ≥ 6, with nasal congestion ≥ 2.
4. Prior to randomization, participants must have experienced nasal symptoms for ≥ 2 days, or nasal and eye symptoms for ≥ 1 day, and the total nasal symptom score must be ≥ 1.
5. Male participants and their partners or female participants must agree to use one or more non-drug contraceptive methods (such as complete abstinence, intrauterine device, partner sterilization, etc.) during the trial and for 6 months after the trial ends, with no plans to donate sperm or eggs.
6. Agree to participate in this clinical trial and voluntarily sign the informed consent form.

**Exclusion Criteria**

**Participants who meet any of the following criteria cannot be included in this clinical trial：**

1. Allergic to the investigational drug or its excipients.
2. Coexisting conditions such as drug-induced rhinitis, vasomotor rhinitis, non-allergic rhinitis with eosinophilia syndrome, acute or chronic rhinosinusitis, dry rhinitis, atrophic rhinitis, severe nasal septum deviation, bronchial asthma, or previous asthma attacks during allergy season that required corticosteroids (mild, exercise-induced asthma not requiring medication or only β-agonist treatment during the study may be included).
3. Patients with perennial allergic rhinitis (seasonal allergic rhinitis complicated with perennial allergic rhinitis and present with seasonal episodes may be included).
4. Any nasal or sinus surgery within one year prior to screening.
5. Presence of glaucoma, cataracts, simple ocular herpes, infectious conjunctivitis, or other eye infections (excluding allergic conjunctivitis).
6. Unresolved and ongoing treatment-requiring local or systemic fungal, bacterial, viral, or parasitic infections, or oral candidiasis within four weeks before screening.
7. Clinically significant conditions (judged by investigators) include but not limited to unstable ischemic heart disease, NYHA Class III/IV left ventricular failure, arrhythmia, uncontrolled hypertension, cerebrovascular disease, neurodegenerative diseases, or other neurological disorders, uncontrolled hypo- or hyperthyroidism, other autoimmune diseases, hypokalemia, high adrenal status; past diagnosis of malignant tumors (except basal cell carcinoma or squamous cell skin cancer); history of myocardial infarction within 12 months before screening.
8. Laboratory Abnormalities at Screening: White blood cell count < 2.5 × 10^9/L; AST or ALT > 2.0 × ULN or total bilirubin > 1.5 × ULN; estimated glomerular filtration rate (eGFR) < 55 mL/min/1.73 m².
9. Treatment with similar experimental drugs (e.g., omalizumab) within six months before screening.
10. Use of systemic corticosteroids within four weeks before screening.
11. Use of intranasal corticosteroids, mast cell stabilizers, tricyclic antidepressants, leukotriene receptor antagonists, antihistamines within one week before randomization.
12. Use of traditional Chinese medicine for allergic rhinitis within seven days before randomization.
13. Use of allergen immunotherapy within six months before screening (for those who have not completed immunotherapy) or within three years before screening (for those who have completed immunotherapy).
14. Inability to discontinue use of anticholinergics (oral and intranasal), leukotriene receptor antagonists, antihistamines, mast cell stabilizers, decongestants, nasal saline rinses, tricyclic antidepressants, anti-allergy herbs, immunosuppressants/immunomodulators, immunotherapy, except for standard concomitant treatment and rescue medication as specified in the protocol.
15. Severe dysfunction of the heart, lungs, liver, or kidneys.
16. Poor compliance, such as poor medication adherence, inability to correctly fill out the diary card, or use of prohibited medications.
17. Comorbid neurological or psychiatric disorders that hinder cooperation or willingness to cooperate; legally defined disabilities (blindness, deafness, muteness, intellectual disability, mental disorders, etc.).
18. Plans to travel outside the local area to non-pollen areas for more than two consecutive days or a total of more than three days during the trial period.
19. Pregnant, breastfeeding, or planning to conceive soon.
20. Participation in another clinical drug trial within the last three months.
21. Any other condition that the investigator believes would make the participant unsuitable for the trial.
